# Use of Thiazide Diuretics and Risk of All Types of Skin Cancers: An Updated Systematic Review and Meta-Analysis

**DOI:** 10.3390/cancers14102566

**Published:** 2022-05-23

**Authors:** Surapon Nochaiwong, Mati Chuamanochan, Chidchanok Ruengorn, Kajohnsak Noppakun, Ratanaporn Awiphan, Chabaphai Phosuya, Napatra Tovanabutra, Siri Chiewchanvit, Manish M. Sood, Brian Hutton, Kednapa Thavorn, Greg A. Knoll

**Affiliations:** 1 Department of Pharmaceutical Care, Faculty of Pharmacy, Chiang Mai University, Chiang Mai 50200, Thailand; chidchanok.r@cmu.ac.th (C.R.); ratanaporn.a@cmu.ac.th (R.A.); chaba.pharmacy@gmail.com (C.P.); 2 Pharmacoepidemiology and Statistics Research Center (PESRC), Chiang Mai University, Chiang Mai 50200, Thailand; kajohnsak.noppakun@cmu.ac.th (K.N.); drsiri2010@gmail.com (S.C.); kthavorn@ohri.ca (K.T.); 3 Department of Internal Medicine, Division of Dermatology, Faculty of Medicine, Chiang Mai University, Chiang Mai 50200, Thailand; napatra.to@cmu.ac.th; 4 Department of Internal Medicine, Division of Nephrology, Faculty of Medicine, Chiang Mai University, Chiang Mai 50200, Thailand; 5 Department of Medicine, Division of Nephrology, University of Ottawa, Ottawa, ON K1H 8L6, Canada; msood@toh.ca (M.M.S.); gknoll@toh.ca (G.A.K.); 6 Ottawa Hospital Research Institute, Ottawa Hospital, Ottawa, ON K1H 8L6, Canada; bhutton@ohri.ca; 7 ICES uOttawa, Ottawa, ON K1Y 4E9, Canada; 8 School of Epidemiology and Public Health, Faculty of Medicine, University of Ottawa, Ottawa, ON K1G 5Z3, Canada

**Keywords:** basal cell carcinoma, keratinocyte carcinoma, melanoma, meta-analysis, skin cancers, squamous cell carcinoma, thiazide diuretics

## Abstract

**Simple Summary:**

By examining evidence from 30 non-randomized studies, we found that thiazide diuretic users have a higher risk of skin cancer than non-thiazide diuretic users. A small but consistent risk was identified across all types of skin cancers, including the more dangerous malignant melanoma (all subtypes, superficial spreading melanoma, nodular melanoma, and lentigo maligna melanoma) and non-melanoma skin cancers (basal cell carcinoma and squamous cell carcinoma). The high cancer risk associated with thiazides, especially the risk of malignant melanoma, is concerning. Individual skin cancer risk assessment, monitoring, and consideration of suitable drug alternatives are needed before the long-term use of these agents. Our findings suggest that to promote the rational use of anti-hypertensive medication, the benefits of thiazide diuretics must be weighed against potential safety concerns in terms of skin cancer risks.

**Abstract:**

Background: The use of thiazide diuretics is associated with skin cancer risk; however, whether this applies to all skin cancer types is unclear. Methods: In this meta-analysis, we searched multiple electronic databases and gray literature up to 10 April 2022, with no language restrictions, to identify relevant randomized controlled trials (RCTs) and non-randomized studies (cohort, case-control) that investigated the association between thiazide diuretics and skin cancer. The primary outcomes of interest were malignant melanoma and non-melanoma skin cancer (basal cell carcinoma [BCC], squamous cell carcinoma [SCC]). Secondary outcomes included other skin cancers (lip cancer, Merkel cell carcinoma, malignant adnexal skin tumors, oral cavity cancer, and precursors of skin cancer). We used a random-effects meta-analysis to estimate pooled adjusted odds ratios (ORs) and 95% confidence intervals (CIs). Results: Thirty non-randomized studies (17 case-control, 13 cohort, no RCTs) were included. Thiazide diuretic users had a higher risk of malignant melanoma (17 studies; *n* = 10,129,196; pooled adjusted OR, 1.10; 95% CI, 1.04–1.15; *p* < 0.001; strength of evidence, very low; very small harmful effect), BCC (14 studies; *n* = 19,780,476; pooled adjusted OR, 1.05; 95% CI, 1.02–1.09; *p* = 0.003; strength of evidence, very low; very small harmful effect), and SCC (16 studies; *n* = 16,387,862; pooled adjusted OR, 1.35; 95% CI, 1.22–1.48; *p* < 0.001; strength of evidence, very low; very small harmful effect) than non-users. Thiazide diuretic use was also associated with a higher risk of lip cancer (5 studies; *n* = 161,491; pooled adjusted OR, 1.92; 95% CI, 1.52–2.42; *p* < 0.001; strength of evidence, very low; small harmful effect), whereas other secondary outcomes were inconclusive. Conclusions: Thiazide diuretics are associated with the risk of all skin cancer types, including malignant melanoma; thus, they should be used with caution in clinical practice.

## 1. Introduction

Numerous classes of anti-hypertensive medications are available to treat high blood pressure. Thiazide diuretics are commonly prescribed anti-hypertensive medications, accounting for approximately 30% of all prescriptions in the United States and Western Europe [1,2]. Owing to their efficacy for primary and secondary cardiovascular or cerebrovascular protection, thiazide diuretics are widely used among individuals with essential hypertension or in conjunction with other anti-hypertensive medications in severely hypertensive patients, as well as in patients with stroke or transient ischemic attack [3].

Emerging evidence from post-marketing surveillance studies suggests that thiazide diuretics can increase the risk of skin cancer [4]. In the United States, the health and economic burden of skin cancer is dramatically increasing, with approximately 5 million people being treated for skin cancer at the cost of $8.1 billion [5]. Thiazides have photosensitizing properties, with a chemical structure that promotes the absorption of ultraviolet radiation [6]. In 2020, the United States Food and Drug Administration (FDA) approved changes to the product monograph for hydrochlorothiazide to reflect a small increased risk of non-melanoma skin cancer (basal cell carcinoma [BCC], squamous cell carcinoma [SCC]) [7].

Among the skin cancers, melanomas are the deadliest. They exhibit an early metastatic potential and are often highly aggressive, though whether the risk associated with thiazides differs according to skin cancer types is unclear [4,8,9,10,11]. Existing clinical trials did not report cancer events; however, these studies were based on a limited follow-up period. Several post-marketing surveillance and observational studies have investigated the association between thiazide diuretics and skin cancer in real-world settings. Such real-world studies provide greater generalizability and power of the findings, supplementing evidence from randomized controlled trials (RCTs). To close the evidence gaps in carcinogenic safety, we aimed to summarize all available real-world evidence on the association between thiazide diuretic use and the risk of all skin cancer types.

## 2. Materials and Methods

This meta-analysis followed the Preferred Reporting Items for Systematic Reviews and Meta-Analyses guidelines [12] and the Meta-analysis of Observational Studies in Epidemiology statement (Supplementary Files S1 and S2 [13]. The study protocol was registered in the International Prospective Register of Systematic Reviews (CRD42020220848). The pre-specified protocol was amended and described in Supplementary File S3.

### 2.1. Systematic Literature Search

We searched electronic databases including Medline, Embase, PubMed, Cochrane Library, Web of Science, Scopus, and CINAHL from inception to 7 May 2021, with no language restrictions. Combinations of Medical Subject Headings and search terms were used, including pharmacological class or individual drugs (i.e., “diuretics” or “thiazides” or “hydrochlorothiazide” or “HCTZ”), and skin cancers (i.e., “skin neoplasm” or “melanoma” or “non-melanoma” or “keratinocyte cancer” or “basal cell carcinoma” or squamous cell melanoma”). The full search strategy for each database is provided in Supplementary Table S1. Grey literature from Google Scholar and preprints (medRxiv, bioRxiv) were also searched. Moreover, potentially relevant articles were manually searched from prior systematic reviews, reference lists of the included studies, and major international pharmacoepidemiology/dermatology/oncology scientific meetings. An updated search was conducted up to 10 April 2022.

### 2.2. Study Selection Criteria and Outcomes

We included both RCTs and non-randomized studies (cohort, case-control) that (i) investigated thiazide diuretic use for any indication and the risk of skin cancer among individuals aged 12 years or older; (ii) consisted of two or more groups, with one group representing users of thiazide or thiazide-type diuretics (i.e., bendroflumethiazide, chlorthalidone, hydrochlorothiazide, hydroflumethiazide, indapamide, metolazone, or thiazides in combination with other anti-hypertensive medications); and (iii) reported the occurrence of any type of skin cancer. We excluded studies that (i) reported only unadjusted effect estimates or lacked information to calculate risk estimates; (ii) had a small sample size (less than 50 patients); and (iii) were case series/case reports, N-of-one trials, pharmacokinetic/pharmacodynamic studies, cross-sectional studies, RCTs, and reviews. Details of the selection criteria are described in Supplementary Table S2. For potential articles with overlapping study periods or populations, we combined relevant information or selected the most detailed study.

The primary outcomes of interest were the major skin cancer types: (i) malignant melanoma and specific subtypes (superficial spreading melanoma, nodular melanoma, lentigo maligna melanoma); and (ii) non-melanoma skin cancer (BCC, SCC, and unspecified non-melanoma). The secondary outcomes were other forms of skin cancer (lip cancer, Merkel cell carcinoma [MCC], malignant adnexal skin tumor [MAST], oral cavity cancer, precursor of skin cancer [actinic keratosis]).

### 2.3. Study Selection, Data Collection, and Risk of Bias Evaluation

Initially, two investigators (SN, MC) independently screened eligible articles based on the titles and abstracts of records identified through systematic searches. Thereafter, a full-text review was conducted to identify the final set of studies for inclusion. Potentially eligible articles published in languages other than English were translated before full-text assessment. Any discrepancies in study selection at either stage were resolved through a team discussion.

Using a standardized approach, two investigators (SN, MC) independently extracted information about (i) study characteristics, including study design (case-control, cohort), sample size, study population and setting, study period, statistical analysis methods (multivariable or propensity score approach), and risk factors adjusted for when deriving effect estimates; (ii) patient characteristics (mean or median age of the study population, the proportion of female participants, comorbidities and skin conditions, and concomitant medications including photosensitive agents); and (iii) specific exposure and control groups, and predefined outcomes of interest (definition of thiazide diuretic users and non-users, dosage and duration of exposure, and skin cancer case ascertainment definition and methods). For studies with incomplete or unclear information, the first or corresponding authors were contacted for clarification. If the authors did not reply after two attempts, we excluded their study from the meta-analyses. The final data set was independently cross-checked by two investigators (RA, CP) to resolve any discrepancies.

Two investigators (CR, KN) independently assessed the methodological quality of each study using the Cochrane risk of bias in randomized trials (RoB 2) [14] and Newcastle-Ottawa Scale (NOS) for non-randomized studies (Supplementary File S4) [15]. For randomized trials, the included studies were then classified as low, high, or of some concern. The NOS scores ranged from 0–9, with higher scores indicating higher overall quality. Considering the overall risk of bias, a study was classified as having the highest quality if the NOS summary score was 8 or more points [16,17].

### 2.4. Approach to Evidence Synthesis

All analyses were performed, and forest plots were created using Stata software (version 16.0; StataCorp, College Station, TX, USA). We used adjusted odds ratios (ORs) with the greatest degree of confounder adjustment in meta-analyses assessing the association between thiazide diuretic use and the outcomes of interest. Since the methodological approach varied across included studies, we used the random-effects model to estimate pooled adjusted ORs with 95% confidence intervals (CIs) to address heterogeneity across all included studies [18]. Using the random-effects model, we calculated 95% prediction intervals for each outcome of interest to account for a predicted range and the expected uncertainty about the estimate of a future study [19]. We also estimated the expected (E)-value to address the robustness of the identified association between thiazide diuretics and skin cancer risk to potential unmeasured confounders [20].

Evidence of statistical heterogeneity was evaluated using the Cochran *Q* test, with a *p*-value of less than 0.100 indicating significant heterogeneity. The *I^2^* index and tau-squared (*τ*^2^) statistics were also used to classify the degree of heterogeneity as low (*I^2^* = 25.0%, *τ*^2^ = 0.01), moderate (*I^2^* = 50.0%, *τ*^2^ = 0.06), or high (*I^2^* = 75.0%, *τ*^2^ = 0.16) [21,22]. When applicable, funnel plots for each outcome of interest were visualized to investigate the asymmetry of the funnel graph. Statistical publication bias was assessed using Begg’s and Egger’s tests, with a *p*-value of less than 0.100 indicating significant publication bias [23,24]. To account for publication bias and address the number of included studies with null effects, the trim-and-fill method was also applied [25].

### 2.5. Subgroup and Sensitivity Analyses

*A priori* subgroup analyses were planned to determine (i) patient characteristics (age, sex, race/ethnicity, history of chronic skin diseases, skin conditions [history of naevi, precancerous skin lesions, Fitzpatrick skin type]), ultraviolet radiation exposure, use of photosensitive agents, immunosuppressant, renin-angiotensin-system inhibitors, and non-steroidal anti-inflammatory drugs; and (ii) study characteristics (sample size [less than 10,000 vs. 10,000 or more]), study design (case-control vs. cohort), and study location (Europe/North America vs. international/other). If possible, individual thiazide diuretic use, dosage, and duration of exposure were also used to investigate the evidence for dose-response and duration-response relationships.

To assess the robustness of the findings, sensitivity analyses were also performed by (i) including only studies with an NOS score of 8 or greater (i.e., highest quality studies), (ii) excluding studies that analyzed patients with a known risk of skin cancer development (i.e., organ transplant recipients), (iii) incorporating unpublished conference abstracts into the main analysis (post-hoc sensitivity analysis), and (iv) removing individual studies (i.e., leave-one-out analysis). Moreover, to investigate the effects of pre-specified covariates on risk estimates, we performed a univariate meta-regression based on the risk-of-bias level, study characteristics, and patient characteristics in the random-effects meta-analysis model.

### 2.6. Judging the Strength of Evidence

The strength of a body of evidence across all types of skin cancers was independently assessed by two clinicians (MC, SC) and two methodologists (SN, CR) using the modified guidance of Grading of Recommended Assessment, Development, and Evaluation (GRADE) working group [26] along with the United State Agency for Healthcare Research and Quality (AHRQ) for the Evidence-based Practice Center (EPC) program (Supplementary File S5) [27]. Evidence certainty was classified into insufficient data or very low, low, moderate, or high-quality evidence [26,27]. Finally, to draw conclusions based on an integrated clinical context and a methodological approach, we summarized the treatment effects of thiazide diuretics concerning the risk of skin cancer types as trivial (not substantially different from non-use of thiazide diuretics), harmful, or beneficial. To estimate the magnitude of the effect of thiazide diuretic use on a particular outcome, we classified the pooled risk estimates as very small (OR, less than 1.68), small (OR, 1.68 to 3.46), medium (OR, 3.47 to 6.71), or large (OR, greater than 6.71) [28].

## 3. Results

### 3.1. Evidence Identified from the Search

The systematic search identified 2854 records (Supplementary Figure S1). From these, 501 duplicate records were removed, and 2353 records remained. Screening titles and abstracts identified 90 potentially relevant citations (Supplementary File S6). However, no RCTs fulfilled the study selection criteria due to a lack of information for pooling the risk estimates according to a specific type of skin cancer [4]. Of these, 30 non-randomized studies (17 case-control, 13 cohort) published in full-text form fulfilled the study selection criteria and were included in the review [29,30,31,32,33,34,35,36,37,38,39,40,41,42,43,44,45,46,47,48,49,50,51,52,53,54,55,56,57,58]. Furthermore, two unpublished conference abstracts were included in the post-hoc sensitivity analysis [59,60].

### 3.2. Overview of Study Characteristics

The included studies were reported between 1996 and 2021 from Europe (Denmark, Finland, France, Germany, Greece, Iceland, Italy, Malta, Netherlands, Poland, Scotland, Spain, Sweden, UK), North America (Canada, USA), Australia, and Asia (Korea, Taiwan). Table 1 describes the study characteristics. The mean age of the study participants ranged from 49.0 to 80.7 years, and most of the included studies were conducted in the elderly population. The proportion of female participants ranged from 26.4% to 63.5%. Hydrochlorothiazide was the thiazide diuretic used in most studies (19 studies, 63.3%). Detailed skin cancer case ascertainment, methodology, and statistical methods for data analysis; comorbidities and skin conditions of the participants; and co-medication use in the included studies are provided in Supplementary Tables S3–S6. Among the 30 non-randomized studies, malignant melanoma was the most common study outcome (*n* = 17) [29,30,31,33,37,38,41,44,45,46,48,49,52,54,56,57,58], followed by SCC (*n* = 16) [30,31,33,36,37,38,40,42,47,48,50,51,53,56,57,58], BCC (*n* = 14) [30,31,32,33,37,38,40,47,48,50,53,56,57,58], and unspecified non-melanoma (*n* = 6) [44,46,49,52,55,56]. With respect to secondary outcomes, five studies reported on lip cancer [34,39,44,45,48], two studies reported on MCC [31,43], and only one study each reported on MAST [43], oral cavity cancer [48], and actinic keratosis [35]. Based on the assessed risk of bias (Supplementary Table S7), the summary NOS scores ranged from 5 to 9 points, with 20 studies (66.7%) having high quality (NOS of 8 or greater).

### 3.3. Finding from Meta-Analysis

**Figure 1:**
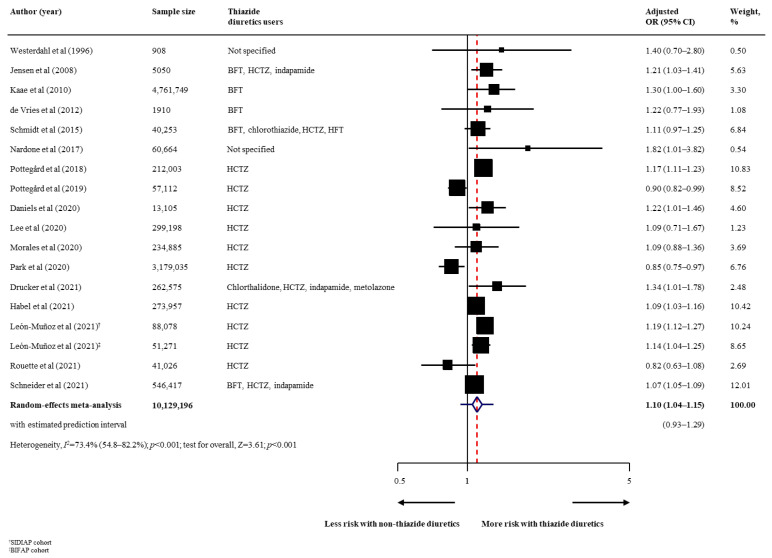
Effect of the use of thiazide diuretics and the risk of malignant melanoma. Abbreviations: BFT, bendroflumethiazide; CI, confidence interval; HCTZ, hydrochlorothiazide; HFT, hydroflumethiazide; OR, odds ratio [29,30,31,33,37,38,41,44,45,46,48,49,52,54,56,57,58].

**Figure 2:**
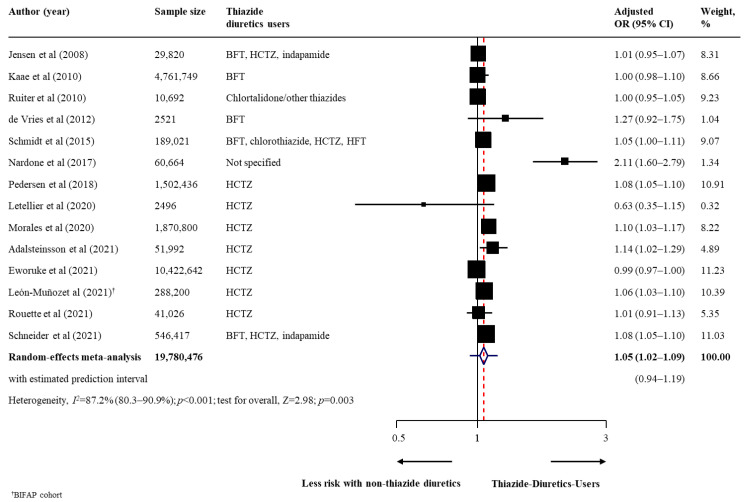
Effect of the use of thiazide diuretics and the risk of basal cell carcinoma. Abbreviations: BFT, bendroflumethiazide; CI, confidence interval; HCTZ, hydrochlorothiazide; HFT, hydroflumethiazide; OR, odds ratio [30,31,32,33,37,38,40,47,48,50,53,56,57,58].

**Figure 3:**
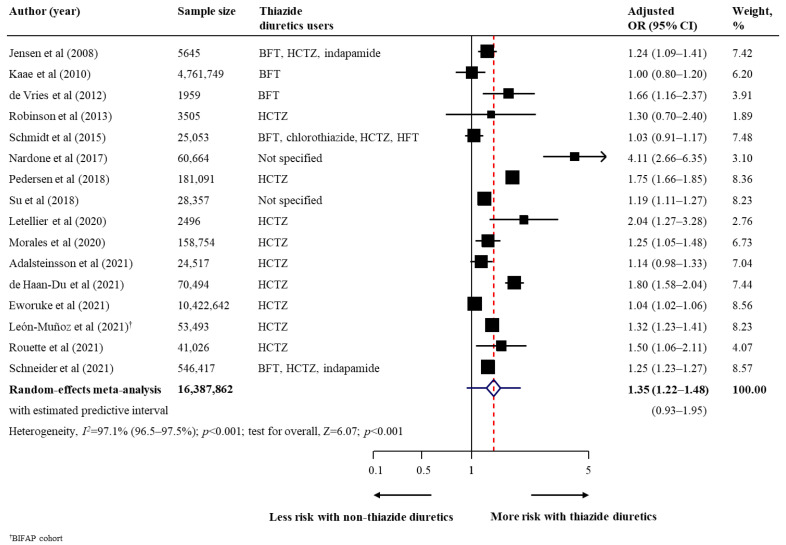
Effect of the use of thiazide diuretics and the risk of squamous cell carcinoma. Abbreviations: BFT, bendroflumethiazide; CI, confidence interval; HCTZ, hydrochlorothiazide; HFT, hydroflumethiazide; OR, odds ratio [30,31,33,36,37,38,40,42,47,48,50,51,53,56,57,58].

**Figure 4:**
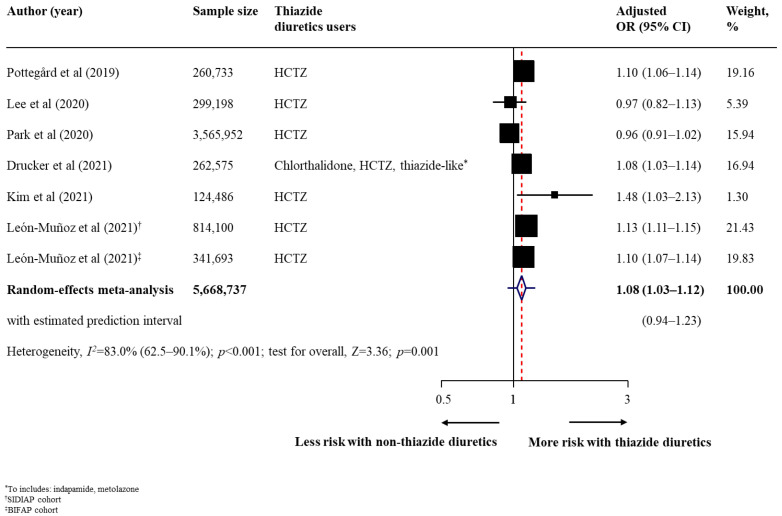
Effect of using thiazide diuretics and the risk of unspecified non-melanoma skin cancer. Abbreviations: CI, confidence interval; HCTZ, hydrochlorothiazide; OR, odds ratio [44,46,49,52,55,56].

The summary findings, strength of evidence, and conclusion on the association between thiazide diuretic use and risk of all skin cancer types are shown in Table 2. Regarding the primary outcomes, thiazide diuretic users had a statistically higher risk of malignant melanoma (17 studies [29,30,31,33,37,38,41,44,45,46,48,49,52,54,56,57,58]; *n* = 10129196; pooled adjusted OR, 1.10; 95% CI, 1.04–1.15; *p* < 0.001; moderate heterogeneity [*I*^2^ = 73.4%]; Figure 1) than to non-users. In a fewer number of studies (3 studies), thiazide diuretic users also had a higher risk of all subtypes of malignant melanoma, with pooled adjusted ORs of 1.18 (95% CI, 1.05–1.33) for superficial spreading melanoma, 1.23 (95% CI, 1.08–1.40) for nodular melanoma, and 1.33 (95% CI, 1.08–1.65) for lentigo maligna melanoma (Table 2). For non-melanoma skin cancer, thiazide diuretic users had an increased risk of BCC (14 studies [30,31,32,33,37,38,40,47,48,50,53,56,57,58]; *n* = 19780476; pooled adjusted OR, 1.05; 95% CI, 1.02–1.09; *p* = 0.003; high heterogeneity [*I*^2^ = 87.2%]; Figure 2), SCC (16 studies [30,31,33,36,37,38,40,42,47,48,50,51,53,56,57,58]; *n* = 16387862; pooled adjusted OR, 1.35; 95% CI, 1.22–1.48; *p* < 0.001; high heterogeneity [*I*^2^ = 97.1%]; Figure 3), and unspecified non-melanoma (6 studies [44,46,49,52,55,56]; *n* = 5668737; pooled adjusted OR, 1.08; 95% CI, 1.03–1.12; *p* = 0.001; high heterogeneity [*I*^2^ = 83·0%]; Figure 4). With respect to secondary outcomes, thiazide diuretic users had an increased risk of lip cancer (5 studies [34,39,44,45,48]; *n* = 161491; pooled adjusted OR, 1.92; 95% CI, 1.52–2.42; *p* < 0.001; moderate heterogeneity [*I*^2^ = 51.5%]; Supplementary Figure S2). Other skin cancer forms (MCC, MAST, oral cavity cancer, and actinic keratosis) were inconclusive owing to limited evidence (Table 2).

### 3.4. Subgroup and Sensitivity Analyses

*A priori* subgroup analyses based on participant characteristics, secondary outcomes, and dose- and duration-response relationships could not be performed because of limited details on diuretic use, skin conditions, and ultraviolet exposure. However, the risk among thiazide diuretic users appeared in individuals who took hydrochlorothiazide, studies with a sample size of more than 10,000, case-control studies, and studies conducted in Europe and North America (Supplementary Table S8). After a post-hoc sensitivity analysis, our findings were robust and did not significantly differ from the main results (Supplementary Tables S9–S12). In univariable meta-regression, the study location (particularly in Europe and North America) and mean age of study participants were associated with heterogeneity in the risk estimates for malignant melanoma and unspecified non-melanoma, respectively (Supplementary Table S13). No publication bias was observed in any of the outcomes of interest in Begg’s and Egger’s tests and visual inspection of funnel plots (all *p*-value more than 0.100; Supplementary Table S14 and Figure S3).

### 3.5. Evidence Certainty

The quality of evidence for each outcome of interest according to the modified GRADE approach is described in Supplementary Table S15. Based on the strength of evidence, effect size magnitude, evidence certainty, and potential unmeasured confounders, we graded and classified the association between thiazide diuretic use and risk of malignant melanoma and non-melanoma skin cancer as having a low strength of evidence with a very small harmful effect. Meanwhile, thiazide diuretic use and lip cancer had a small harmful effect with a very low strength of evidence. Other forms of skin cancer were judged to have insufficient data (Table 2).

## 4. Discussion

Thiazide diuretics are commonly used blood pressure-lowering agents associated with skin cancer risk; however, whether the risk differs according to skin cancer types is unclear. We summarized the evidence from 30 non-randomized studies that analyzed the data of up to 19 million individuals on the association between thiazide diuretics and the risk of all skin cancer types. We found that thiazide diuretic users had a higher risk of all cancer types, including malignant melanoma and non-melanoma skin cancer (very small to small effect, low certainty of evidence).

The International Agency for Research on Cancer has classified hydrochlorothiazide as a possible carcinogenic medication (group 2B) [61]. Subsequently, the FDA has changed the product labeling for hydrochlorothiazide to reflect the risk of non-melanoma skin cancer [7]. Theoretically, thiazide diuretics may increase the risk of skin cancers through the ultraviolet-induced dissociation of their chlorine substitute, leading to free radical formation, DNA damage, and chronic subclinical skin inflammation [6]. Some epidemiological studies found an association between thiazide diuretic use, particularly among hydrochlorothiazide users, and the risk of SCC and lip cancer in terms of dose- and duration response patterns (i.e., cumulative usage of 50,000 mg or greater and duration of use of 5 years or over) [39,40,53], whereas others did not [48,54,56]. Unfortunately, our analyses cannot confirm these dose- and duration-response relationships owing to limited information and heterogeneous definitions of thiazide diuretic users.

Concerning previous meta-analyses of non-randomized studies, the study by Gandini et al. [8], which included six non-randomized studies, found no skin cancer risk (malignant melanoma, BCC, and SCC) among thiazide diuretic users. Conversely, another three reports by Tang et al. [9,10] (two unique meta-analyses with 10 non-randomized studies) and Bendinelli et al. [11] (nine non-randomized studies and one conference abstract) revealed a significantly higher risk of malignant melanoma (risk estimate, 1.10 to 1.17), BCC (risk estimate, 1.10 to 1.17), and SCC (risk estimate, 1.40 to 1.93) among thiazide diuretic users. Compared with existing systematic reviews and meta-analyses, our study expanded the risk estimates across all types of skin cancers and updated the contemporary evidence by adding 30 non-randomized studies and by using a comprehensive methodological approach.

In 2021, Copland et al. [4] performed a meta-analysis of individual patient data from six RCTs (*n* = 58,185) comparing thiazide diuretics with other anti-hypertensive medications, with trial durations of 4.5 years (interquartile range, 3.7–5.5 years). They found that thiazide diuretic use was associated with an increased risk of cancer death (hazard ratio, 1.14; 95% CI, 1.03–1.26); however, no evidence of association was recognized in the incidence of unspecified type of skin cancer. In fact, apart from outcomes with delayed onsets, such as cancer-related treatment interventions, RCTs are generally not intended to assess the long-term carcinogenic safety profiles of anti-hypertensive medications. Meanwhile, our study has an important methodological contribution because we included real-world evidence from a large sample with diverse participant characteristics and a long follow-up time, which allowed us to quantify the risk of all types of skin cancers associated with thiazide diuretic use in clinical practice. Interestingly, the risk of skin cancer associated with thiazide diuretics seemed to be limited to Europe and North America, as the association was not observed in other regions (Taiwan or Korea). We postulated that Europeans and North Americans, who are mostly fair-skinned, are more susceptible to ultraviolet radiation and the photosensitizing properties of thiazide diuretics. This finding is supported by a systematic review by Lopes et al. [62] which included “colored” populations (i.e., Africans, Asians, Pacific Islanders, indigenous populations, Hispanics) and showed no or weak relationship between ultraviolet radiation exposure and cutaneous melanoma. Nevertheless, further studies in non-white populations are needed to fully clarify this association.

### 4.1. Strengths and Limitations

Our study included up-to-date and expanded evidence on the association between thiazide diuretic use and the risk of all skin cancer types. From a methodological viewpoint, we performed extensive searching without language restrictions along with a rigorous and comprehensive systematic review to draw evidence-based conclusions. Moreover, the results were robust based on sensitivity analyses and no evidence of publication bias.

Nevertheless, our meta-analysis had several limitations. First, since our results are based on non-randomized studies, the risk of selection bias in terms of confounding by indication or contraindication should be considered when interpreting our findings. Therefore, conclusions about causality relationships cannot be drawn. Second, apart from the nature of non-randomized studies, we found that uncertainty and potential unmeasured confounders existed based on the prediction interval and E-value, respectively. Consequently, we downgraded and judged the strength of evidence to be very low. Third, in the risk-of-bias assessment, only 20 studies (66.7%) were of high quality (NOS of 8 or greater). However, a post-hoc sensitivity analysis, including studies with the highest quality, showed no substantial difference from the main findings. Fourth, our findings may have information bias because most of the included studies relied on electronic databases or routinely collected administrative data. Nonetheless, most studies identified skin cancer cases using histological confirmation or through regional or national cancer registries. Fifth, differences were observed in the study population and exposure to individual thiazide diuretics, which could explain the moderate or high heterogeneity of the pooled effect estimates. Moreover, the results were incompletely adjusted for known risk factors, namely ultraviolet radiation exposure. Different skin phenotypes such as fair or colored skin were addressed only in a few included studies. Lastly, details on individual thiazide diuretic use and key participant characteristics (i.e., skin conditions or phenotypes, degree of ultraviolet radiation exposure, and use of other photosensitive agents) are lacking; thus, risk estimates in subpopulations cannot be derived.

### 4.2. Implications for Practice and Future Research

Given the limited effect size and evidence certainty, our findings provide the best available evidence on the carcinogenic safety of thiazide diuretics in general practice. Although thiazide diuretic use only slightly increases the risk of skin cancers, our study underscores that the benefits and risks of this medication class should be balanced, especially with long-term use in the management of chronic conditions such as hypertension. Proactive monitoring is warranted in thiazide diuretic users, and those at risk of developing skin cancer should be identified. These include individuals with extreme exposure to ultraviolet radiation from sunlight, indoor tanning, or other artificial sources for medical or cosmetic purposes; fair skin and advanced age; a history of skin cancer or precancerous skin conditions; and immunocompromised conditions or use of immunosuppressive agents that may further increase the skin cancer risk [63,64,65].

To minimize skin cancer risk and promote rational drug use, physicians should encourage patients to use sunscreen and sun protection in an outdoor setting and reduce the use of indoor tanning or other artificial radiation sources, as well as provide information about sun safety when prescribing thiazide diuretics. Further collaborative longitudinal pharmacoepidemiological surveillance studies using real-world data are needed to confirm the causal association between thiazide diuretic use and skin cancer risk. Moreover, proactive screening studies and intervention trials focusing on skin cancer prevention strategies are warranted.

## 5. Conclusions

With respect to the very low strength of evidence certainty, thiazide diuretics are associated with the risk of skin cancer, including malignant melanoma and non-melanoma skin cancer. Future studies examining other forms of skin cancers or individual thiazide diuretics, as well as dose- and duration-response relationships, are required. Our findings suggest that individual skin cancer risk assessments, consideration of suitable alternative medications, and limited duration of thiazide diuretic use may be potential mitigation strategies.

## Figures and Tables

**Table 1:** Characteristics of the included studies in the meta-analysis.

Author (year)	Country	Study Design	Total Sample Size	Study Population	Database	Study Period	Age in Years, Mean ± SD	Female Sex, No. (%)	Definition of Thiazide Diuretics Exposure	Outcomes Reported: Skin Cancer
Westerdahl et al. (1996) [29]	Sweden	Nested case-control	908	Participants in the South Swedish Healthcare region	Regional cancer registry	1 July 1988–30 June 1990	Range: 15–75 (NS)	NR	Self-reported: used prescribed thiazide diuretics > 1 month continuously	MM
Jensen et al. (2008) [30]	Denmark	Nested case-control	BCC cohort, 29,820; SCC cohort, 5645; MM cohort, 5050	Adult Danish residents in North Jutland country	EHRs linkage with cancer registry	1989–2003	BCC cohort, median 69; SCC cohort, median 77; MM cohort, median 59	NR	Thiazide/Thiazide-like-users (bendroflumethiazide, indapamide, HCTZ): any prescriptions filled, >1 year, and >5 years before the index date	BCC, SCC, MM
Kaae et al. (2010) [31]	Denmark	Retrospective cohort	4,761,749	Danish residents	EHRs linkage with cancer registry	1995–2006	≥15 (NS)	NR	Bendroflumethiazide users: filled at least 1 prescription	BCC, SCC, MCC, MM
Ruiter et al. (2010) [32]	Netherlands	Prospective cohort	10,692	Adult participants in the Rotterdam cohort (mainly Caucasians)	Rotterdam Study I and II; National registry of histo- and cytopathology	1 April 1991–31 December 2007	69.0 (9.7)	6404 (59.9)	Thiazide diuretics-users (chlorthalidone and thiazides in combination with other drugs)	BCC
de Vries et al. (2012) [33]	International	Case-control	2521	Hospital-based adult European populations in Finland, Germany, Greece, Italy, Malta, Poland, Scotland, and Spain	EPIDERM consortium, partly self-administered and partly completed by dermatologists	NR	67.1 (12.1)	1464 (58.1)	Thiazide diuretics-users (self-reported)	BCC, SCC, MM
Friedman et al. (2012) [34]	USA	Case-control	23,616	Adult non-Hispanic whites in the San Francisco Bay area and central valley of California	EHRs linkage with cancer registry—SEER program	1 August 1994–29 February 2008	67.2 (8.8)	6243 (26.4)	HCTZ-users	Lip cancer
Traianou et al. (2012) [35]	International	Case-control	1029	Hospital-based adult European populations in Finland, Germany, Greece, Italy, Malta, Poland, Scotland, and Spain	EPIDERM consortium, partly self-administered and partly completed by dermatologists	NR	65.4 (9.7)	382 (37.1)	Thiazide diuretics-users (self-reported)	Actinic keratosis
Robinson et al. (2013) [36]	USA	Nested case-control	Whole cohort, 5072; SCC cohort, 3505	Adult residents of New Hampshire, speak English	Part of New Hampshire Skin Cancer Study	July 1993–June 2009	≤60, 2285 (45.1%); 61–70, 1846 (36.4%); >70, 941 (18.6%)	2213 (43.6)	HCTZ-users (self-reported)	SCC
Schmidt et al. (2015) [37]	Denmark	Nested case-control	Whole cohort, 254,927; BCC cohort, 18,902; SCC cohort, 25,053; MM cohort, 40,253	Adult Danish residents in northern Denmark	EHRs linkage with cancer registry	1991–2010	<60, 86,892 (34.1%); 60–69, 62,721 (24.6%); 70–79, 59,680 (23.4%); ≥80, 45,634 (17.9%)	125,419 (49.2)	Thiazides diuretics-users (bendroflumethiazide, hydroflumethiazide, HCTZ, chlorothiazide)	BCC, SCC, MM
Nardone et al. (2017) [38]	USA	Retrospective cohort	60,664	Adult participants receiving treatment through Northwestern University healthcare affiliates	Northwestern Medicine Enterprise Data Warehouse	January 2004–December 2014	<60, 29,791 (49.1%); 60–69, 15,412 (25.4%); 70–79, 10,129 (16.7%); 80–89, 5306 (8.7%)	38,315 (63.2)	Thiazide diuretics-users (NS)	BCC, SCC, MM
Pottegård et al. (2017) [39]	Denmark	Nested case-control	63,700	Adults Danish residents	EHRs linkage with cancer registry	1 January 2004–31 December 2012	72.0 (11.9)	20,775 (32.6)	HCTZ-users	Lip cancer
Pedersen et al. (2018) [40]	Denmark	Nested case-control	BCC cohort, 1502436; SCC cohort, 181091	Adults Danish residents	EHRs linkage with cancer registry	1 January 2004–31 December 2012	BCC cohort, 66.3 (14.1); SCC cohort, 76.7 (12.6)	792,333 (52.7)	HCTZ-users	BCC, SCC
Pottegård et al. (2018) [41]	Denmark	Nested case-control	212,003	Adults Danish residents	EHRs linkage with cancer registry	1 January 2004–31 December 2015	Range:18–90	NR	HCTZ-users	MM
Su et al. (2018) [42]	USA	Retrospective cohort	28,357	Adult non-Hispanic white patients with hypertension in a closed healthcare system	Based on KPNC’s Research Program in Genes and Environmental Health	1 January 2002–31 December 2012	69.1 (10.6)	15,975 (56.3)	Thiazide-users	SCC (in situ or invasive)
Pedersen et al. (2019) [43]	Denmark	Nested case-control	MCC cohort, 1954; MAST cohort, 2752	Adult Danish residents	EHRs linkage with cancer registry	1 January 2004–31 December 2015	MCC cohort, 78.6 (11.9); MAST cohort, 71.0 (13.4)	MCC cohort, 1156 (59.2); MAST cohort, 1449 (52.6)	HCTZ-users	MCC, MAST
Pottegård et al. (2019) [44]	Taiwan	Nested case-control	319,902	Adult Taiwanese residents	NHIRD, Nationwide Taiwanese claims database	1 January 2008–31 December 2015	67.3 (20.0)	156,211 (48.8)	HCTZ-users	Non-melanoma skin cancer (lip and non-lip), MM
Daniels et al. (2020) [45]	Australia	Nested case-control	Lip cancer cohort, 911; MM cohort, 13,105	Elderly patients aged ≥65 years within a population of veterans residing in New South Wales	EHRs linkage with cancer registry	1 January 2004–31 December 2015	Lip cancer cohort, 78.3 (4.5); MM cohort, 80.7 (3.7)	Lip cancer cohort, 386 (42.4); MM cohort, 4263 (32.5)	HCTZ-users	Lip cancer (SCC), MM
Lee et al. (2020) [46]	Korea	Retrospective cohort	299,198	Adult patients aged 20–80 years	Three-academic center hospital-Based	1 January 2004–28 February 2018	59.7 (13.8)	157,655 (52.7)	HCTZ-users	Non-melanoma skin cancer (NS), MM
Letellier et al. (2020) [47]	France	Retrospective cohort	2496	Adult patients undergoing kidney, pancreas, or combined kidney-pancreas transplantation with graft functioned ≥ 3 months)	Single-center at university hospital	1 January 2000–31 December 2017	49.0 (14.0)	958 (38.4)	HCTZ-users	BCC, SCC
Morales et al. (2020) [48]	UK	Nested case-control	BCC cohort, 1,870,800; SCC cohort, 158,754; lip cancer cohort, 71,207; oral cavity cancer cohort, 73,844; MM cohort, 234,885	Population-based: adults aged ≥ 18 years	THIN database	1 January 1999–1 May 2016	SCC cohort, 74.8 (11.5); BCC cohort, 68.3 (13.6); lip cancer cohort, 63.8 (13.6); oral cavity cohort, 61.5 (13.2); MM cohort, 58.2 (16.4)	SCC cohort, 63,315 (39.9); BCC cohort, 913,647 (48.8); lip cancer cohort, 23,939 (33.6); oral cavity cancer cohort: 24,675 (33.4); MM cohort: 133,665 (56.9)	HCTZ-users	BCC, SCC, lip cancer, oral cavity cancer, MM
Park et al. (2020) [49]	Korea	Retrospective cohort	3,565,952	Population-based: adult patients aged ≥ 18 years with a first diagnosis of primary hypertension	Health Insurance Review and Assessment Service claims database	1 January 2007–30 June 2017	55.5 (12.8)	1,519,379 (47.8)	HCTZ-users	Non-melanoma skin cancer (NS), MM
Adalsteinsson et al. (2021) [50]	Iceland	Case-control	BCC cohort, 51,992; SCC in situ cohort, 13,128; invasive SCC cohort, 11,389	Population-based: all Icelandic population	EHRs linkage with cancer registry	2003–2017	BCC cohort, 68.0 (17.1); SCC in situ cohort 76.0 (12.6); invasive SCC: 78.2 (11.1)	BCC cohort, 29,982 (57.7); SCC in situ cohort, 8335 (63.5); invasive SCC cohort: 5559 (48.8)	HCTZ-users	BCC, SCC in situ, invasive SCC
de Haan-Du et al. (2021) [51]	Netherlands	Prospective cohort	70,494	Adult type 2 diabetes patients	EHRs linkage with cancer registry	1998–2019	66.5 (12.1)	34,949 (49.6)	HCTZ-users	SCC
Drucker et al. (2021) [52]	Canada	Retrospective cohort	262,575	Population-based: elderly aged ≥ 65 years	Linked administrative health data from Ontario	1 January 1998–31 December 2017	70.7 (5.9)	165,723 (63.1)	New users of thiazides: chlorthalidone, HCTZ, indapamide, metolazone	BCC, SCC, MM
Eworuke et al. (2021) [53]	USA	Retrospective cohort	10,422,642	Adults participants	US FDA Sentinel System (17 health plans)	1 January 2000–31 August 2018	60.7 (NS)	5,503,155 (52.8)	New users of any HCTZ-containing products	BCC, SCC
Habel et al. (2021) [54]	USA	Nested case-control	273,957	Adults non-Hispanic White participants	KPNC, an integrated healthcare system	1 January 1996–30 June 2014	<60, 112,049 (40.9%); 60–69, 67,788 (24.7%); ≥70, 94,120 (34.4%)	116,750 (42.6)	HCTZ-users	MM
Kim et al. (2021) [55]	Korea	Retrospective cohort	124,486	Adult participants randomly selected from 91% of people in the country	Korean National Health Insurance Service National Sample Cohort	2002–2013	<60, 65,214 (52.4%); 60–69, 32,762 (26.3%); ≥70, 26,510 (21.3%)	64,774 (52.0)	HCTZ-users (cumulative dose of ≥2500 mg)	Non-melanoma skin cancer (NS)
León-Muñoz et al. (2021) [56]: SIDIAP cohort	Spain	Nested case-control	Nonmelanoma cohort, 814,100; MM cohort, 88,078	Population-based: adults aged ≥ 18 years	SIDIAP, prospective database in primary care (Catalonia region)	2007–2017	Nonmelanoma cohort, 73.0 (14.1); MM cohort, 59.8 (19.3)	Nonmelanoma cohort, 431,643 (53.0); MM cohort, 49,181 (55.8)	HCTZ-users	Non-melanoma skin cancer (NS), MM
León-Muñoz et al. (2021) [56]: BIFAP cohort	Spain	Nested case-control	Nonmelanoma cohort, 341,693; MM cohort, 51271	Population-based: adults aged ≥ 18 years	BIFAP, prospective database in primary care (different Spanish regions)	2007–2017	Nonmelanoma cohort, 72.7 (14.1); MM cohort, 60.7 (18.5)	Nonmelanoma cohort, 176,418 (51.6); MM cohort, 28,633 (55.8)	HCTZ-users	BCC, SCC, MM
Rouette et al. (2021) [57]	UK	Retrospective cohort	41,026	Population-based: adults aged ≥ 18 years	CPRD, primary care and linked data	1 January 1988–31 March 2018	61.1 (14.9)	24,292 (59.2)	New users of HCTZ	BCC, SCC, MM
Schneider et al. (2021) [58]	UK	Retrospective cohort	546,417	Population-based: adults aged 18–85 years	CPRD, primary care and linked data	1 January 1998–31 December 2017	61.6 (13.4)	344,079 (63.0)	New users of thiazides and thiazide-like diuretics	BCC, SCC, MM

Abbreviations: BCC, basal cell carcinoma; BIFAP, Base de Datos para la Investigación Farmacoepidemiológica en Atención Primaria; CPRD, Clinical Practice Research Datalink; EHRs, electronic health records; FDA, Food and Drug Administration; HCTZ, hydrochlorothiazide; KPNC, Kaiser Permanente Northern California; MAST, malignant adnexal skin tumors; MCC, Merkel cell carcinoma; MM, malignant melanoma; NHIRD, National Health Insurance Research Database; NS, not specified; NR, not reported; RPGEH, Research Program in Genes and Environmental Health; SCC, squamous cell carcinoma; SIDIAP, Spain: Information System for Research in Primary Care; SEER, Surveillance, Epidemiology and End Results; THIN, The Health Improvement Network; UK, United Kingdom; US, United States.

**Table 2:** Summary of findings and strength of evidence.

Skin Cancer	No. of Included Studies (Sample Size)	OR (95% CI)	*p*-Value	E-Values for		95% Prediction Interval	Heterogeneity				Strength of Evidence (Evidence-Based Conclusion)
				Point Estimate	CI Limit		*Q* Statistic	*p*-Value	*I^2^* Index (95% CI)	τ^2^	
Primary Outcomes											
Malignant melanoma: All subtype	17 (*n* = 10,129,196)	1.10 (1.04–1.15)	<0.001	1.420	1.255	0.93–129	63.94	<0.001	73.4% (54.8–82.2)	0.005	Very low (very small harmful)
Malignant melanoma: Superficial spreading melanoma	3 (*n* = 221,624)	1.18 (1.05–1.33)	0.006	1.643	1.279	0.35–4.02	4.32	0.115	53.7% (0.0–85.3)	0.006	Very low (very small harmful)
Malignant melanoma: Nodular melanoma	3 (*n* = 36,631)	1.23 (1.08–1.40)	0.001	1.760	1.383	0.54–2.79	1.66	0.435	0.0% (0.0–72.9)	<0.001	Very low (very small harmful)
Malignant melanoma: Lentigo maligna melanoma	3 (*n* = 21,407)	1.33 (1.08–1.65)	0.008	2.001	1.365	0.18–10.09	3.17	0.205	36.9% (0.0–81.7)	0.013	Very low (very small harmful)
Non-melanoma skin cancer: BCC	14 (*n* = 19,780,476)	1.05 (1.02–1.09)	0.003	1.293	1.153	0.94–1.19	101.43	<0.001	87.2% (80.3–90.9)	0.003	Very low (very small harmful)
Non-melanoma skin cancer: SCC	16 (*n* = 16,387,862)	1.35 (1.22–1.48)	<0.001	2.026	1.743	0.93–1.95	511.45	<0.001	97.1% (96.5–97.5)	0.028	Very low (very small harmful)
Non-melanoma skin cancer: Unspecified	6 (*n* = 5,668,737)	1.08 (1.03–1.12)	0.001	1.362	1.210	0.94–1.23	35.38	<0.001	83.0% (62.5–90.1)	0.002	Very low (very small harmful)
Secondary Outcomes											
Lip cancer	5 (*n* = 161,491)	1.92 (1.52–2.42)	<0.001	3.249	2.409	0.97–3.81	8.25	0.083	51.5% (0.0–80.3)	0.032	Very low (small harmful)
MCC	2 (*n* = 4,763,703)	0.98 (0.57–1.65)	0.924	1.165	1.000	NA	0.12	0.732	0.0% (NA)	<0.001	Insufficient data (NA)
MAST	1 (*n* = 2752)	1.40 (0.86–2.29)	0.179	2.148	1.000	NA	NA	NA	NA	NA	Insufficient data (NA)
Oral cavity cancer	1 (*n* = 73,844)	0.90 (0.60–1.36)	0.614	1.462	1.000	NA	NA	NA	NA	NA	Insufficient data (NA)
Actinic keratosis	1 (*n* = 1029)	3.18 (1.93–5.25)	<0.001	5.813	3.270	NA	NA	NA	NA	NA	Insufficient data (NA)

Abbreviations: BCC, basal cell carcinoma; CI, confidence interval; MAST, malignant adnexal skin tumor; MCC, Merkel cell carcinoma; NA, not applicable; OR, odds ratio; SCC, squamous cell carcinoma.

## Data Availability

The data presented in this study are available in this article (and Supplementary Materials).

## Supplementary Materials

The following supporting information can be downloaded at: https://www.mdpi.com/article/10.3390/cancers14102566/s1, Table S1: Systematic Review Search Strategy; Table S2: The PICOTS Format: Study Inclusion/Exclusion Criteria; Table S3: Measurement and Definition of Skin Cancer Cases; Table S4: Methods of Included Studies in the Meta-Analysis; Table S5: Comorbidities and Skin Conditions of Study Participants Included in the Meta-Analysis; Table S6: Concomitant Medication Use of Included Studies; Table S7: Risk of Bias Assessment of Included Studies; Table S8: Subgroup Analysis; Table S9: Sensitivity Analysis: Restricted the Analysis to the Highest-Quality Study; Table S10: Sensitivity Analysis: Excluding Studies that Included Patients Who Underwent Organ Transplantation; Table S11: Sensitivity Analysis: Adding Unpublished Studies; Table S12: Sensitivity Analysis: Outcomes After Removing Individuals Studies; Table S13: Meta-Regression of Included Studies; Table S14: Meta-Analysis of Included Studies with Calibration for Publication Bias; Table S15: Quality of Evidence Synthesis and GRADE Evidence Profile of Outcomes; Figure S1: Study selection flowchart; Figure S2: Use of Thiazide Diuretics and the Risk of Lip Cancer; Figure S3: The Funnel Plot of Included Studies in the Meta-Analysis; File S1: MOOSE statement Checklist; File S2: PRISMA 2020 Statement Checklist; File S3: Pre-specified Protocol and Protocol Amendments; File S4: NOS for Assessing the Quality of Non-Randomized Studies; File S5: Modified Criteria of Evidence Certainty Assessment.; File S6: List of Excluded Articles.
