# Supplementary material for: Use of Thiazide Diuretics and Risk of All Types of Skin Cancers: An Updated Systematic Review and Meta-Analysis
Source: Cancers (Basel). 2022 May 23;14(10):2566. doi: 10.3390/cancers14102566 (PMC9140049; doi:10.3390/cancers14102566)
Supplement: Supplementary file 1 [file cancers-14-02566-s001.zip › cancers-1706242-supplementary.pdf]

Supplementary Information

Use of Thiazide Diuretics and Risk of All Types of Skin Cancers: An Updated Systematic Review and Meta-Analysis

Surapon Nochaiwong*; Mati Chuamanochan*; Chidchanok Ruengorn; Kajohnsak Noppakun; Ratanaporn Awiphan; Chabaphai Phosuya; Napatra Tovanabutra; Siri Chiewchanvit; Manish M. Sood; Brian Hutton; Kednapa Thavorn; Greg A. Knoll

***Correspondence:**
Surapon Nochaiwong, PharmD, Department of Pharmaceutical Care, Faculty of Pharmacy, Chiang Mai University, Chiang Mai 50200, Thailand, Phone: +66-899973365, Fax: 6653222741, Email: surapon.nochaiwong@cmu.ac.th

or

Mati Chuamanochan, MD, Division of Dermatology, Department of Internal Medicine, Faculty of Medicine, Chiang Mai University, Chiang Mai 50200, Thailand, Phone: +66-53936735-6, Fax: +66-53946234, Email: mati.c@cmu.ac.th

Supplementary Online Content

**Table S1**	Systematic Review Search Strategy	S3
**Table S2**	The PICOTS Format: Study Inclusion/Exclusion Criteria	S12
**Table S3**	Measurement and Definition of Skin Cancer Cases	S13
**Table S4**	Methods of Included Studies in the Meta-Analysis	S15
**Table S5**	Comorbidities and Skin Conditions of Study Participants Included in the Meta-Analysis	S22
**Table S6**	Concomitant Medication Use of Included Studies	S25
**Table S7**	Risk of Bias Assessment of Included Studies	S28
**Table S8**	Subgroup Analysis	S30
**Table S9**	Sensitivity Analysis: Restricted the Analysis to the Highest-Quality Study	S34
**Table S10**	Sensitivity Analysis: Excluding Studies that Included Patients Who Underwent Organ Transplantation	S35
**Table S11**	Sensitivity Analysis: Adding Unpublished Studies	S36
**Table S12**	Sensitivity Analysis: Outcomes After Removing Individuals Studies	S37
**Table S13**	Meta-Regression of Included Studies	S39
**Table S14**	Meta-Analysis of Included Studies with Calibration for Publication Bias	S41
**Table S15**	Quality of Evidence Synthesis and GRADE Evidence Profile of Outcomes	S42
**Figure S1**	Study selection flowchart	S47
**Figure S2**	Use of Thiazide Diuretics and the Risk of Lip Cancer	S48
**Figure S3**	The Funnel Plot of Included Studies in the Meta-Analysis	S49
**File S1**	MOOSE statement Checklist	S54
**File S2**	PRISMA 2020 Statement Checklist	S56
**File S3**	Pre-specified Protocol and Protocol Amendments	S58
**File S4**	NOS for Assessing the Quality of Non-Randomized Studies	S61
**File S5**	Modified Criteria of Evidence Certainty Assessment	S63
**File S6**	List of Excluded Articles	S64

Table S1 Systematic Review Search Strategy

**Ovid MEDLINE(R) ALL 1946 to May 7, 2021**		
**Search**	**Query**	**Items Found**
#1	exp Diuretics/	80589
#2	exp Thiazides/	15511
#3	exp Hydrochlorothiazide/	6878
#4	exp Sodium Potassium Chloride Symporter Inhibitors/	14177
#5	(Thiazide diuretic or Hydrochlorothiazide).mp.	9615
#6	(Diuretic or Thiazide* or (Na-K-Cl adj1 inhibit*) or (Sodium potassium chloride adj1 inhibit*)).ti,ab,kf.	25822
#7	(Bendroflumethiazide or Benzthiazide or Chlorthalidone or Chlorothiazide or Chlorphthalidolone or Chlortalidone or Cyclopenthiazide or Cyclothiazide or Dichlothiazide or Dihydrochlorothiazide or HCTZ or Hydrodiuril or Hydrochlorothiazide or Hydroflumethiazide or Hypothiazide or Indapamide or Methiclothiazide or Methyclothiazide or Metindamide or Metolazone or Oxodoline or Phthalamudine or Quinethazone or Thalitone or Xipamide).ti,ab,kf.	12081
#8	or/1-7	98849
#9	exp Skin Cancer/	128342
#10	exp Skin Neoplasms/	128342
#11	exp Melanoma/	98047
#12	exp Carcinoma, Basal Cell/	18243
#13	exp Nevus, Pigmented/	9948
#14	exp Keratosis, Actinic/	2146
#15	exp Precancerous Conditions/	51224
#16	((skin adj2 neoplasm*) or (skin adj2 cancer*) or (skin adj2 malignan*) or (skin adj2 metas*)).mp.	136584
#17	(skin adj3 (cancer* or neoplasm* or tumo$r or malignan*)).ti,ab,kf.	37180
#18	((skin$ or cutaneous$ or cutanea$ or dermis$ or corium or dermal$ or dermatolog$ or epiderm$ or subcutaneous$ or sub-cutaneous$ or hypodermis$ or (superficial$ adj2 fascia$) or subcutis$ or scalp$1 or acanthoma$) adj3 (cancer* or neoplasm* or tumo$r or malignan*)).ti,ab,kf.	54060
#19	(melanoma* or non-melanoma* or nonmelanoma* or NMSC or malignant melanoma or cutaneous melanoma or (squamous adj1 carcinoma) or (squamous$ adj3 (cell cancer$ or epithelioma$)) or (sebaceous cell adj2 carcinoma) or (basal cell adj2 carcinoma) or (pigmented and (lesion* or n?evi)) or mole* or ((actinic or solar or senile) adj keratos*) or keratinocyte cancer* or actinically damaged field or field-canceri$ed).ti,ab,kf.	2275053
#20	or/9-19	2427132
#21	8 and 20	3386
#22	(news or newspaper article or comment or editorial or interview or letter or review or systematic review or case report or case series or cross-sectional).pt.	5009870
#23	21 not 22	2872
#24	exp Clinical Trials as Topic/	356251
#25	(randomi$ed controlled trial* or controlled clinical trial*).mp.	126987
#26	(random allocation or double-blind method or single-blind method or clinical trial).mp.	893154

#27	((controlled clinical trial) or (randomi$ed controlled trial)).mp. or (clinical trial).pt	563742
#28	(control* adj2 trial*).tw,kw.	298539
#29	((clinical adj trial*) or (randomly allocated) or (allocated adj2 random*) or randomi$ed or RCT$1 placebo*).tw,kw.	427262
#30	((singl* or doubl* or trebl* or tribl*) adj (blind* or mask* or dumm*)).tw,kw.	178922
#31	or/24-29	1458024
#32	(nRCT or nRCTs or non-RCT?).tw,kw.	1054
#33	(control* adj2 stud$3).tw,kw.	245849
#34	control group/	1733
#35	(control* adj2 group$1).tw,kw.	535354
#36	exp comparative study/	1888825
#37	((comparative or comparison) adj stud$3).tw,kw.	115182
#38	exp cohort study/	2126118
#39	(cohort* adj2 stud$3).tw,kw.	259051
#40	exp case control study/	1166589
#41	((case-control* or case-based or case-comparison) adj stud$3).tw,kw.	133678
#42	or/32-41	4500775
#43	23 and 31	105
#44	23 and 42	348
#45	43 or 44	425
#46	exp Adolescent/ not (exp Adult/ and Adolescent/)	625583
#47	exp Child/ not (exp Adult/ and exp Child/)	1247576
#48	exp Infant/ not (exp Adult/ and exp Infant/)	851090
#49	or/46-48	1930046
#50	45 not 49	411
#51	limit 50 to human	209

Table S1 Systematic Review Search Strategy (Continued)

**Ovid Embase 1946 to May 7, 2021**		
**Search**	**Query**	**Items Found**
#1	exp Diuretics/	371565
#2	exp Thiazides/	50961
#3	exp Hydrochlorothiazide/	24605
#4	exp Sodium Potassium Chloride Symporter Inhibitors/	69189
#5	(Thiazide diuretic or Hydrochlorothiazide).mp.	42642
#6	(Diuretic or Thiazide* or (Na-K-Cl adj1 inhibit*) or (Sodium potassium chloride adj1 inhibit*)).ti,ab,kw.	34060
#7	(Bendroflumethiazide or Benzthiazide or Chlorthalidone or Chlorothiazide or Chlorphthalidolone or Chlortalidone or Cyclopenthiazide or Cyclothiazide or Dichlothiazide or Dihydrochlorothiazide or HCTZ or Hydrodiuril or Hydrochlorothiazide or Hydroflumethiazide or Hypothiazide or Indapamide or Methiclothiazide or Methyclothiazide or Metindamide or Metolazone or Oxodoline or Phthalamudine or Quinethazone or Thalitone or Xipamide).ti,ab,kw.	13612
#8	or/1-7	381891
#9	exp Skin Cancer/	104388
#10	exp Skin Neoplasms/	182814
#11	exp Melanoma/	163677
#12	exp Carcinoma, Basal Cell/	27444
#13	exp Nevus, Pigmented/	8172
#14	exp Keratosis, Actinic/	7367
#15	exp Precancerous Conditions/	20588
#16	((skin adj2 neoplasm*) or (skin adj2 cancer*) or (skin adj2 malignan*) or (skin adj2 metas*)).mp.	63256
#17	(skin adj3 (cancer* or neoplasm* or tumo$r or malignan*)).ti,ab,kw.	48704
#18	((skin$ or cutaneous$ or cutanea$ or dermis$ or corium or dermal$ or dermatolog$ or epiderm$ or subcutaneous$ or sub-cutaneous$ or hypodermis$ or (superficial$ adj2 fascia$) or subcutis$ or scalp$1 or acanthoma$) adj3 (cancer* or neoplasm* or tumo$r or malignan*)).ti,ab,kw.	71251
#19	(melanoma* or non-melanoma* or nonmelanoma* or NMSC or malignant melanoma or cutaneous melanoma or (squamous adj1 carcinoma) or (squamous$ adj3 (cell cancer$ or epithelioma$)) or (sebaceous cell adj2 carcinoma) or (basal cell adj2 carcinoma) or (pigmented and (lesion* or n?evi)) or mole* or ((actinic or solar or senile) adj keratos*) or keratinocyte cancer* or actinically damaged field or field-canceri$ed).ti,ab,kw.	2691695
#20	or/9-19	2880392
#21	8 and 20	21554
#22	(news or newspaper article or comment or editorial or interview or letter or review or systematic review or case report or case series or cross-sectional).pt.	4579713
#23	21 not 22	17556
#24	exp Clinical Trials as Topic/	358105
#25	(randomi$ed controlled trial* or controlled clinical trial*).mp.	507869
#26	(random allocation or double-blind method or single-blind method or clinical trial).mp.	1625224

#27	((controlled clinical trial) or (randomi$ed controlled trial)).mp. or (clinical trial).pt	495304
#28	(control* adj2 trial*).tw,kw.	404257
#29	((clinical adj trial*) or (randomly allocated) or (allocated adj2 random*) or randomi$ed or RCT$1 placebo*).tw,kw.	625320
#30	((singl* or doubl* or trebl* or tribl*) adj (blind* or mask* or dumm*)).tw,kw.	245457
#31	or/24-29	2102948
#32	(nRCT or nRCTs or non-RCT?).tw,kw.	1467
#33	(control* adj2 stud$3).tw,kw.	331839
#34	control group/	110068
#35	(control* adj2 group$1).tw,kw.	770006
#36	exp comparative study/	1400506
#37	((comparative or comparison) adj stud$3).tw,kw.	132168
#38	exp cohort study/	705392
#39	(cohort* adj2 stud$3).tw,kw.	392423
#40	exp case control study/	190826
#41	((case-control* or case-based or case-comparison) adj stud$3).tw,kw.	150276
#42	or/32-41	3182838
#43	23 and 31	911
#44	23 and 42	1201
#45	43 or 44	2003
#46	exp Adolescent/ not (exp Adult/ and Adolescent/)	576115
#47	exp Child/ not (exp Adult/ and exp Child/)	1870939
#48	exp Infant/ not (exp Adult/ and exp Infant/)	757181
#49	or/46-48	2102470
#50	45 not 49	1960
#51	limit 50 to human	1294

Table S1 Systematic Review Search Strategy (Continued)

**PubMed: From Inception to May 7, 2021**		
**Search**	**Query**	**Items Found**
#1	Diuretics[Pharmacological Action]	79912
#2	Diuretics OR Thiazides OR "Sodium Potassium Chloride Symporter Inhibitors" OR "Na K Cl cotransporter inhibitors" OR "Na K Cl symporter inhibitors" OR "Na-K-Cl cotransporter inhibitors" OR "Na-K-Cl symporter inhibitors" OR "Sodium potassium chloride cotransporter inhibitors" OR "Sodium potassium chloride symporter inhibitors"	108438
#3	Bendroflumethiazide OR Benzthiazide OR Chlorthalidone OR Chlorothiazide OR Chlorphthalidolone OR Chlortalidone OR Cyclopenthiazide OR Cyclothiazide OR Dichlothiazide OR Dihydrochlorothiazide OR HCTZ OR Hydrodiuril OR Hydrochlorothiazide OR Hydroflumethiazide OR Hypothiazide OR Indapamide OR Methiclothiazide OR Methyclothiazide OR Metindamide OR Metolazone OR Oxodoline OR Phthalamudine OR Quinethazone OR Thalitone OR Xipamide	15736
#4	#1 OR #2 OR #3	110424
#5	Skin Cancers[MeSH Terms]	128347
#6	(skin[Title/Abstract] OR cutaneous[Title/Abstract] OR dermatology[Title/Abstract] OR dermal[Title/Abstract] OR epidermal[Title/Abstract] OR subcutaneous[Title/Abstract] OR sub-cutaneous[Title/Abstract] OR hypodermis[Title/Abstract] OR superficial[Title/Abstract]) AND (cancer[Title/Abstract] OR neoplasm[Title/Abstract] OR tumor[Title/Abstract] OR tumour[Title/Abstract] OR malignancy[Title/Abstract] OR metastases[Title/Abstract])	186503
#7	melanoma[Title/Abstract] OR non-melanoma[Title/Abstract] OR nonmelanoma[Title/Abstract] OR NMSC[Title/Abstract] OR "malignant melanoma"[Title/Abstract] OR "cutaneous melanoma"[Title/Abstract] OR "squamous cell carcinoma"[Title/Abstract] OR "sebaceous cell carcinoma"[Title/Abstract] OR "basal cell carcinoma"[Title/Abstract] OR "pigmented nevus" OR moles[Title/Abstract] OR "actinic keratosis"[Title/Abstract] OR "solar keratosis"[Title/Abstract] OR "senile keratosis"[Title/Abstract] OR "keratinocyte cancer"[Title/Abstract] OR "actinically damaged field"[Title/Abstract] OR field-cancerized[Title/Abstract] OR "precancerous conditions"[Title/Abstract]	231456
#8	#5 OR #6 OR #7	434966
#9	#4 AND #8	324
#10	(((((((Case Reports[Publication Type]) OR Comment[Publication Type]) OR Editorial[Publication Type]) OR Guideline[Publication Type]) OR Letter[Publication Type]) OR News[Publication Type]) OR Newspaper Article[Publication Type]) OR Review[Publication Type]	6780467
#11	#9 NOT #10	208
#12	Filters: Humans	139

Table S1 Systematic Review Search Strategy (Continued)

**Cochrane Library: From Inception to May, 2021**		
**Search**	**Query**	**Items Found**
#1	MeSH descriptor: [Sodium Chloride Symporter Inhibitors] explode all trees	406
#2	Diuretics OR Thiazides OR "Sodium Potassium Chloride Symporter Inhibitors" OR "Na K Cl cotransporter inhibitors" OR "Na K Cl symporter inhibitors" OR "Na-K-Cl cotransporter inhibitors" OR "Na-K-Cl symporter inhibitors" OR "Sodium potassium chloride cotransporter inhibitors" OR "Sodium potassium chloride symporter inhibitors"	6031
#3	Bendroflumethiazide OR Benzthiazide OR Chlorthalidone OR Chlorothiazide OR Chlorphthalidolone OR Chlortalidone OR Cyclopenthiazide OR Cyclothiazide OR Dichlothiazide OR Dihydrochlorothiazide OR HCTZ OR Hydrodiuril OR Hydrochlorothiazide OR Hydroflumethiazide OR Hypothiazide OR Indapamide OR Methiclothiazide OR Methyclothiazide OR Metindamide OR Metolazone OR Oxodoline OR Phthalamudine OR Quinethazone OR Thalitone OR Xipamide	5951
#4	#1 OR #2 OR #3	10465
#5	MeSH descriptor: [Skin Neoplasms] explode all trees	1603
#6	(skin OR cutaneous OR dermatology OR dermal OR epidermal OR subcutaneous OR sub-cutaneous OR hypodermis OR superficial) AND (cancer OR neoplasm OR tumor OR tumour OR malignancy OR metastases)	21085
#7	melanoma OR non-melanoma OR nonmelanoma OR NMSC OR "malignant melanoma" OR "cutaneous melanoma" OR "squamous cell carcinoma" OR "sebaceous cell carcinoma" OR "basal cell carcinoma" OR "pigmented nevus" OR moles OR "actinic keratosis" OR "solar keratosis" OR "senile keratosis" OR "keratinocyte cancer" OR "actinically damaged field" OR field-cancerized OR "precancerous conditions"	13660
#8	#5 OR #6 OR #7	31116
#9	#4 AND #8	62

Table S1 Systematic Review Search Strategy (Continued)

**Web of Science: From Inception to May 7, 2021**		
**Search**	**Query**	**Items Found**
#1	TS=(Diuretic* OR Thiazide* OR "Sodium Potassium Chloride Symporter Inhibit*" OR "Na K Cl cotransporter inhibit*" OR "Na K Cl symporter inhibit*" OR "Na-K-Cl cotransporter inhibit*" OR "Na-K-Cl symporter inhibit*" OR "Sodium potassium chloride cotransporter inhibit*" OR "Sodium potassium chloride symporter inhibit*")	20885
#2	TS=(Bendroflumethiazide OR Benzthiazide OR Chlorthalidone OR Chlorothiazide OR Chlorphthalidolone OR Chlortalidone OR Cyclopenthiazide OR Cyclothiazide OR Dichlothiazide OR Dihydrochlorothiazide OR HCTZ OR Hydrodiuril OR Hydrochlorothiazide OR Hydroflumethiazide OR Hypothiazide OR Indapamide OR Methiclothiazide OR Methyclothiazide OR Metindamide OR Metolazone OR Oxodoline OR Phthalamudine OR Quinethazone OR Thalitone OR Xipamide)	6731
#3	#1 OR #2	25638
#4	TS=((skin OR cutaneous OR dermatology OR dermal OR epidermal OR subcutaneous OR sub-cutaneous OR hypodermis OR superficial) AND (cancer OR neoplasm OR tumor OR tumour OR malignancy OR metastases))	187898
#5	TS=(melanoma OR non-melanoma OR nonmelanoma OR NMSC OR "malignant melanoma" OR "cutaneous melanoma" OR "squamous cell carcinoma" OR "sebaceous cell carcinoma" OR "basal cell carcinoma" OR "pigmented nevus" OR moles OR "actinic keratosis" OR "solar keratosis" OR "senile keratosis" OR "keratinocyte cancer" OR "actinically damaged field" OR field-cancerized OR "precancerous conditions")	278412
#6	#4 OR #5	420955
#7	#3 AND #6	216
#8	Refined by: DOCUMENT TYPES: (ARTICLE OR MEETING ABSTRACT OR EARLY ACCESS OR PROCEEDINGS PAPER) Timespan: All years.	143

Table S1 Systematic Review Search Strategy (Continued)

**Scopus: From Inception to May 7, 2021**		
**Search**	**Query**	**Items Found**
#1	TITLE-ABS-KEY (diuretic* OR thiazide* OR "Sodium Potassium Chloride Symporter Inhibit*" OR "Na K Cl cotransporter inhibit*" OR "Na K Cl symporter inhibit*" OR "Na-K-Cl cotransporter inhibit*" OR "Na-K-Cl symporter inhibit*" OR "Sodium potassium chloride cotransporter inhibit*" OR "Sodium potassium chloride symporter inhibit*")	127983
#2	TITLE-ABS-KEY (bendroflumethiazide OR benzthiazide OR chlorthalidone OR chlorothiazide OR chlorphthalidolone OR chlortalidone OR cyclopenthiazide OR cyclothiazide OR dichlothiazide OR dihydrochlorothiazide OR hctz OR hydrodiuril OR hydrochlorothiazide OR hydroflumethiazide OR hypothiazide OR indapamide OR methiclothiazide OR methyclothiazide OR metindamide OR metolazone OR oxodoline OR phthalamudine OR quinethazone OR thalitone OR xipamide)	46808
#3	#1 OR #2	156490
#4	TITLE-ABS-KEY ((skin OR cutaneous OR dermatology OR dermal OR epidermal OR subcutaneous OR sub-cutaneous OR hypodermis OR superficial) AND (cancer OR neoplasm OR tumor OR tumour OR malignancy OR metastases))	516283
#5	TITLE-ABS-KEY (melanoma OR non-melanoma OR nonmelanoma OR nmsc OR "malignant melanoma" OR "cutaneous melanoma" OR "squamous cell carcinoma" OR "sebaceous cell carcinoma" OR "basal cell carcinoma" OR "pigmented nevus" OR moles OR "actinic keratosis" OR "solar keratosis" OR "senile keratosis" OR "keratinocyte cancer" OR "actinically damaged field" OR field-cancerized OR "precancerous conditions")	521973
#6	#4 OR #5	916367
#7	#3 AND #6	1587
#8	LIMIT-TO (PUBSTAGE , "final") OR LIMIT-TO (PUBSTAGE , "aip")) AND (LIMIT-TO (DOCTYPE , "ar")) AND (LIMIT-TO (SRCTYPE , "j"))	944

Table S1 Systematic Review Search Strategy (Continued)

**CINAHL: From Inception to May 7, 2021**		
**Search**	**Query**	**Items Found**
#1	AB diuretic* OR thiazide* OR "Sodium Potassium Chloride Symporter Inhibit*" OR "Na K Cl cotransporter inhibit*" OR "Na K Cl symporter inhibit*" OR "Na-K-Cl cotransporter inhibit*" OR "Na-K-Cl symporter inhibit*" OR "Sodium potassium chloride cotransporter inhibit*" OR "Sodium potassium chloride symporter inhibit*"	6149
#2	AB bendroflumethiazide OR benzthiazide OR chlorthalidone OR chlorothiazide OR chlorphthalidolone OR chlortalidone OR cyclopenthiazide OR cyclothiazide OR dichlothiazide OR dihydrochlorothiazide OR hctz OR hydrodiuril OR hydrochlorothiazide OR hydroflumethiazide OR hypothiazide OR indapamide OR methiclothiazide OR methyclothiazide OR metindamide OR metolazone OR oxodoline OR phthalamudine OR quinethazone OR thalitone OR xipamide	2395
#3	S1 OR S2	7864
#4	AB (skin OR cutaneous OR dermatology OR dermal OR epidermal OR subcutaneous OR sub-cutaneous OR hypodermis OR superficial) AND (cancer OR neoplasm OR tumor OR tumour OR malignancy OR metastases)	27799
#5	AB melanoma OR non-melanoma OR nonmelanoma OR nmsc OR "malignant melanoma" OR "cutaneous melanoma" OR "squamous cell carcinoma" OR "sebaceous cell carcinoma" OR "basal cell carcinoma" OR "pigmented nevus" OR moles OR "actinic keratosis" OR "solar keratosis" OR "senile keratosis" OR "keratinocyte cancer" OR "actinically damaged field" OR field-cancerized OR "precancerous conditions"	46054
#6	S4 OR S5	67760
#7	S3 AND S6	53

Table S2 The PICOTS Format: Study Inclusion/Exclusion Criteria

Study Elements	Criteria for Inclusion	Criteria for Exclusion
Populations	• Adult or adolescent participants aged 12 years or older which addressed at least one of the outcome of interest<br>• Other subgroups analysis were included if studies providing data to calculate the effect estimates of the outcome of interest	• In vitro or animal studies<br>• Studies including less than 50 participants were excluded owing to they lacked statistically significant power
Interventions	• Thiazide diuretics therapy for any indications	• Unclear definition of thiazide diuretics exposure
Comparators	• Non-thiazide-users or active comparators	• Studies without control groups
Outcomes	• Primary outcomes<br>❖ Malignant melanoma<br>❖ NMSC: BCC and SCC<br>• Secondary outcomes<br>❖ Lip cancer<br>❖ Merkel cell carcinoma<br>❖ Actinic keratosis<br>❖ Malignant adnexal skin tumor<br>❖ Oral cavity cancer	• Studies not providing data to calculate the effect estimates of the outcome of interest<br>• Inadequate control of confounders/unadjusted effect estimates
Timing	• An extensive search strategy from the inception of bibliographic databases forward to assure all published literature was identified	• No limit timing of start date
Setting	• Observational nonrandomized trials (cohort studies and case-control studies) with no language restriction	• N-of-one, cross-sectional, case series/case reports, pharmacokinetic/pharmacodynamics study, and RCTs design<br>• Reports not involving primary data including, narrative review, systematic review, meta-analysis, news items, consensus statement, guidelines, and opinion/editorials

Abbreviations: BCC, basal cell carcinoma; NA, not applicable; NMSC, non-melanoma skin cancer; PICOTS, populations, interventions, comparators, outcomes, timing, setting; RCTs, randomized controlled trial; SCC, squamous cell carcinoma.

Table S3 Measurement and Definition of Skin Cancer Cases

First Author, Year	Outcomes Measurement	Definition of Skin Cancer Cases
Westerdahl et al[1], 1996	Cancer registry data	First histopathological diagnosis of malignant melanoma
Jensen et al[2], 2008	EHRs linked with cancer registry data	First primary diagnosis of BCC, SCC or malignant melanoma: BCC (ICD-7 codes 1910-1919; ICD-O-1 codes 80903, 80913, 80923, 80933 and 81233), SCC (ICD-7 codes 1910-1919; ICD-O-1 codes 80513, 80703, 80713, 80743, 80763, 80943, and 80953), and malignant melanoma (ICD-7 codes 1900-1909)
Kaae et al[3], 2010	EHRs linked with cancer registry data	ICD-10 codes C43 and C44 (1995 to 2006); WHO histology codes: BCC (C44, histology codes 80903-80933), SCC (C44, histology codes 80513-80523, 80703-80763, 80943, or 85603), MCC (C44, histology code 82473), malignant melanoma (C43)
Ruiter et al[4], 2010	Prospective registries data	Diagnosis of BCC using ICD-10, including histo- and cytopathology (two research physicians independently assessed the first date and diagnosis)
de Vries et al[5], 2012	Medical record: hospital-based data	Histological confirmation and a maximum of 3 months since diagnosis was required
Friedman et al[6], 2012	EHRs linked with cancer registry data	Based on SEER program
Traianou et al[7], 2012	Medical record: hospital-based data	Histological confirmation and a maximum of 3 months since diagnosis was required
Robinson et al[8], 2013	Medical record along with the New Hampshire Skin Cancer Study	Histological confirmation of SCC
Schmidt et al[9], 2015	EHRs linked with cancer registry data	Based on ICD-O-3 and ICD-10
Nardone et al[10], 2017	EHRs link with pathology data from the hospital	Primary diagnosis of BCC, SCC or malignant melanoma using ICD-9: BCC (codes 171.01-173.91), SCC (codes 173.02-179.92), malignant melanoma (172.0-172.9)
Pottegård et al[11], 2017	EHRs linked with cancer registry data	Biopsy-verified first diagnosis of SCC of the lip
Pedersen et al[12], 2018	EHRs linked with cancer registry data	First diagnosis of BCC or SCC of the skin
Pottegård et al[13], 2018	EHRs linked with cancer registry data	Histologically verified malignant melanoma cases
Shaw et al[14], 2018	Along with the VAKCC trial	Histopathological confirmed of SCC
Su et al[15], 2018	EHRs: KPNC	Biopsy-proven SCC
Pedersen et al[16], 2019	EHRs linked with cancer registry data	Histologically verified primary diagnosis of MCC or malignant adnexal skin tumor
Pottegård et al[17], 2019	EHRs: NHIRD	First ever diagnosis using ICD-9 code: NMSC of the lip (code 1730), non-lip NMSC (codes 1731-1739), malignant melanoma (codes 1720-1729)
Daniels et al[18], 2020	EHRs linked with cancer registry data	Histologically confirmed diagnosis of lip cancer or malignant melanoma
Lee et al[19], 2020	EHRs linked with 3 hospital-based centers in Korean	KCD-8 codes, which are similar to ICD-10 codes: NMSC (C44, D04), malignant melanoma (C43, D03)
Letellier et al[20], 2020	EHRs linked with prospective DIVAT cohort of patients with transplants	Histologically confirmed keratinocyte cancers (NMSC)

Abbreviations: BCC, basal cell carcinoma; DIVAT, Données Informatisées et VAlidées en Transplantation; EHRs, electronic health records; ICD, International Classification of Diseases; ICD-O, International Classification of Diseases for Oncology; KCD, Korean Classification of Disease; KPNC, Kaiser Permanente Northern California; MCC, Merkel cell carcinoma; NHIRD, National Health Insurance Research Database; NMSC, non-melanoma skin cancer; SCC, squamous cell carcinoma; SEER, Surveillance, Epidemiology, and End Results; VAKCC, Veterans Affairs Keratinocyte Carcinoma Chemoprevention; WHO, World Health Organization.

Table S3 Measurement and Definition of Skin Cancer Cases (Continued)

First Author, Year	Outcomes Measurement	Definition of Skin Cancer Cases
Morales et al[21], 2020	EHRs: THIN	Using the Read Code clinical classification system, a hierarchical classification system, linked to the ICD
Park et al[22], 2020	EHRs: HIRA	Diagnosis is confirmed by pathologic examination: KCD-8 codes, which are similar to ICD-10 codes: NMSC (C44, D04); malignant melanoma (C43, D03)
Yeon et al[23], 2020	EHRs: KNHI	ICD-10 code C44 for NMSC
Adalsteinsson et al[24], 2021	EHRs linked with cancer registry data	All cases of skin cancer diagnosed with histologic verification
de Haan-Du et al[25], 2021	EHRs linked with cancer registry data	Using the Dutch Pathology Network based on histologic, cytologic, and autopsy reports submitted by pathology departments
Drucker et al[26], 2021	EHRs linked with administrative health data from Ontario, Canada	BCC and SCC: using a validated OHIP claims-based algorithm (sensitivity 83%-85%, specificity 93%-99%); malignant melanoma: using the Ontario Cancer Registry, captures 94% of melanomas diagnosed in Ontario (ICD-O codes C440-C449)
Eworuke et al[27], 2021	EHRs: US FDA Sentinel System with a PPV of 98.7% for outcome algorithm	BCC (ICD-9 codes 173.x1 or ICD-10 codes C44.x1x), SCC (ICD-9 codes 173.x2 or ICD-10 codes C44.x2x)
Habel et al[28], 2021	EHRs: KPNC	Histologic diagnosis based on cancer registry, including pathology reports
Kim et al[29], 2021	EHRs: KNHI	Diagnosis of NMSC was based on the ICD-10 (codes C44, D04)
León-Muñoz et al[30], SIDIAP cohort 2021	EHRs: SIDIAP—routine visits in primary care	Malignant melanoma (ICPC-2, ICD-9, ICD-10); NMSC (ICPC-2, ICD-9, ICD-10); unspecified skin cancer (ICPC-2, ICD-9), ICD-10)
León-Muñoz et al[30], BIFAP cohort 2021	EHRs: BIFAP—routine visits in primary care	Malignant melanoma (ICPC-2, ICD-9, ICD-10); NMSC (ICPC-2, ICD-9, ICD-10); BCC (ICPC-2, ICD-9, ICD-10), SCC (ICPC-2, ICD-9, ICD-10); unspecified skin cancer (ICPC-2, ICD-9), ICD-10)
Rouette et al[31], 2021	EHRs using CPRD Gold linked with National Cancer Registry	Read codes: BCC (PPV=93%), SCC (PPV=83%), and malignant melanoma (PPV=85%)
Schneider et al[32], 2021	EHRs using CPRD Gold and HES APC data	Frist time diagnosis of skin cancers using Read codes: BCC or SCC (PPV=85%), malignant melanoma (PPV=93%)

Abbreviations: BCC, basal cell carcinoma; BIFAP, Base de Datos para la Investigación Farmacoepidemiológica en Atención Primaria; CPRD, Clinical Practice Research Datalink; EHRs, electronic health records; HES APC, Hospital Episode Statistics Admitted Patient Care; HIRA, Health Insurance Review and Assessment Service; ICD, International Classification of Diseases; ICD-O, International Classification of Diseases for Oncology; ICPC-2, International Classification of Primary Care; KCD, Korean Classification of Disease; KNHI, Korean National Health Insurance; KPNC, Kaiser Permanente Northern California; NMSC, non-melanoma skin cancer; NR, not reported; OHIP, Ontario Health Insurance Plan; PPV, positive predictive value; SCC, squamous cell carcinoma; SIDIAP, Spain: Information System for Research in Primary Care; THIN, The Health Improvement Network; US FDA, United States Food and Drug Administration.

Table S4 Methods of Included Studies in the Meta-Analysis

Author, Year	Population/Case Patients	Definition of Exposure	Definition of Non-exposure	Statistical Methods for Data Analysis	Factors Controlled for in Analysis
Westerdahl et al[1], 1996	Participants in the South Swedish Health Care Region	Self-reported: used prescribed thiazide diuretics >1 month continuously	Control: up to 2 controls using a random sampling and matched by sex, age (within a year), and parish	Multivariable conditional logistic regression models	Matching variables (age, sex, and parish), history of sunburns and host factors (hair color, number of raised naevi)
Jensen et al[2], 2008	Adult Danish residents in North Jutland Country	Thiazide/Thiazide-like-users (bendroflumethiazide, indapamide, HCTZ): any prescriptions filled, >1 years and >5 years before the index date	Control: up to 4 controls matched by exact age, sex, and area of residence based on risk set sampling	Multivariable conditional logistic regression models	Matching variables (age, sex, and area of residence), prior hospitalization for selected chronic diseases and use of glucocorticoids
Kaae et al[3], 2010	All Danish residents	Bendroflumethiazide users: filled at least 1 prescription	Non-photosensitizing medication users	Multivariable Poisson regression models	Age, period, sex, and education
Ruiter et al[4], 2010	Rotterdam study cohort: mainly Caucasians	Thiazides user (chlorthalidone and thiazides in combination with other drugs): prescriptions during the study period	Non-thiazides-users (NS)	Multivariable Cox proportional hazards regression models	Age at baseline and gender
de Vries et al[5], 2012	Hospital-based adult European populations in Finland, Germany, Greece, Italy, Malta, Poland, Scotland, and Spain	Self-reported: used prescribed thiazide diuretics (bendroflumethiazide) ≥3 months of regular (daily)	Control: frequency-match for age (in 5-year age bands) and sex in each country (visiting the hospital clinics for any condition unrelated to skin cancer); 2 controls per malignant melanoma and 1 control per non-melanoma skin cancer	Multivariable unconditional logistic regression models	Age, sex, Fitzpatrick skin type (or phototype), and country
Friedman et al[6], 2012	Adult non-Hispanic whites in the San Francisco Bay area and central valley of California	HCTZ-users, defined as 3 or more filled prescriptions	Control: up to 50 risk-set controls and matched on age, sex, and year of entry into the cohort	Multivariable conditional logistic regression models	Matching variables (age, sex, and year of entry into the cohort) and smoking habits
Traianou et al[7], 2012	Hospital-based adult European populations in Finland, Germany, Greece, Italy, Malta, Poland, Scotland, and Spain	Self-reported: used prescribed thiazide diuretics ≥3 months of regular (daily)	Control: frequency matched for age and sex (cases to controls ratio was 1:2)	Multivariable unconditional logistic regression models	Age, sex, and country
Robinson et al[8], 2013	Adult residents of New Hampshire, speak English	Self-reported: used prescribed thiazides (HCTZ, including combination medication) ≥6 months of regular (at least 4 times/week)	Control: frequency matched for age and sex	Multivariable logistic regression models	Matching variables (age and sex), number of painful sunburns, study phase, lifetime hours of warm months sun exposure, skin response to first hour of sun in summer, and tanning lamp use

Abbreviations: HCTZ, hydrochlorothiazide; NS, not specified.

Table S4 Methods of Included Studies in the Meta-Analysis (Continued)

Author, Year	Population/Case Patients	Definition of Exposure	Definition of Non-exposure	Statistical Methods for Data Analysis	Factors Controlled for in Analysis
Schmidt et al[9], 2015	Adult Danish residents in northern Denmark	Thiazides users (bendroflumethiazide, hydroflumethiazide, HCTZ, chlorothiazide): had redeemed >2 prescription before the index date	Control: up to 10 controls using a risk-set sampling and matched by birth year, sex, and country of residence	Multivariable conditional logistic regression models	Matching variables (birth year, sex, and country of residence), CCI, obesity, medication use (glucocorticoids, aspirin, NSAIDs, statins)
Nardone et al[10], 2017	All adult persons receiving treatment through Northwestern University healthcare affiliates	Thiazide-users, who had one or more written orders for thiazide diuretics	Non-thiazides-users (no documented any antihypertensive drug): randomly selected 3:1 ratio and matched by time to follow-up (± 3 months) and by age (± 5 years)	Multivariable logistic regression models	Age, gender, race, and CCI
Pottegård et al[11], 2017	Adult Danish residents	HCTZ-users, filled at least 1 prescription for a HCTZ-containing drug prior to the index date	Control: up to 100 controls using a risk-set sampling strategy and matched by age, gender, and calendar time	Multivariable conditional logistic regression models	Matching variables (age, gender, and calendar time), history of heavy alcohol consumption, diabetes, COPD, history of non-melanoma skin cancer, CCI, highest achieved education, and medication use (photosensitizing drugs, aspirin, NSAIDs, statins)
Pedersen et al[12], 2018	Adult Danish residents	HCTZ-users, filled at least 1 prescription for a HCTZ-containing drug prior to the index date	Control: up to 20 controls using a risk-set sampling strategy and matched by age, sex and calendar time	Multivariable conditional logistic regression models	Matching variables (age, gender, and calendar time), history of heavy alcohol consumption, diabetes, chronic renal insufficiency, COPD, CCI, highest achieved education, and medication use (photosensitizing drugs, aspirin, NSAIDs, statins)
Pottegård et al[13], 2018	Adult Danish residents	HCTZ-users, filled at least 1 prescription for a HCTZ-containing drug prior to the index date	Control: matched 1:10 using a risk-set sampling strategy by age, sex and calendar time	Multivariable conditional logistic regression models	Matching variables (age, gender, and calendar time), history of non-melanoma skin cancer, comorbidity (diabetes, COPD, alcohol abuse-associated disorders, chronic renal insufficiency), CCI, highest achieved education, and medication use (photosensitizing drugs, aspirin, NSAIDs, statins or oral steroids)

Abbreviations: CCI, Charlson Comorbidity Index; COPD, Chronic obstructive pulmonary disease; HCTZ, hydrochlorothiazide; NSAIDs, non-steroidal anti-inflammatory drugs.

Table S4 Methods of Included Studies in the Meta-Analysis (Continued)

Author, Year	Population/Case Patients	Definition of Exposure	Definition of Non-exposure	Statistical Methods for Data Analysis	Factors Controlled for in Analysis
Shaw et al[14], 2018	Veterans participants at high risk for skin cancer (history of at least 2 keratinocyte carcinomas in the past 5 years)	HCTZ-users based on Veterans Health Administration pharmacy records (NS)	Non-HCTZ-users (NS)	Cox proportional hazards regression models	NS
Su et al[15], 2018	Adult non-Hispanic White participants aged 18 years and older with hypertension in a closed healthcare system in northern California	Thiazide-users, defined as two or more filled prescriptions during the study period	Non-thiazide-users (nonusers of any antihypertensive drugs)	Multivariable cox proportional hazards regression models	Age, sex, smoking, comorbidities, history of squamous cell carcinoma and actinic keratosis, survey year, healthcare utilization, surveillance measure, length of the health plan membership and prior history of photosensitizing antihypertensive drug use
Pedersen et al[16], 2019	Adult Danish residents	HCTZ-users, at least one filled prescription of a HCTZ-containing drug before the index date	Control: up to 20 controls via risk-set sampling and matched based on age, sex and calendar time	Multivariable conditional logistic regression models	Matching variables (age, sex, and calendar time), comorbidities (diabetes, COPD, history of heavy alcohol consumption, and chronic renal insufficiency), CCI, highest achieved education, and medication use (photosensitizing drugs, aspirin, NSAIDs, statins or oral steroids)
Pottegård et al[17], 2019	Adult Taiwanese residents	HCTZ-users, at least one filled prescription of a HCTZ-containing drug before the index date	Control: up to 10 controls among all Taiwanese residents were selected using a risk-set sampling and matched based on sex, age (birth year and month), and calendar time	Multivariable conditional logistic regression models	Matching variables (age, sex, and calendar time), comorbidities (diabetes and COPD), CCI, and medication use (photosensitizing drugs, aspirin, NSAIDs, and statins)
Daniels et al[18], 2020	Adult patients aged 65 years and older within a population of veterans residing in New South Wales	HCTZ-users, filled as a single agent or in combination with other medicines	Control: up to 20 controls via risk-set sampling and matched based on age at the time Gold Card benefits began and sex	Multivariable conditional logistic regression models	Matching variables (age and sex), CCI, ambient ultraviolet radiation exposure, and medication use (photosensitizing drugs, aspirin, NSAIDs, and statins)

Abbreviations: CCI, Charlson Comorbidity Index; COPD, Chronic obstructive pulmonary disease; HCTZ, hydrochlorothiazide; NS, not specified; NSAIDs, non-steroidal anti-inflammatory drugs.

Table S4 Methods of Included Studies in the Meta-Analysis (Continued)

Author, Year	Population/Case Patients	Definition of Exposure	Definition of Non-exposure	Statistical Methods for Data Analysis	Factors Controlled for in Analysis
Lee et al[19], 2020	Adult patients aged 20-80 years	HCTZ-users (NS): HCTZ-only use (no other prescription drugs than HCTZ); Combination use (use of combination of HCTZ and other HTN diuretics); Ever use (both HCTZ-only and combination use)	Non-HCTZ-users (use of other antihypertensive agents)	Propensity score matching; Multivariable Cox proportional hazards regression models	Age, sex, CCI, comorbidities (diabetes and COPD), medication use (aspirin, NSAIDs, and statins)
Letellier et al[20], 2020	Adult patients transplanted with a graft that functioned for at least 3 months (kidney, pancreases, or combined kidney-pancreases). Patients received mTOR inhibitors or azathioprine were excluded.	HCTZ-users, prescribed in the post-transplant periods (at least 1 month)	Non-HCTZ-users	Multivariable time-varying Cox proportional hazards regression models	Age, sex, re-transplantation, type of transplantation, type of donor, HLA-A -B -DR mismatch ≥4, induction therapy, maintenance treatment at transplantation, use of steroids at transplantation, initial nephropathy, cold ischemia time, and for time-varying covariates (rejection, maintenance treatment during follow-up, and other malignancies
Morales et al[21], 2020	Population-based, using the longitudinal electronic medical records from general practices across the UK	HCTZ-users, filled 1 or more prescription before the index date	Control: randomly selected using incidence density sampling up to 20 controls matched on sex, exact year of birth and calendar year of cohort entry (up to 100 controls for the analysis with lip cancer)	Multivariable conditional logistic regression models	Matching variables (age and sex), body mass index, smoking status, CCI, comorbidities (history of alcohol abuse, DM, and COPD) and medication use (photosensitizing drugs, aspirin, NSAIDs, and statins)
Park et al[22], 2020	Population-based, adult patients aged 18 years and older with a first diagnosis of primary hypertension	HCTZ-users, filled at least 3 prescription for HCTZ	non-HCTZ-users (treated with other antihypertensive agents and never prescribed for HCTZ during the follow-up period)	Multivariable Cox proportional hazards regression models	Age, sex, CCI, and medication use (photosensitizing drugs, aspirin, statins, and oral corticosteroids)
Yeon et al[23], 2020	Patients with hypertensive disorder older than 30 years	HCTZ-users, from entry date to 2 years prior to the index date	Control: randomly matched 4 population controls by sex, age, and entry date	Multivariable conditional logistic regression models	Matching variables (age, sex, and entry date), other factors (NS)

Abbreviations: CCI, Charlson Comorbidity Index; COPD, Chronic obstructive pulmonary disease; HCTZ, hydrochlorothiazide; mTOR, Mammalian target of rapamycin; NS, not specified; NSAIDs, non-steroidal anti-inflammatory drugs; UK, United Kingdom.

Table S4 Methods of Included Studies in the Meta-Analysis (Continued)

**Author, Year**	**Population/Case Patients**	**Definition of Exposure**	**Definition of Non-exposure**	**Statistical Methods for Data Analysis**	**Factors Controlled for in Analysis**
Adalsteinsson et al[24], 2021	Population-based: the entire Icelandic population	Had filled 1 or more HCTZ prescriptions at least 2 years before the index date (date of keratinocyte carcinoma diagnosis)	Control: up to 10 population control individuals were randomly selected from the National Registry of Iceland and matched based on year of birth and sex	Multivariable conditional logistic regression models	Matching variables (age and sex), and photosensitizing drugs
de Haan-Du et al[25], 2021	Adult type 2 diabetes patients	HCTZ users (including its combination) at baseline; thiazide-like diuretics (chlorthalidone and indapamide)	Non-HCTZ users	Multivariable Cox proportional hazards regression models	Age, sex, smoking status, body mass index, systolic blood pressure, serum creatinine, and medications (angiotensin-converting enzyme inhibitors, angiotensin-receptor blockers, calcium channel blockers, beta-blockers, other diuretics, sulfonylureas, metformin, lipid lowering drugs, and NSAIDs)
Drucker et al[26], 2021	Population-based: patients aged 65 years and older	New users of thiazides: chlorthalidone, HCTZ, indapamide, metolazone	Unexposed to antihypertensive (age- and sex-matched)	Multivariable Cox proportional hazards regression models	Age, sex, rurality, income according to postal code, year of index date, number of physician visits, CCI, hypertension, and time-varying covariates (medication use: immunosuppressive drugs, photosensitizing drugs, cumulative dosage/duration of other antihypertensive classes, and ever use of each antihypertensive class)
Eworuke et al[27], 2021	Patient with no diagnosis of any cancer type, no use of any chemotherapeutic agent in any formulation, and no radiation therapy	New users of any HCTZ-containing products	New user of angiotensin-converting enzyme inhibitors monotherapy or non-HCTZ angiotensin-converting enzyme inhibitors combination products	Propensity score matching (nearest neighbor approach, caliper=0.05); Multivariable Cox proportional hazards regression models	Age, sex, actinic keratosis, arsenic exposure, diabetes, human papillomavirus, chronic pulmonary disease, connective tissue disease, cardiovascular disease, moderate/severe renal disease, transplant, HIV, immune disorders, severe skin disease, white blood cell disease, mole removal, naevi, xeroderma pigmentosum, ultraviolet radiation exposure, alcohol use/abuse, comorbidity score, health service utilization intensity, medications (drugs with chemoprotective effects, glucocorticoids, photosensitizing medications)

Abbreviations: CCI, Charlson Comorbidity Index; HCTZ, hydrochlorothiazide; HIV, human immunodeficiency virus; NSAIDs, non-steroidal anti-inflammatory drugs.

Table S4 Methods of Included Studies in the Meta-Analysis (Continued)

Author, Year	Population/Case Patients	Definition of Exposure	Definition of Non-exposure	Statistical Methods for Data Analysis	Factors Controlled for in Analysis
Habel et al[28], 2021	Adult non-Hispanic White participants	HCTZ-users (ascertained from cohort entry to two year before index date and was based on prescriptions filled at KPNC pharmacies	Control: up to 50 cancer-free control, matched for birth year (exact year), sex, and year of joining KPNC (exact year)	Multivariable conditional logistic regression models	Matching variables (age, sex, and calendar time), highest education achieved and socioeconomic level based on the United States Census block of resident, and number of ambulatory visits, including dermatology visits, internal medicine visits, and urgent care visits for the period from start of follow-up to 1 year prior to the index date
Kim et al[29], 2021	Adult participants randomly selected from 97.1% of people in the country enrolled into the national health insurance	HCTZ-users, had been taking for >6 months with a cumulative dose of ≥2,500 mg	Non-HCTZ-users, randomly selected and matched for age, index date, sex and income level	Multivariable Cox proportional hazards regression models	Age, sex, income level, CCI, comorbidities (hypertension, diabetes, dyslipidemia, thyroid disorders), medication use (photosensitizing drugs)
León-Muñoz et al[30], SIDIAP cohort 2021	Population-based: patients aged 18 years and older	HCTZ-users (alone or in combination with other active drug: filled at least one prescription)	Control: up to 10 controls using a risk-set sampling strategy matched to cases by sex and age ± 1 year	Multivariable conditional logistic regression models	Age, sex, time up to index date, smoking, comorbid conditions (diabetes, COPD, chronic kidney disease, myocardial infection, heart failure, peripheral vascular disease, cerebrovascular accident, dementia/Alzheimer disease, connective tissue disease, gastric ulcer, hemiplegia, liver disease), and medication use (photosensitizing drugs, aspirin, NSAIDs, glucocorticoids, and statins)
León-Muñoz et al[30], BIFAP cohort 2021	Population-based: patients aged 18 years and older	HCTZ-users (alone or in combination with other active drug: filled at least one prescription)	Control: up to 10 controls using a risk-set sampling strategy matched to cases by sex and age ± 1 year	Multivariable conditional logistic regression models	Age, sex, time up to index date, smoking, comorbid conditions (diabetes, COPD, chronic kidney disease, myocardial infection, heart failure, peripheral vascular disease, cerebrovascular accident, dementia/Alzheimer disease, connective tissue disease, gastric ulcer, hemiplegia, liver disease), and medication use (photosensitizing drugs, aspirin, NSAIDs, glucocorticoids, and statins)

Abbreviations: BIFAP, Base de Datos para la Investigación Farmacoepidemiológica en Atención Primaria; CCI, Charlson Comorbidity Index; COPD, Chronic obstructive pulmonary disease; HCTZ, hydrochlorothiazide; KPNC, Kaiser Permanente Northern California; NSAIDs, non-steroidal anti-inflammatory drugs; SIDIAP, Spain: Information System for Research in Primary Care.

Table S4 Methods of Included Studies in the Meta-Analysis (Continued)

Author, Year	Population/Case Patients	Definition of Exposure	Definition of Non-exposure	Statistical Methods for Data Analysis	Factors Controlled for in Analysis
Rouette et al[31], 2021	Population-based: patients aged 18 years and older	New users of HCTZ	New user of other thiazide diuretics (bendroflumethiazide, chlorothiazide, trichlormethiazide, methyclothiazide, polythiazide, quinethazone, hydroflumethiazide, benzthiazide, cyclopenthiazide, mefruside, indapamide, chlorthalidone, clopamide, xipamide, and metolazone)	Propensity score matching (nearest neighbor approach, caliper=0.01); Multivariable Cox proportional hazards regression models	Age, sex, body mass index, smoking status, number of physician visits in the year before cohort entry, skin cancer risk factors (presence of naevi and precancerous skin lesions), comorbid conditions (alcohol-related disorder, chronic heart failure, myocardial infarction, stroke, peripheral vascular disease, previous cancers--other than skin cancer, rheumatoid arthritis, inflammatory bowel disorder, psoriasis, lupus, vasculitis, and previous organ transplantation), and medication use (NSAIDs, statins, antidiabetic drugs, antihypertensive drugs, antiparkinsonian drugs, and immunosuppressive and immunomodulatory drugs)
Schneider et al[32], 2021	Population-based: patients aged 18-85 years	New users of thiazides and thiazide-like diuretics	Active comparator: calcium channel blockers (or renin-angiotensin-aldosterone system inhibitors)	Propensity score weighted; Multivariable negative binomial and Poisson regression models	Age, sex, year of cohort entry, region, smoking status, alcohol consumption, body mass index, number of days of history within the CPRD prior to cohort entry date, number of general practitioner visits in the year prior to cohort entry date, number of different prescribed agents in the year prior to cohort entry date, CCI, comorbidities (diabetes, COPD, myocardial infarction, ischemic heart disease, chronic heart failure, and hypertension), medication use (photosensitizing drugs, aspirin, NSAIDs, statins, beta-blockers, and renin-angiotensin-aldosterone system inhibitors)

Abbreviations: CCI, Charlson Comorbidity Index; CPRD, Clinical Practice Research Datalink; COPD, Chronic obstructive pulmonary disease; HCTZ, hydrochlorothiazide; NSAIDs, non-steroidal anti-inflammatory drugs.

Table S5 Comorbidities and Skin Conditions of Study Participants Included in the Meta-Analysis

Author, Year	Smoking Status, No. (%)	Hypertension, No. (%)	Diabetes, No. (%)	Chronic Pulmonary Disease, No. (%)	Naevi, No. (%)	Precancer skin, No. (%)	Ultraviolet Exposure, No. (%)	Solid Organ Transplant, No. (%)
Westerdahl et al[1], 1996	Current, 222 (24.4)	NR	NR	NR	NR	NR	NR	NR
Jensen et al[2], 2008	NR	NR	NR	NR	NR	NR	NR	NR
Kaae et al[3], 2010	NR	NR	NR	NR	NR	NR	NR	NR
Ruiter et al[4], 2010	Current, 2286 (21.5); former, 4644 (43.6); never, 3707 (34.9)	NR	NR	NR	NR	NR	High tendency to sunburn, 3216 (30.1); outdoor work (>4 h daily for >25 years), 1187 (11.1)	NR
de Vries et al[5], 2012	Current, 519 (17.8); former, 848 (29.1); never, 1551 (53.1)	NR	NR	NR	2196 (75.2)	NR	Having (had) outdoor occupation, 1236 (66.1)	NR
Friedman et al[6], 2012	Current, 6100 (25.8); former, 6971 (29.5); never, 3902 (16.5); unknown, 6643 (28.1)	NR	NR	NR	NR	NR	NR	0 (0.0)
Traianou et al[7], 2012	NR	NR	NR	NR	NR	NR	NR	NR
Robinson et al[8], 2013	NR	NR	NR	NR	NR	NR	NR	NR
Schmidt et al[9], 2015	NR	25467 (10.0)	NR	11329 (4.4)	NR	NR	NR	0 (0.0)
Nardone et al[10], 2017	NR	NR	NR	NR	NR	NR	NR	0 (0.0)
Pottegård et al[11], 2017	NR	NR	5182 (8.1)	3722 (5.8)	NR	NR	NR	0 (0.0)
Pedersen et al[12], 2018	NR	NR	BCC cohort, 101272 (6.7); SCC cohort, 15350 (8.5)	BCC cohort, 69863 (4.6); SCC cohort, 11589 (6.4)	NR	NR	NR	0 (0.0)
Pottegård et al[13], 2018	NR	NR	NR	NR	NR	NR	NR	0 (0.0)

Abbreviations: BCC, basal cell carcinoma; NR, not reported; SCC, squamous cell carcinoma.

Table S5 Comorbidities and Skin Conditions of Study Participants Included in the Meta-Analysis (Continued)

Author, Year	Smoking Status, No. (%)	Hypertension, No. (%)	Diabetes, No. (%)	Chronic Pulmonary Disease, No. (%)	Naevi, No. (%)	Precancer skin, No. (%)	Ultraviolet Exposure, No. (%)	Solid Organ Transplant, No. (%)
Shaw et al[14], 2018	NR	NR	NR	0 (0.0)	NR	NR	NR	0 (0.0)
Su et al[15], 2018	Current, 2219 (7.8%); past, 13247 (46.7%); never, 12217 (43.1%); unknown, 674 (2.4%)	18244 (100.0)	NR	NR	NR	Actinic keratosis, 9972 (35.2)	NR	0 (0.0)
Pedersen et al[16], 2019	NR	NR	MCC cohort, 206 (10.5); MAST cohort, 258 (9.4)	MCC cohort, 154 (7.9); MAST cohort, 190 (6.9)	NR	NR	NR	0 (0.0)
Pottegård et al[17], 2019	NR	NR	51741 (16.2)	10688 (3.3)	NR	NR	NR	0 (0.0)
Daniels et al[18], 2020	NR	NR	NR	NR	NR	NR	Lip cohort: lowest, NR; middle, 664 (72.9); highest, NR; MM cohort: lowest, 1859 (14.2); middle, 9418 (71.9); highest, 1828 (13.9)	0 (0.0)
Lee et al[19], 2020	NR	NR	100562 (33.6)	23998 (8.0)	NR	NR	NR	0 (0.0)
Letellier et al[20], 2020	NR	NR	NR	NR	NR	NR	NR	2496 (100.0)
Morales et al[21], 2020	Current: SCC cohort, 14951 (9.4); BCC cohort, 215346 (11.5); melanoma cohort, 32963 (14.0); lip cohort, 10013 (14.1); oral cavity cohort, 11485 (15.6)	NR	NR	NR	NR	NR	NR	0 (0.0)
Park et al[22], 2020	NR	3565952 (100.0)	NR	NR	NR	NR	NR	0 (0.0)
Yeon et al[23], 2020	NR	NR	NR	NR	NR	NR	NR	NR

Abbreviations: BCC, basal cell carcinoma; MAST, malignant adnexal skin tumors; MCC, Merkel cell carcinoma; MM, malignant melanoma; NR, not reported; SCC, squamous cell carcinoma.

Table S5 Comorbidities and Skin Conditions of Study Participants Included in the Meta-Analysis (Continued)

Author, Year	Smoking Status, No. (%)	Hypertension, No. (%)	Diabetes, No. (%)	Chronic Pulmonary Disease, No. (%)	Naevi, No. (%)	Precancer skin, No. (%)	Ultraviolet Exposure, No. (%)	Solid Organ Transplant, No. (%)
Adalsteinsson et al[24], 2021	NR	NR	NR	NR	NR	NR	NR	NR
de Haan-Du et al[25], 2021	No, 47497 (67.4); ever, 14813 (21.0); unknown, 8184 (11.6)	NR	70494 (100.0)	NR	NR	NR	NR	NR
Drucker et al[26], 2021	NR	101807 (38.8)	NR	NR	NR	0 (0.0)	NR	0 (0.0)
Eworuke et al[27], 2021	NR	NR	2532702 (24.3)	1313252 (12.6)	72958 (0.7)	Actinic keratosis, 302256 (2.9)	Low, 10422 (0.1); moderate, 4320185 (41.4); high, 4179480 (40.1); very high, 1751004 (16.8); extreme, 10422 (0.1); unknown, 145916 (1.4)	26057 (0.3)
Habel et al[28], 2021	NR	NR	NR	NR	NR	NR	NR	NR
Kim et al[29], 2021	NR	69447 (55.8)	23664 (19.0)	NR	NR	298 (0.2)	NR	118 (0.1)
León-Muñoz et al[30], SIDIAP cohort 2021	Non-melanoma cohort, 96164 (11.8); MM cohort, 14291 (16.2)	NR	Non-melanoma cohort, 159453 (19.6); MM cohort, 11050 (12.5)	NR	NR	NR	NR	0 (0.0)
León-Muñoz et al[30], BIFAP cohort 2021	Non-melanoma cohort, 58300 (17.1%); MM cohort, 9965 (19.4%)	NR	Non-melanoma cohort, 55876 (16.4); MM cohort, 5914 (11.5)	NR	NR	NR	NR	0 (0.0)
Rouette et al[31], 2021	Current, 10574 (25.8); past, 5579 (13.6); never, 18591 (45.3); unknown, 6282 (15.3)	NR	NR	NR	1249 (3.0)	2773 (6.8)	NR	36 (0.1)
Schneider et al[32], 2021	Current, 99420 (18.2); past, 135063 (24.7); never, 260469 (47.7); unknown, 51465 (9.4)	459836 (84.2)	22814 (4.2)	22294 (4.1)	NR	NR	NR	0 (0.0)

Abbreviations: BCC, basal cell carcinoma; MAST, malignant adnexal skin tumors; MCC, Merkel cell carcinoma; MM, malignant melanoma; NR, not reported; SCC, squamous cell carcinoma.

Table S6 Concomitant Medication Use of Included Studies

Author, Year	Immuno-suppressant, No. (%)	RAS Inhibitors, No. (%)	Beta-blockers, No. (%)	Calcium Channel Blockers, No. (%)	Aspirin, No. (%)	NSAIDs, n (%)	Statins, n (%)	Photosensitivity Agents, No. (%)
Westerdahl et al[1], 1996	NR	NR	95 (10.5)	NR	NR	NR	NR	NR
Jensen et al[2], 2008	NR	NR	NA	NR	NR	NR	NR	NR
Kaae et al[3], 2010	NR	NR	NA	NR	NR	NR	NR	NR
Ruiter et al[4], 2010	NR	NR	NR	NR	NR	NR	NR	NR
de Vries et al[5], 2012	0 (0.0)	NR	NR	NR	NR	411 (14.1)	NR	NR
Friedman et al[6], 2012	NR	3391 (14.4)	2765 (11.7)	1002 (4.2)	NR	NR	NR	NR
Traianou et al[7], 2012	0 (0.0)	NR	NR	NR	NR	NR	NR	NR
Robinson et al[8], 2013	NR	NR	NR	NR	NR	NR	NR	NR
Schmidt et al[9], 2015	NR	40612 (15.9)	40338 (15.8)	33334 (13.1)	44246 (17.4)	81107 (31.8)	34955 (13.7)	NR
Nardone et al[10], 2017	NR	5609 (9.2)	NR	NR	NR	NR	NR	NR
Pottegård et al[11], 2017	0 (0.0)	NR	NR	NR	18066 (28.4)	32950 (51.7)	13024 (20.4)	28128 (44.2)
Pedersen et al[12], 2018	0 (0.0)	NR	NR	NR	BCC cohort, 298917 (19.9); SCC cohort, 57292 (31.6)	BCC cohort, 763444 (50.8); SCC cohort, 94179 (52.0)	BCC cohort, 238108 (15.8); SCC cohort, 34192 (18.9)	NR
Pottegård et al[13], 2018	0 (0.0)	NR	NR	NR	NR	NR	NR	NR
Shaw et al[14], 2018	NR	NR	NR	NR	NR	NR	NR	NR
Su et al[15], 2018	NR	ACEIs, 11658 (41.1); ARBs, 5251 (18.5)	15792 (55.7)	9383 (33.1)	NR	NR	NR	NR

Abbreviations: ACEIs, angiotensin-converting enzyme inhibitors; ARBs, angiotensin receptor blockers; BCC, basal cell carcinoma; NR, not reported; NSAIDs, non-steroidal anti-inflammatory drugs; RAS, renin-angiotensin system; SCC, squamous cell carcinoma.

Table S6 Concomitant Medication Use of Included Studies (Continued)

Author, Year	Immuno-suppressant, No. (%)	RAS Inhibitors, No. (%)	Beta-blockers, No. (%)	Calcium Channel Blockers, No. (%)	Aspirin, No. (%)	NSAIDs, n (%)	Statins, n (%)	Photosensitivity Agents, No. (%)
Pedersen et al[16], 2019	0 (0.0)	NR	NR	NR	MCC cohort, 687 (34.7); MAST cohort, 729 (26.5)	MCC cohort, 1130 (57.8); MAST cohort, 1480 (53.8)	MCC cohort, 496 (25.4); MAST cohort, 600 (21.8)	NR
Pottegård et al[17], 2019	NR	NR	NR	NR	65803 (20.6)	213271 (66.7)	49752 (15.6)	22079 (6.9)
Daniels et al[18], 2020	0 (0.0)	Lip cohort, 589 (64.6); MM cohort, 10012 (76.4)	NR	Lip cohort, 212 (23.3); MM cohort, 4825 (36.8)	Lip cohort, NA; MM cohort, 452 (3.4)	Lip cohort, 374 (41.1); MM cohort, 5067 (38.7)	Lip cohort, 495 (54.3); MM cohort, 7063 (53.9)	Lip cohort, 165 (18.1); MM cohort, 2416 (18.4)
Lee et al[19], 2020	0 (0.0)	NR	NR	NR	131330 (43.9)	106502 (35.6)	114675 (38.3)	NR
Letellier et al[20], 2020	2496 (100.0)	NR	NR	NR	NR	NR	NR	NR
Morales et al[21], 2020	0 (0.0)	NR	NR	NR	SCC cohort, 42036 (26.5); BCC cohort, 348516 (18.6); MM cohort, 27811 (11.8); lip cohort, 10971 (15.4); oral cavity cohort, 10163 (13.8)	SCC cohort, 62449 (39.3); BCC cohort, 651384 (34.8); MM cohort, 73139 (31.1); lip cohort, 22009 (30.9); oral cavity cohort, 24218 (32.8)	SCC cohort, 46259 (29.1); BCC cohort, 359782 (19.2); MM cohort, 32715 (13.9); lip cohort, 11754 (16.5); oral cavity cohort, 12841 (17.4)	NR
Park et al[22], 2020	0 (0.0)	NR	NR	NR	NR	NR	NR	NR
Yeon et al[23], 2020	NR	NR	NR	NR	NR	NR	NR	NR
Adalsteinsson et al[24], 2021	0 (0.0)	NR	NR	NR	NR	NR	NR	NR
de Haan-Du et al[25], 2021	NR	25338 (35.9)	19547 (27.7)	10307 (14.6)	NR	3977 (5.6)	NR	NR
Drucker et al[26], 2021	0 (0.0)	NR	NR	NR	NR	NR	NR	0 (0.0)

Abbreviations: BCC, basal cell carcinoma; MAST, malignant adnexal skin tumors; MCC, Merkel cell carcinoma; MM, malignant melanoma; NR, not reported; NSAIDs, non-steroidal anti-inflammatory drugs; RAS, renin-angiotensin system; SCC, squamous cell carcinoma.

Table S6 Concomitant Medication Use of Included Studies (Continued)

Author, Year	Immuno-suppressant, No. (%)	RAS Inhibitors, No. (%)	Beta-blockers, No. (%)	Calcium Channel Blockers, No. (%)	Aspirin, No. (%)	NSAIDs, n (%)	Statins, n (%)	Photosensitivity Agents, No. (%)
Eworuke et al[27], 2021	2892283 (27.7)	5211321 (50.0)	NR	NR	NR	NR	NR	1407056 (13.5)
Habel et al[28], 2021	NR	NR	NR	NR	NR	NR	NR	NR
Kim et al[29], 2020	NR	NR	NR	NR	NR	63409 (50.9)	NR	13155 (10.6)
León-Muñoz et al[30], SIDIAP cohort 2021	0 (0.0)	Non-melanoma cohort, 408621 (50.2); MM cohort, 26784 (30.4)	NR	Non-melanoma cohort, 109595 (13.5); MM cohort, 6470 (7.3)	Non-melanoma cohort, 171941 (21.1); MM cohort, 10383 (11.8)	Non-melanoma cohort, 430043 (52.8); MM cohort, 38862 (44.1)	Non-melanoma cohort, 276612 (34.0); MM cohort, 19350 (22.0)	Non-melanoma cohort, 91032 (11.2); MM cohort, 6929 (7.9)
León-Muñoz et al[30], BIFAP cohort 2021	0 (0.0)	Non-melanoma cohort, 155181 (45.4); MM cohort, 15535 (30.3)	NR	Non-melanoma cohort, 49035 (14.4); MM cohort, 4707 (9.2)	Non-melanoma cohort, 58451 (17.1); MM cohort, 5491 (10.7)	Non-melanoma cohort, 158367 (46.3); MM cohort, 23286 (45.4)	Non-melanoma cohort, 96970 (28.4); MM cohort, 10544 (20.6)	Non-melanoma cohort, 38738 (11.3); MM cohort, 5117 (10.0)
Rouette et al[31], 2021	1154 (2.8)	21563 (52.6)	10843 (26.4)	9761 (23.8)	NR	22792 (55.6)	8195 (20.0)	NR
Schneider et al[32], 2021	0 (0.0)	111048 (20.3)	99234 (18.2)	275263 (50.0)	69174 (12.7%)	129203 (23.6)	69666 (12.7)	48891 (8.9)

Abbreviations: BCC, basal cell carcinoma; MM, malignant melanoma; NR, not reported; NSAIDs, non-steroidal anti-inflammatory drugs; RAS, renin-angiotensin system; SCC, squamous cell carcinoma.

Table S7 Risk of Bias Assessment of Included Studies by the NOS

First Author, Year	Cohort Studies									
	Selection				Comparability		Outcomes			Total NOS
	Representa-tiveness	Non-Exposed: Selection	Exposure: Ascertainment	Outcomes Not Present at Entry	Controls for: age and Fitzpatrick skin type/sun exposure	Control for: additional Factors†	Assessment	Follow-up Long Enough	Adequacy of follow-up	
Kaae et al[3], 2010	*	*	*	*	…	…	*	*	*	7
Ruiter et al[4], 2010	*	*	*	*	…	…	*	*	*	7
Nardone et al[10], 2017	*	*	*	*	…	*	*	*	*	8
Su et al[15], 2018	*	*	*	*	…	*	*	*	*	8
Lee et al[19], 2020	*	*	*	*	…	*	*	*	*	8
Letellier et al[20], 2020	…	*	*	*	…	*	*	*	*	7
Park et al[22], 2020	*	*	*	*	…	*	*	*	*	8
de Haan-Du et al[25], 2021	*	*	*	*	…	*	*	*	*	8
Drucker et al[26], 2021	*	*	*	*	…	*	*	*	*	8
Eworuke et al[27], 2021	*	*	*	*	*	*	*	*	*	9
Kim et al[29], 2020	*	*	*	*	…	*	*	*	*	8
Rouette et al[31], 2021	*	*	*	*	…	*	*	*	*	8
Schneider et al[32], 2021	*	*	*	*	…	*	*	*	*	8

†Study control for 3 of additional factors: sex, race/ethnic, skin tanning, smoking, alcohol consumption, precancerous skin conditions, comorbidities, photosensitizing drugs, medications with potential antineoplastic properties (e.g., aspirin, NSAIDs, statins), other antihypertensive agents, human papillomavirus; immunosuppression/immunosuppressive drugs, environmental hazards/occupational risks (e.g., exposure of arsenic, polycyclic aromatic hydrocarbons, nitrosamine, alkylating agents), and genetic predisposing factors (e.g., xeroderma pigmentosum).

Abbreviations: NOS, Newcastle-Ottawa Scale; NSAIDs, non-steroidal anti-inflammatory drugs.

Table S7 Risk of Bias Assessment of Included Studies (Continued)

First Author, Year	Case-Control Studies									
	Selection				Comparability		Exposure			Total NOS
	Cases: Definition	Cases: Representa-tiveness	Controls: Selection	Controls: Definitions	Controls for: age and Fitzpatrick skin type/sun exposure	Control for: additional Factors†	Ascertainment	Same Method	Non-Response Rate	
Westerdahl et al[1], 1996	*	*	*	*	*	…	…	*	…	6
Jensen et al[2], 2008	*	*	*	*	…	*	*	*	*	8
de Vries et al[5], 2012	*	*	*	*	*	…	…	*	…	6
Friedman et al[6], 2012	*	*	*	*	…	…	*	*	*	7
Traianou et al[7], 2012	*	*	*	*	…	…	…	*	…	5
Robinson et al[8], 2013	*	*	*	*	*	…	…	*	*	7
Schmidt et al[9], 2015	*	*	*	*	…	*	*	*	*	8
Pottegård et al[11], 2017	*	*	*	*	…	*	*	*	*	8
Pedersen et al[12], 2018	*	*	*	*	…	*	*	*	*	8
Pottegård et al[13], 2018	*	*	*	*	…	*	*	*	*	8
Pedersen et al[16], 2019	*	*	*	*	…	*	*	*	*	8
Pottegård et al[17], 2019	*	*	*	*	…	*	*	*	*	8
Daniels et al[18], 2020	*	*	*	*	*	*	*	*	*	9
Morales et al[21], 2020	*	*	*	*	…	*	*	*	*	8
Adalsteinsson et al[24], 2021	*	*	*	*	…	…	*	*	*	7
Habel et al[28], 2021	*	*	*	*	…	…	*	*	*	7
León-Muñoz et al[30], SIDIAP cohort 2021	*	*	*	*	…	*	*	*	*	8
León-Muñoz et al[30], BIFAP cohort 2021	*	*	*	*	…	*	*	*	*	8

†Study control for 3 of additional factors: sex, race/ethnic, skin tanning, smoking, alcohol consumption, precancerous skin conditions, comorbidities, photosensitizing drugs, medications with potential antineoplastic properties (e.g., aspirin, NSAIDs, statins), other antihypertensive agents, human papillomavirus; immunosuppression/immunosuppressive drugs, environmental hazards/occupational risks (e.g., exposure of arsenic, polycyclic aromatic hydrocarbons, nitrosamine, alkylating agents), and genetic predisposing factors (e.g., xeroderma pigmentosum).

Abbreviations: NOS, Newcastle-Ottawa Scale; NSAIDs, non-steroidal anti-inflammatory drugs.

Table S8 Subgroup Analysis

Subgroup Comparison	No. of Studies (Ref)	No. of Participants	Odds Ratio (95% CI)	*P* Value	Heterogeneity			
					Q Statistic	*P* Value	I^2 Index (95% CI)	τ^2
**Malignant melanoma**								
**Individual thiazide diuretics**								
Hydrochlorothiazide	11 (2, 13, 17, 18, 19, 21, 22, 28, 30, 31, 32)	5001137	1.07 (1.00 – 1.15)	0.065	54.35	<0.001	79.8% (63.1 – 87.0)	0.010
Bendroflumethiazide	4 (2, 3, 5, 32)	5315126	1.08 (0.97 – 1.21)	0.155	4.79	0.188	37.4% (0.0 – 78.5)	0.005
Mixed cases	17 (1, 2, 3, 5, 9, 10, 13, 17, 18, 19, 21, 22, 26, 28, 30, 31, 32)	10129196	1.10 (1.04 – 1.15)	<0.001	63.94	<0.001	73.4% (54.8 – 82.2)	0.005
**Sample size**								
<10,000	3 (1, 2, 5)	7868	1.22 (1.05 – 1.40)	0.008	0.17	0.919	0.0% (0.0 – 72.9)	<0.001
≥10,000	14 (3, 9, 10, 13, 17, 18, 19, 21, 22, 26, 28, 30, 31, 32)	10121328	1.09 (1.03 – 1.15)	0.002	61.3	<0.001	77.2% (60.3 – 84.9)	0.006
**Study design**								
Case-control	10 (1, 2, 5, 9, 13, 17, 18, 21, 28, 30)	978532	1.12 (1.06 – 1.19)	<0.001	30.17	0.001	66.8% (25.0 – 80.9)	0.005
Cohort	7 (3, 10, 19, 22, 26, 31, 32)	9150664	1.07 (0.92 – 1.23)	0.384	23.44	0.001	74.4% (30.4 – 86.3)	0.021
**Study location**								
European	9 (1, 2, 3, 9, 13, 21, 30, 31, 32)	5981640	1.13 (1.08 – 1.19)	<0.001	28.11	0.001	68.0% (24.4 – 81.9)	0.003
North America	3 (10, 26, 28)	597196	1.22 (0.98 – 1.52)	0.078	4.14	0.126	51.7% (0.0 – 84.9)	0.020
International/Other	5 (5, 17, 18, 19, 22)	3550360	0.99 (0.85 – 1.15)	0.918	12.53	0.014	68.1% (0.0 – 85.6)	0.017

Abbreviations: CI, confidence interval; NA, not applicable.

Table S8 Subgroup Analysis (Continued)

Subgroup Comparison	No. of Studies (Ref)	No. of Participants	Odds Ratio (95% CI)	*P* Value	Heterogeneity			
					Q Statistic	*P* Value	I^2 Index (95% CI)	τ^2
**Basal cell carcinoma**								
**Individual thiazide diuretics**								
Hydrochlorothiazide	9 (2, 12, 20, 21, 24, 27, 30, 31, 32)	14755829	1.05 (1.01 – 1.10)	0.020	54.22	<0.001	85.2% (72.7 – 90.6)	0.002
Bendroflumethiazide	4 (2, 3, 5, 32)	5340507	1.05 (1.01 – 1.09)	0.014	5.50	0.139	45.4% (0.0 – 80.6)	0.001
Mixed cases	14 (2, 3, 4, 5, 9, 10, 12, 20, 21, 24, 27, 30, 31, 32)	19780476	1.05 (1.02 – 1.09)	0.003	101.43	<0.001	87.2% (80.3 – 90.9)	0.003
**Sample size**								
<10,000	3 (2, 5, 20)	34837	1.01 (0.80 – 1.29)	0.913	4.38	0.112	54.3% (0.0 – 85.5)	0.026
≥10,000	11 (3, 4, 9, 10, 12, 21, 24, 27, 30, 31, 32)	19745639	1.06 (1.02 – 1.10)	0.002	96.42	<0.001	89.6% (83.8 – 92.7)	0.003
**Study design**								
Case-control	7 (2, 5, 9, 12, 21, 24, 30)	3934790	1.07 (1.04 – 1.09)	<0.001	7.98	0.239	24.8% (0.0 – 68.0)	<0.001
Cohort	7 (3, 4, 10, 20, 27, 31, 32)	15845686	1.04 (0.98 – 1.10)	0.196	70.84	<0.001	91.5% (85.5 – 94.3)	0.004
**Study location**								
European	11 (2, 3, 4, 9, 12, 20, 21, 24, 30, 31, 32)	9294649	1.05 (1.03 – 1.08)	<0.001	22.59	0.012	55.7% (0.0 – 75.9)	0.001
North America	2 (10, 27)	10483306	1.43 (0.68 – 2.99)	0.348	28.38	<0.001	96.5% (NA)	0.276
International/Other	1 (5)	2521	1.27 (0.92 – 1.75)	0.145	NA	NA	NA	NA

Abbreviations: CI, confidence interval; NA, not applicable.

Table S8 Subgroup Analysis (Continued)

Subgroup Comparison	No. of Studies (Ref)	No. of Participants	Odds Ratio (95% CI)	*P* Value	Heterogeneity			
					Q Statistic	*P* Value	I^2 Index (95% CI)	τ^2
**Squamous cell carcinoma**								
**Individual thiazide diuretics**								
Hydrochlorothiazide	11 (2, 8, 12, 20, 21, 24, 25, 27, 30, 31, 32)	11510080	1.48 (1.18 – 1.84)	0.001	1052.71	<0.001	99.1% (0.0 – 99.9)	0.125
Bendroflumethiazide	4 (2, 3, 5, 32)	5315770	1.09 (0.97 – 1.22)	0.157	6.42	0.093	53.3% (0.0 – 82.7)	0.007
Mixed cases	16 (2, 3, 5, 8, 9, 10, 12, 15, 20, 21, 24, 25, 27, 30, 31, 32)	16387862	1.35 (1.22 – 1.48)	<0.001	511.45	<0.001	97.1% (96.5 – 97.5)	0.028
**Sample size**								
<10,000	4 (2, 5, 8, 20)	13605	1.46 (1.15 – 1.86)	0.002	5.78	0.123	48.1% (0.0 – 81.3)	0.028
≥10,000	12 (3, 9, 10, 12, 15, 21, 24, 25, 27, 30, 31, 32)	16374257	1.32 (1.19 – 1.47)	<0.001	502.60	<0.001	97.8% (97.4 – 98.1)	0.028
**Study design**								
Case-control	8 (2, 5, 8, 9, 12, 21, 24, 30)	454017	1.31 (1.11 – 1.54)	0.001	100.78	<0.001	93.1% (89.2 – 95.1)	0.045
Cohort	8 (3, 10, 15, 20, 25, 27, 31, 32)	15933845	1.36 (1.21 – 1.54)	<0.001	283.39	<0.001	97.5% (96.8 – 98.0)	0.020
**Study location**								
European	11 (2, 3, 9, 12, 20, 21, 24, 25, 30, 31, 32)	5870735	1.33 (1.18 – 1.49)	<0.001	189.90	<0.001	94.7% (92.8 – 95.9)	0.031
North America	4 (8, 10, 15, 27)	10515168	1.37 (1.11 – 1.70)	0.004	51.41	<0.001	94.2% (88.8 – 96.3)	0.031
International/Other	1 (5)	1959	1.66 (1.16 – 2.37)	0.005	NA	NA	NA	NA

Abbreviations: CI, confidence interval; NA, not applicable.

Table S8 Subgroup Analysis (Continued)

**Subgroup Comparison**	**No. of Studies (Ref)**	**No. of Participants**	**Odds Ratio (95% CI)**	***P* Value**	**Heterogeneity**			
					***Q* Statistic**	***P* Value**	**I^2 Index (95% CI)**	**τ^2**
**Non-melanoma skin cancer (unspecified)**								
**Individual thiazide diuretics**								
Hydrochlorothiazide	5 (17, 19, 22, 29, 30)	5406162	1.08 (1.02 – 1.13)	0.005	34.43	<0.001	85.5% (67.2 – 91.6)	0.002
Bendroflumethiazide	NA	NA	NA	NA	NA	NA	NA	NA
Mixed cases	6 (17, 19, 22, 26, 29, 30)	5668737	1.08 (1.03 – 1.12)	0.001	35.38	<0.001	(83.0% (62.5 – 90.1)	0.002
**Sample size**								
<10,000	NA	NA	NA	NA	NA	NA	NA	NA
≥10,000	6 (17, 19, 22, 26, 29, 30)	5668737	1.08 (1.03 – 1.12)	0.001	35.38	<0.001	83.0% (62.5 – 90.1)	0.002
**Study design**								
Case-control	2 (17, 30)	1416526	1.12 (1.09 – 1.14)	<0.001	3.15	0.207	36.5% (0.0 – 81.6)	<0.001
Cohort	4 (19, 22, 26, 29)	4252211	1.03 (0.93 – 1.15)	0.521	13.52	0.004	77.8% (0.0 – 89.9)	0.007
**Study location**								
European	1 (30)	1155793	1.12 (1.09 – 1.15)	<0.001	2.11	0.146	52.6% (NA)	<0.001
North America	1 (26)	262575	1.08 (1.03 – 1.14)	0.003	NA	NA	NA	NA
International/Other	4 (17, 19, 22, 29)	4250369	1.05 (0.93 – 1.17)	0.442	19.95	<0.001	85.0% (51.0 – 92.4)	0.009

Abbreviations: CI, confidence interval; NA, not applicable.

Table S9 Sensitivity Analysis: Restricted the Analysis to the Highest-Quality Study (NOS ≥8 Points)

Skin Cancer	No. of Studies (Ref)	No. of Participants	Odds Ratio (95% CI)	*P* Value	Heterogeneity			
					Q Statistic	*P* Value	I^2 Index (95% CI)	τ^2
**Primary outcomes**								
Malignant melanoma (all subtype)	13 (2, 9, 10, 13, 17, 18, 19, 21, 22, 26, 30, 31, 32)	5090672	1.10 (1.04 – 1.15)	0.006	60.81	<0.001	78.6% (62.6 – 85.9)	0.007
Non-melanoma skin cancer: basal cell carcinoma	9 (2, 9, 10, 12, 21, 27, 30, 31, 32)	14951026	1.06 (1.02 – 1.11)	0.004	91.24	<0.001	91.2% (86.1 – 93.9)	0.003
Non-melanoma skin cancer: squamous cell carcinoma	11 (2, 9, 10, 12, 15, 21, 25, 27, 30, 31, 32)	11593636	1.36 (1.22 – 1.52)	<0.001	499.95	<0.001	98.0% (97.6 – 98.3)	0.028
Non-melanoma skin cancer: Unspecified	6 (17, 19, 22, 26, 29, 30)	5668737	1.08 (1.03 – 1.12)	0.001	35.38	<0.001	83.0% (62.5 – 90.1)	0.002
**Secondary outcomes**								
Lip cancer	4 (11, 17, 18, 21)	137875	1.81 (1.28 – 2.55)	0.001	7.27	0.064	58.7% (0.0 – 84.2)	0.068
Merkel cell carcinoma	1 (16)	1954	1.00 (0.58 – 1.73)	1.000	NA	NA	NA	NA
Malignant adnexal skin tumors	1 (16)	2752	1.40 (0.86 – 2.29)	0.179	NA	NA	NA	NA
Oral cavity cancer	1 (21)	73844	0.90 (0.60 – 1.36)	0.614	NA	NA	NA	NA
Actinic keratosis	NA	NA	NA	NA	NA	NA	NA	NA

Abbreviations: CI, confidence interval; NA, not applicable; NOS, Newcastle-Ottawa scale.

Table S10 Sensitivity Analysis: Excluding Studies that Included Patients Who Underwent Organ Transplantation

Skin Cancer	No. of Studies (Ref)	No. of Participants	Odds Ratio (95% CI)	*P* Value	Heterogeneity			
					Q Statistic	*P* Value	I^2 Index (95% CI)	τ^2
**Primary outcomes**								
Malignant melanoma (all subtype)	16 (1, 2, 3, 5, 9, 10, 13, 17, 18, 19, 21, 22, 26, 28, 30, 32)	10088170	1.10 (1.04 – 1.15)	<0.001	59.80	<0.001	73.2% (53.5 – 82.3)	0.005
Non-melanoma skin cancer: basal cell carcinoma	11 (2, 3, 4, 5, 9, 10, 12, 21, 24, 30, 32)	9314312	1.06 (1.03 – 1.10)	<0.001	43.10	<0.001	76.8% (54.1 – 85.7)	0.002
Non-melanoma skin cancer: squamous cell carcinoma	13 (2, 3, 5, 8, 9, 10, 12, 15, 21, 24, 25, 30, 32)	5921698	1.36 (1.22 – 1.51)	<0.001	219.77	<0.001	94.5% (92.7 – 95.7)	0.029
Non-melanoma skin cancer: Unspecified	5 (17, 19, 22, 26, 30)	5544251	1.07 (1.03 – 1.12)	0.034	32.92	<0.001	84.8% (64.9 – 91.2)	0.002
**Secondary outcomes**								
Lip cancer	5 (6, 11, 17, 18, 21)	161491	1.92 (1.52 – 2.42)	<0.001	8.25	0.083	51.5% (0.0 – 80.3)	0.032
Merkel cell carcinoma	1 (16)	1954	1.00 (0.58 – 1.73)	1.000	NA	NA	NA	NA
Malignant adnexal skin tumors	1 (16)	2752	1.40 (0.86 – 2.29)	0.179	NA	NA	NA	NA
Oral cavity cancer	1 (21)	73844	0.90 (0.60 – 1.36)	0.614	NA	NA	NA	NA
Actinic keratosis	1 (7)	1029	3.18 (1.93 – 5.25)	<0.001	NA	NA	NA	NA

Abbreviations: CI, confidence interval; NA, not applicable; NOS, Newcastle-Ottawa scale.

Table S11 Sensitivity Analysis: Adding Unpublished Studies

Skin Cancer	No. of Studies (Ref)	No. of Participants	Odds Ratio (95% CI)	*P* Value	Heterogeneity			
					Q Statistic	*P* Value	I^2 Index (95% CI)	τ^2
**Primary outcomes**								
Malignant melanoma (all subtype)	NA	NA	NA	NA	NA	NA	NA	NA
Non-melanoma skin cancer: basal cell carcinoma	NA	NA	NA	NA	NA	NA	NA	NA
Non-melanoma skin cancer: squamous cell carcinoma	17 (2, 3, 5, 8, 9, 10, 12, 14, 15, 20, 21, 24, 25, 27, 30, 31, 32)	16388794	1.35 (1.23 – 1.48)	<0.001	513.12	<0.001	96.9% (96.3 – 97.4)	0.028
Non-melanoma skin cancer: Unspecified	6 (17, 19, 22, 23, 26, 29, 30)	5721562	1.07 (1.02 – 1.11)	0.002	47.51	<0.001	85.3% (71.3 – 90.8)	0.002
**Secondary outcomes**								
Lip cancer	NA	NA	NA	NA	NA	NA	NA	NA
Merkel cell carcinoma	NA	NA	NA	NA	NA	NA	NA	NA
Malignant adnexal skin tumors	NA	NA	NA	NA	NA	NA	NA	NA
Oral cavity cancer	NA	NA	NA	NA	NA	NA	NA	NA
Actinic keratosis	NA	NA	NA	NA	NA	NA	NA	NA

Abbreviations: CI, confidence interval; NA, not applicable.

Table S12 Sensitivity Analysis: Outcomes After Removing Individuals Studies

**Author, Year**	**Odds Ratio (95% CI)**	
	**Malignant Melanoma (All Subtype)**	**Non-melanoma Skin Cancer: Basal Cell Carcinoma**
**Overall**	**1.10 (1.04 – 1.15)**	**1.05 (1.02 – 1.09)**
Westerdahl et al[1], 1996	1.10 (1.04 – 1.15)	NA
Jensen et al[2], 2008	1.09 (1.04 – 1.15)	1.06 (1.02 – 1.10)
Kaae et al[3], 2010	1.09 (1.04 – 1.15)	1.06 (1.02 – 1.10)
Ruiter et al[4], 2010	NA	1.06 (1.02 – 1.10)
de Vries et al[5], 2012	1.10 (1.04 – 1.15)	1.05 (1.02 – 1.09)
Friedman et al[6], 2012	NA	NA
Traianou et al[7], 2012	NA	NA
Robinson et al[8], 2013	NA	NA
Schmidt et al[9], 2015	1.10 (1.04 – 1.16)	1.05 (1.02 – 1.09)
Nardone et al[10], 2017	1.09 (1.04 – 1.15)	1.04 (1.01 – 1.08)
Pottegård et al[11], 2017	NA	NA
Pedersen et al[12], 2018	NA	1.05 (1.01 – 1.09)
Pottegård et al[13], 2018	1.09 (1.03 – 1.15)	NA
Shaw et al[14], 2018	NA	NA
Su et al[15], 2018	NA	NA
Pedersen et al[16], 2019	NA	NA
Pottegård et al[17], 2019	1.12 (1.06 – 1.17)	NA
Daniels et al[18], 2020	1.09 (1.04 – 1.15)	NA
Lee et al[19], 2020	1.10 (1.04 – 1.15)	NA
Letellier et al[20], 2020	NA	1.06 (1.02 – 1.09)
Morales et al[21], 2020	1.10 (1.04 – 1.15)	1.05 (1.01 – 1.09)
Park et al[22], 2020	1.12 (1.06 – 1.17)	NA
Yeon et al[23], 2020	NA	NA
Adalsteinsson et al[24], 2021	NA	1.05 (1.01 – 1.09)
de Haan-Du et al[25], 2021	NA	NA
Drucker et al[26], 2021	1.09 (1.04 – 1.15)	NA
Eworuke et al[27], 2021	NA	1.06 (1.03 – 1.09)
Habel et al[28], 2021	1.10 (1.04 – 1.16)	NA
Kim et al[29], 2021	NA	NA
León-Muñoz et al[30], SIDIAP cohort 2021	1.09 (1.03 – 1.14)	NA
León-Muñoz et al[30], BIFAP cohort 2021	1.09 (1.04 – 1.15)	1.05 (1.01 – 1.09)
Rouette et al[31], 2021	1.10 (1.05 – 1.16)	1.06 (1.02 – 1.09)
Schneider et al[32], 2021	1.10 (1.03 – 1.17)	1.05 (1.01 – 1.09)

Abbreviations: BIFAP, Base de Datos para la Investigación Farmacoepidemiológica en Atención Primaria; CI, confidence interval; NA, not applicable; SIDIAP, Spain: Information System for Research in Primary Care.

Table S12 Sensitivity Analysis: Outcomes After Removing Individuals Studies (Continued)

**Author, Year**	**Odds Ratio (95% CI)**	
	**Non-melanoma Skin Cancer: Squamous Cell Carcinoma**	**Non-melanoma Skin Cancer: Unspecified**
**Overall**	**1.35 (1.22 – 1.48)**	**1.08 (1.03 – 1.12)**
Westerdahl et al[1], 1996	NA	NA
Jensen et al[2], 2008	1.35 (1.22 – 1.50)	NA
Kaae et al[3], 2010	1.37 (1.24 – 1.51)	NA
Ruiter et al[4], 2010	NA	NA
de Vries et al[5], 2012	1.33 (1.21 – 1.47)	NA
Friedman et al[6], 2012	NA	NA
Traianou et al[7], 2012	NA	NA
Robinson et al[8], 2013	1.35 (1.22 – 1.48)	NA
Schmidt et al[9], 2015	1.37 (1.24 – 1.52)	NA
Nardone et al[10], 2017	1.30 (1.18 – 1.43)	NA
Pottegård et al[11], 2017	NA	NA
Pedersen et al[12], 2018	1.29 (1.19 – 1.41)	NA
Pottegård et al[13], 2018	NA	NA
Shaw et al[14], 2018	NA	NA
Su et al[15], 2018	1.36 (1.23 – 1.51)	NA
Pedersen et al[16], 2019	NA	NA
Pottegård et al[17], 2019	NA	1.07 (1.01 – 1.13)
Daniels et al[18], 2020	NA	NA
Lee et al[19], 2020	NA	1.08 (1.04 – 1.13)
Letellier et al[20], 2020	1.33 (1.21 – 1.46)	NA
Morales et al[21], 2020	1.35 (1.22 – 1.49)	NA
Park et al[22], 2020	NA	1.11 (1.08 – 1.13)
Yeon et al[23], 2020	NA	NA
Adalsteinsson et al[24], 2021	1.36 (1.23 – 1.51)	NA
de Haan-Du et al[25], 2021	1.31 (1.19 – 1.44)	NA
Drucker et al[26], 2021	NA	1.07 (1.02 – 1.13)
Eworuke et al[27], 2021	1.38 (1.24 – 1.53)	NA
Habel et al[28], 2021	NA	NA
Kim et al[29], 2020	NA	1.07 (1.03 – 1.12)
León-Muñoz et al[30], SIDIAP cohort 2021	NA	1.06 (1.01 – 1.12)
León-Muñoz et al[30], BIFAP cohort 2021	1.35 (1.22 – 1.49)	1.07 (1.01 – 1.13)
Rouette et al[31], 2021	1.34 (1.21 – 1.48)	NA
Schneider et al[32], 2021	1.39 (1.20 – 1.60)	NA

Abbreviations: BIFAP, Base de Datos para la Investigación Farmacoepidemiológica en Atención Primaria; CI, confidence interval; NA, not applicable; SIDIAP, Spain: Information System for Research in Primary Care.

Table S13 Meta-Regression of Included Studies

Covariate	Malignant Melanoma (All Subtype)			Non-melanoma Skin Cancer: Basal Cell Carcinoma		
	**No. of Studies (Reference)**	**Odd Ratio (95% CI)**[†]	***P* Value**	**No. of Studies (Reference)**	**Odd Ratio (95% CI)**[†]	***P* Value**
**Risk of bias assessment**						
NOS (per 1 point)	17 (1, 2, 3, 5, 9, 10, 13, 17, 18, 19, 21, 22, 26, 28, 30, 31, 32)	0.97 (0.84 – 1.11)	0.651	14 (2, 3, 4, 5, 9, 10, 12, 20, 21, 24, 27, 30, 31, 32)	0.99 (0.90 – 1.09)	0.826
**Study characteristics**						
Sample size (<10,000 vs. ≥10,000)	17 (1, 2, 3, 5, 9, 10, 13, 17, 18, 19, 21, 22, 26, 28, 30, 31, 32)	0.89 (0.69 – 1.14)	0.331	14 (2, 3, 4, 5, 9, 10, 12, 20, 21, 24, 27, 30, 31, 32)	1.04 (0.85 – 1.26)	0.672
Study design (case-control vs. cohort)	17 (1, 2, 3, 5, 9, 10, 13, 17, 18, 19, 21, 22, 26, 28, 30, 31, 32)	0.94 (0.81 – 1.09)	0.381	14 (2, 3, 4, 5, 9, 10, 12, 20, 21, 24, 27, 30, 31, 32)	0.96 (0.87 – 1.06)	0.419
Study locations (European/North America vs. International/Other)	17 (1, 2, 3, 5, 9, 10, 13, 17, 18, 19, 21, 22, 26, 28, 30, 31, 32)	**0.84 (0.74 – 0.96)**	**0.014**	14 (2, 3, 4, 5, 9, 10, 12, 20, 21, 24, 27, 30, 31, 32)	1.21 (0.66 – 2.23)	0.508
**Baseline study level**						
Age (mean, per 1 year)	10 (5, 17, 18, 19, 21, 22, 26, 30, 31, 32)	1.01 (0.99 – 1.02)	0.247	10 (4, 5, 12, 20, 21, 24, 27, 30, 31, 32)	1.00 (0.99 – 1.01)	0.286
Female sex (per %)	13 (5, 9, 10, 17, 18, 19, 21, 22, 26, 28, 30, 31, 32)	1.00 (0.99 – 1.01)	0.734	12 (4, 5, 9, 10, 12, 20, 21, 24, 27, 30, 31, 32)	1.01 (0.99 – 1.03)	0.154
Thiazide diuretics utilization (per %)	16 (1, 2, 5, 9, 10, 13, 17, 18, 19, 21, 22, 26, 28, 30, 31, 32)	1.00 (0.99 – 1.00)	0.124	12 (2, 5, 9, 10, 12, 20, 21, 24, 27, 30, 31, 32)	1.00 (0.99 – 1.00)	0.411
Immunosuppressant (per %)	10 (5, 13, 18, 19, 21, 22, 26, 30, 31, 32)	0.90 (0.78 – 1.03)	1.09	9 (5, 12, 20, 21, 24, 27, 30, 31, 32)	1.00 (1.00 – 1.00)	0.910
RAS inhibitors (per %)	6 (9, 10, 18, 30, 31, 32)	1.00 (0.99 – 1.01)	0.882	6 (9, 10, 27, 30, 31, 32)	0.99 (0.98 – 1.01)	0.213
NSAIDs (per %)	9 (5, 9, 17, 18, 19, 21, 30, 31, 32)	1.00 (0.99 – 1.00)	0.124	7 (5, 9, 12, 21, 30, 31, 32)	1.00 (1.00 – 1.00)	0.793

[†]Effect size for each variable of interest reflecting unit change.

Abbreviations: CI, confidence interval; NOS, Newcastle-Ottawa scale; NSAIDs, non-steroidal anti-inflammatory drugs; RAS, renin-angiotensin system.

Table S13 Meta-Regression of Included Studies

**Covariate**	**Non-melanoma Skin Cancer: Squamous Cell Carcinoma**			**Non-melanoma Skin Cancer: Unspecified**		
	**No. of Studies (Reference)**	**Odd Ratio (95% CI)†**	***P* Value**	**No. of Studies (Reference)**	**Odd Ratio (95% CI)†**	***P* Value**
**Risk of bias assessment**						
NOS (per 1 point)	16 (2, 3, 5, 8, 9, 10, 12, 15, 20, 21, 24, 27, 29, 30, 31, 32)	0.96 (0.73 – 1.25)	0.721	6 (17, 19, 22, 26, 29, 30)	NA	NA
**Study characteristics**						
Sample size (<10,000 vs. ≥10,000)	16 (2, 3, 5, 8, 9, 10, 12, 15, 20, 21, 24, 25, 27, 30, 31, 32)	0.90 (0.58 – 1.41)	0.633	6 (17, 19, 22, 26, 29, 30)	NA	NA
Study design (case-control vs. cohort)	16 (2, 3, 5, 8, 9, 10, 12, 15, 20, 21, 24, 25, 27, 30, 31, 32)	1.12 (0.79 – 1.59)	0.509	6 (17, 19, 22, 26, 29, 30)	0.92 (0.82 – 1.04)	0.146
Study locations (European vs. North America vs. International/Other)	16 (2, 3, 5, 8, 9, 10, 12, 15, 20, 21, 24, 25, 27, 30, 31, 32)	1.21 (0.55 – 2.69)	0.613	6 (17, 19, 22, 26, 29, 30)	0.94 (0.81 – 1.09)	0.319
**Baseline study level**						
Age (mean, per 1 year)	11 (5, 12, 15, 20, 21, 24, 25, 27, 30, 31, 32)	1.00 (0.98 – 1.02)	0.736	5 (17, 19, 22, 26, 30)	**1.01 (1.00 – 1.01)**	**0.010**
Female sex (per %)	14 (5, 8, 9, 10, 12, 15, 20, 21, 24, 25, 27, 30, 31, 32)	1.01 (0.98 – 1.04)	0.565	6 (17, 19, 22, 26, 29, 30)	1.00 (0.99 – 1.02)	0.669
Thiazide diuretics utilization (per %)	15 (2, 5, 8, 9, 10, 12, 15, 20, 21, 24, 25, 27, 30, 31, 32)	1.00 (0.99 – 1.01)	0.485	6 (17, 19, 22, 26, 29, 30)	1.00 (0.99 – 1.00)	0.148
Immunosuppressant (per %)	9 (5, 12, 20, 21, 24, 27, 30, 31, 32)	1.00 (0.99 – 1.01)	0.490	4 (19, 22, 26, 30)	NA	NA
RAS inhibitors (per %)	8 (9, 10, 15, 25, 27, 30, 31, 32)	0.99 (0.97 – 1.01)	0.314	1 (30)	NA	NA
NSAIDs (per %)	8 (5, 9, 12, 21, 25, 30, 31, 32)	1.00 (0.99 – 1.01)	0.753	4 (17, 19, 29, 30)	1.00 (0.99 – 1.01)	0.776

†Effect size for each variable of interest reflecting unit change.

Abbreviations: CI, confidence interval; NA, not applicable; NOS, Newcastle-Ottawa scale; NSAIDs, non-steroidal anti-inflammatory drugs; RAS, renin-angiotensin system.

Table S14 Meta-Analysis of Included Studies with Calibration for Publication Bias[†]

Skin Cancer	No. of Studies (Reference)	*P* Value for Begg's Test	*P* Value for Egger's Test	Odds Ratio (95% CI)	*P* Value	Heterogeneity			
						Q Statistic	*P* Value	I^2 Index (95% CI)	τ^2
**Primary outcomes**									
Malignant melanoma (all subtype)	17 (1, 2, 3, 5, 9, 10, 13, 17, 18, 19, 21, 22, 26, 28, 30, 31, 32)	0.705	0.463	1.07 (1.02 – 1.12)	0.005	74.58	<0.001	70.5% (52.3 – 79.7)	0.006
Non-melanoma skin cancer: basal cell carcinoma	14 (2, 3, 4, 5, 9, 10, 12, 20, 21, 24, 27, 30, 31, 32)	1.000	0.282	Not detected	NA	NA	NA	NA	NA
Non-melanoma skin cancer: squamous cell carcinoma	16 (2, 3, 5, 8, 9, 10, 12, 15, 20, 21, 24, 25, 27, 30, 31, 32)	0.964	0.212	Not detected	NA	NA	NA	NA	NA
Non-melanoma skin cancer: Unspecified	6 (17, 19, 22, 26, 29, 30)	0.368	0.297	1.07 (1.02 – 1.12)	0.002	39.03	<0.001	82.1% (62.3 – 89.2)	0.002
**Secondary outcomes**									
Lip cancer	5 (6, 11, 17, 18, 21)	0.806	0.625	1.85 (1.48 – 2.32)	<0.001	9.85	0.079	49.3% (0.0 – 78.0)	0.034
Merkel cell carcinoma	2 (3, 16)	1.000	NA	Not detected	NA	NA	NA	NA	NA
Malignant adnexal skin tumors	1 (16)	NA	NA	NA	NA	NA	NA	NA	NA
Oral cavity cancer	1 (21)	NA	NA	NA	NA	NA	NA	NA	NA
Actinic keratosis	1 (7)	NA	NA	NA	NA	NA	NA	NA	NA

[†]Calibration for publication bias was carried out, if indicated in trim and fill analysis.

Abbreviations: CI, confidence interval; NA, not applicable.

Table S15 Quality of Evidence Synthesis and GRADE Evidence Profile of Outcomes

Outcomes	No. of Studies (Ref.)	Study Design (Sample Size)	Quality Assessment: Required Domains					Other Issues	Finding and Direction (Magnitude) of Effect	Strength of Evidence
			Study Limitations	Directions	Consistency	Precision	Reporting Bias			
**Malignant melanoma**										
All subtype	17 (1, 2, 3, 5, 9, 10, 13, 17, 18, 19, 21, 22, 26, 28, 30, 31, 32)	Non-RCTs (10129196)	High	Direct	Consistent	Imprecise	Undetected	• Dose-response association could not be determined	• Seventeen non-RCTs with a large sample size revealed high study limitations and imprecise based on the prediction interval (0.93 – 1.29).<br>• The summary pooled OR was 1.10 (95% CI, 1.04 – 1.15); *P*<0.001).<br>• Weak strength of association (magnitude of effect) with moderate heterogeneity (I^2 index=73.4%).<br>• The findings were robust with respect to a set of sensitivity analyses.	Very low (small harmful)
Superficial spreading melanoma	3 (13, 18, 28)	Non-RCTs (221624)	High	Direct	Unknown	Imprecise	Suspected	• Dose-response association could not be determined<br>• Present plausible confounding that would decrease the observed effect[†]	• Three non-RCTs illustrated high study limitations and imprecise based on the prediction interval (0.35 – 4.02).<br>• The summary pooled OR was 1.18 (95% CI, 1.05 – 1.33); *P*=0.006).<br>• Weak strength of association (magnitude of effect) with low heterogeneity (I^2 index=53.7%).<br>• Publication bias cannot be ruled out due to the small number of studies included.	Very low (small harmful)

[†]On the basis of the E-value.

Abbreviations: CI, confidence interval; GRADE, Grading of Recommendations Assessment, Development and Evaluation; OR, odds ratio; RCTs, randomized-controlled trials.

Table S15 Quality of Evidence Synthesis and GRADE Evidence Profile of Outcomes (Continued)

Outcomes	No. of Studies (Ref.)	Study Design (Sample Size)	Quality Assessment: Required Domains					Other Issues	Finding and Direction (Magnitude) of Effect	Strength of Evidence
			Study Limitations	Directions	Consistency	Precision	Reporting Bias			
**Malignant melanoma (continued)**										
Nodular melanoma	3 (13, 18, 28)	Non-RCTs (36631)	High	Direct	Unknown	Imprecise	Suspected	• Dose-response association could not be determined<br>• Weak strength of association (magnitude of effect)<br>• Present plausible confounding that would decrease the observed effect†	• Three non-RCTs illustrated high study limitations and imprecise based on the prediction interval (0.54 – 2.79).<br>• The summary pooled OR was 1.23 (95% CI, 1.08 – 1.40); *P*=0.001).<br>• Weak strength of association (magnitude of effect) with moderate heterogeneity (I^2 index=0.0%).<br>• Publication bias cannot be ruled out due to the small number of studies included.	Very low (small harmful)
Lentigo maligna melanoma	3 (13, 18, 28)	Non-RCTs (21407)	High	Direct	Unknown	Imprecise	Suspected	• Dose-response association could not be determined<br>• Present plausible confounding that would decrease the observed effect†	• Three non-RCTs illustrated high study limitations and imprecise based on the prediction interval (0.18 – 10.09).<br>• The summary pooled OR was 1.33 (95% CI, 1.08 – 1.65); *P*=0.008).<br>• Weak strength of association (magnitude of effect) with moderate heterogeneity (I^2 index=36.9%).<br>• Publication bias cannot be ruled out due to the small number of studies included.	Very low (small harmful)

†On the basis of the E-value.

Abbreviations: CI, confidence interval; GRADE, Grading of Recommendations Assessment, Development and Evaluation; OR, odds ratio; RCTs, randomized-controlled trials.

Table S15 Quality of Evidence Synthesis and GRADE Evidence Profile of Outcomes (Continued)

Outcomes	No. of Studies (Ref.)	Study Design (Sample Size)	Quality Assessment: Required Domains					Other Issues	Finding and Direction (Magnitude) of Effect	Strength of Evidence
			Study Limitations	Directions	Consistency	Precision	Reporting Bias			
**Non-melanoma skin cancer**										
Basal cell carcinoma	14 (2, 3, 4, 5, 9, 10, 12, 20, 21, 24, 27, 30, 31, 32)	Non-RCTs (19780476)	High	Direct	Consistent	Imprecise	Undetected	• Dose-response association could not be determined	• Fourteen non-RCTs with a large sample size revealed high study limitations and imprecise based on the prediction interval (0.94 – 1.19).<br>• The summary pooled OR was 1.05 (95% CI, 1.02 – 1.09); *P*=0.003).<br>• Weak strength of association (magnitude of effect) with high heterogeneity (I^2 index=87.2%).<br>• The findings were robust with respect to a set of sensitivity analyses.	Very low (small harmful)
Squamous cell carcinoma	16 (2, 3, 5, 8, 9, 10, 12, 15, 20, 21, 24, 25, 27, 30, 31, 32)	Non-RCTs (16387862)	High	Direct	Consistent	Imprecise	Undetected	• Dose-response association could not be determined	• Sixteen non-RCTs with a large sample size revealed high study limitations and imprecise based on the prediction interval (0.93 – 1.95).<br>• The summary pooled OR was 1.35 (95% CI, 1.22 – 1.48); *P*<0.001).<br>• Weak strength of association (magnitude of effect) with high heterogeneity (I^2 index=97.1%).<br>• The findings were robust with respect to a set of sensitivity analyses.	Very low (small harmful)

Abbreviations: CI, confidence interval; GRADE, Grading of Recommendations Assessment, Development and Evaluation; OR, odds ratio; RCTs, randomized-controlled trials.

Table S15 Quality of Evidence Synthesis and GRADE Evidence Profile of Outcomes (Continued)

Outcomes	No. of Studies (Ref.)	Study Design (Sample Size)	Quality Assessment: Required Domains					Other Issues	Finding and Direction (Magnitude) of Effect	Strength of Evidence
			Study Limitations	Directions	Consistency	Precision	Reporting Bias			
**Non-melanoma skin cancer (continued)**										
Unspecified	6 (17, 19, 22, 26, 29, 30)	Non-RCTs (5668737)	High	Direct	Consistent	Imprecise	Undetected	• Dose-response association could not be determined	• Six non-RCTs with a large sample size revealed high study limitations and imprecise based on the prediction interval (0.94 – 1.23).<br>• The summary pooled OR was 1.08 (95% CI, 1.03 – 1.12); *P*=0.001).<br>• Weak strength of association (magnitude of effect) with high heterogeneity (I^2 index=83.0%).<br>• The findings were robust with respect to a set of sensitivity analyses.	Very low (small harmful)
**Secondary outcomes**										
Lip cancer	5 (6, 11, 17, 18, 21)	Non-RCTs (161491)	High	Direct	Consistent	Imprecise	Undetected	• Dose-response association could not be determined<br>• Present plausible confounding that would decrease the observed effect[†]	• Five non-RCTs illustrated high study limitations and imprecise based on the prediction interval (0.97 – 3.81).<br>• The summary pooled OR was 1.92 (95% CI, 1.52 – 2.42); *P*<0.001).<br>• Weak strength of association (magnitude of effect) with moderate heterogeneity (I^2 index=51.5%).	Very low (small harmful)
MCC	2 (3, 16)	Non-RCTs (4763703)	High	Direct	Unknown	Unknown	Suspected	• Present plausible confounding that would decrease the observed effect[†]	• Two non-RCTs with a large sample size illustrated high study limitations; however, the uncertainty in terms of prediction interval could not be estimated.<br>• The summary pooled OR was 0.98 (95% CI, 0.57 – 1.65); *P*=0.924).	Insufficient data

[†]On the basis of the E-value.

Abbreviations: CI, confidence interval; GRADE, Grading of Recommendations Assessment, Development and Evaluation; OR, odds ratio; RCTs, randomized-controlled trials.

Table S15 Quality of Evidence Synthesis and GRADE Evidence Profile of Outcomes (Continued)

Outcomes	No. of Studies (Ref.)	Study Design (Sample Size)	Quality Assessment: Required Domains					Other Issues	Finding and Direction (Magnitude) of Effect	Strength of Evidence
			Study Limitations	Directions	Consistency	Precision	Reporting Bias			
**Secondary outcomes (continued)**										
MAST	1 (16)	Non-RCTs (2752)	High	Direct	Unknown	Unknown	Suspected	• Present plausible confounding that would decrease the observed effect†	• A single study with a small sample size by Pedersen et al[16], 2019 illustrated non-statistical significance (OR, 1.40; 95% CI, 0.86 – 2.29; *P*=0.179).<br>• However, the uncertainty in terms of prediction interval could not be estimated.	Insufficient data
Oral cavity cancer	1 (21)	Non-RCTs (73844)	High	Direct	Unknown	Unknown	Suspected	• Present plausible confounding that would decrease the observed effect†	• A single study by Morales et al[22], 2020 illustrated non-statistical significance (OR, 0.90; 95% CI, 0.60 – 1.36; *P*=0.614).<br>• However, the uncertainty in terms of prediction interval could not be estimated.	Insufficient data
Actinic keratosis	1 (7)	Non-RCTs (1029)	High	Direct	Unknown	Unknown	Suspected	• Present plausible confounding that would decrease the observed effect†	• A single study with a small sample size by Traianou et al[7], 2012 illustrated statistical significance (OR, 3.18; 95% CI, 1.93 – 5.25; *P*<0.001).<br>• However, the uncertainty in terms of prediction interval could not be estimated.	Insufficient data

†On the basis of the E-value.

Abbreviations: CI, confidence interval; GRADE, Grading of Recommendations Assessment, Development and Evaluation; OR, odds ratio; RCTs, randomized-controlled trials.

Figure S1 Study Selection Flowchart

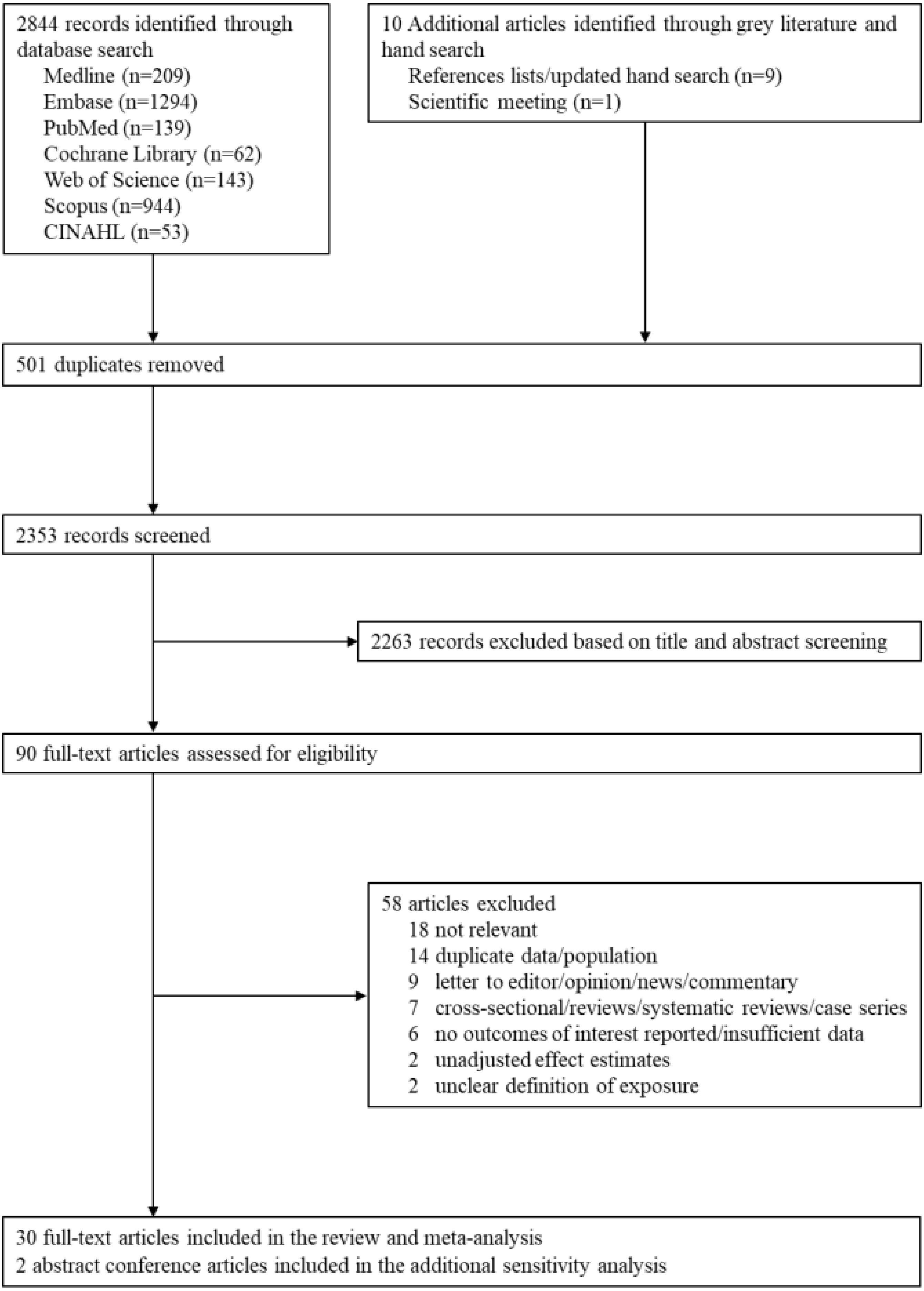

Figure S2 Use of Thiazide Diuretics and the Risk of Lip Cancer

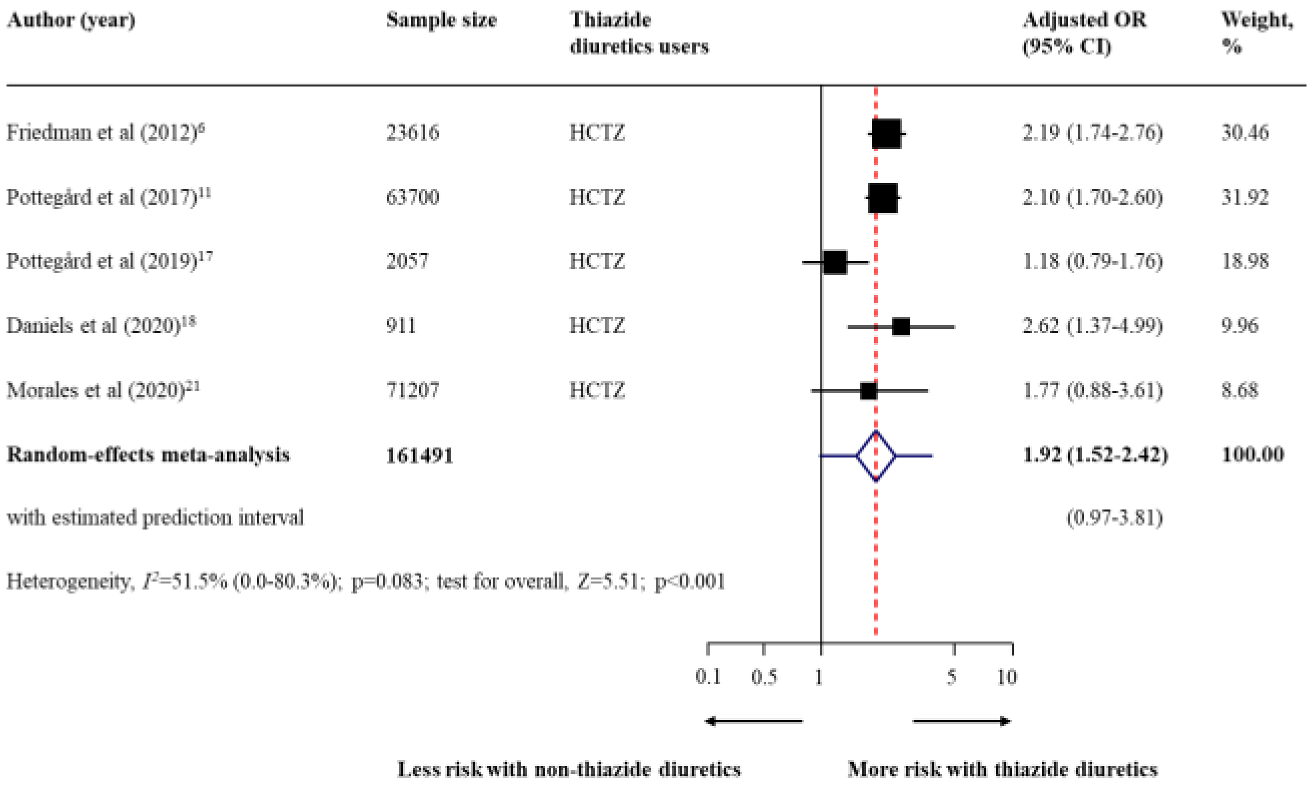

Abbreviations: CI, confidence interval, HCTZ, hydrochlorothiazide; OR, odds ratio.

Figure S3 Funnel Plot of Included Studies in the Meta-Analysis

A. Malignant Melanoma (All Subtype)

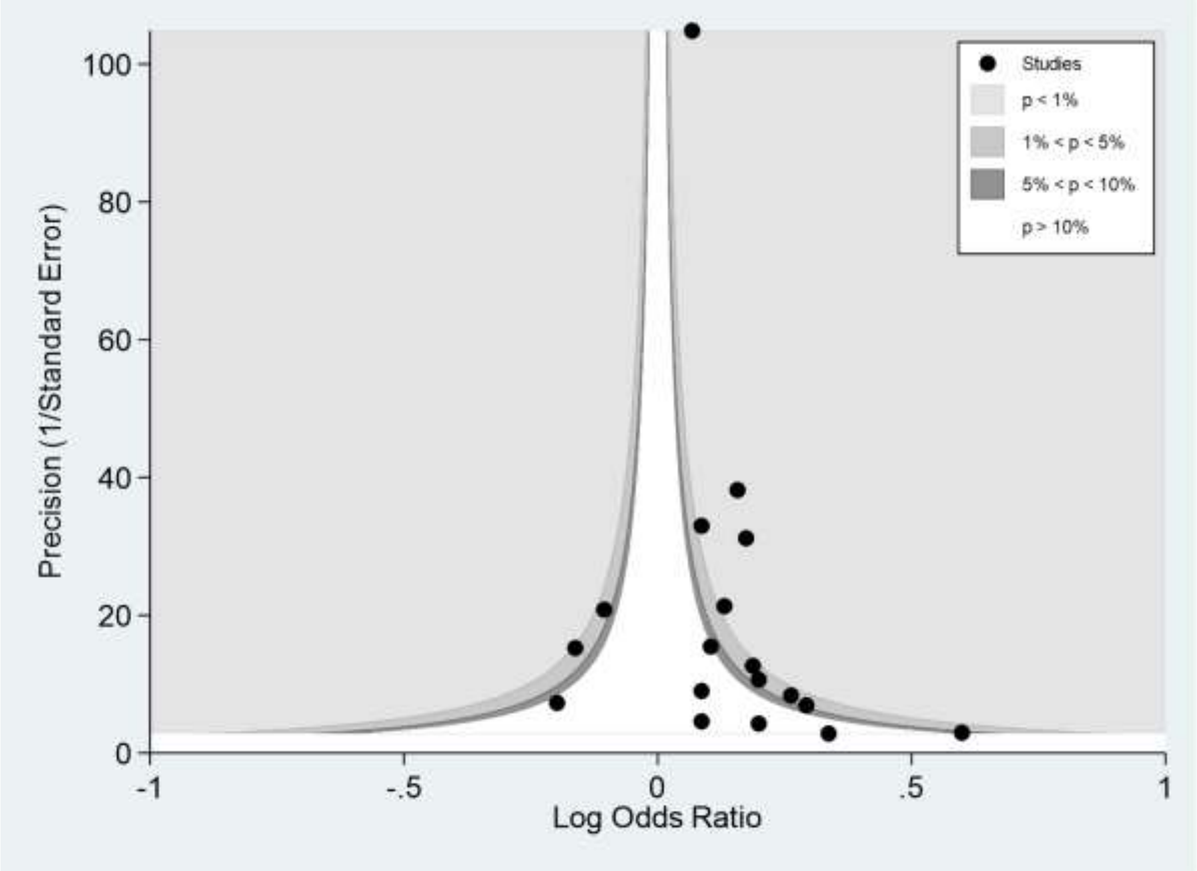

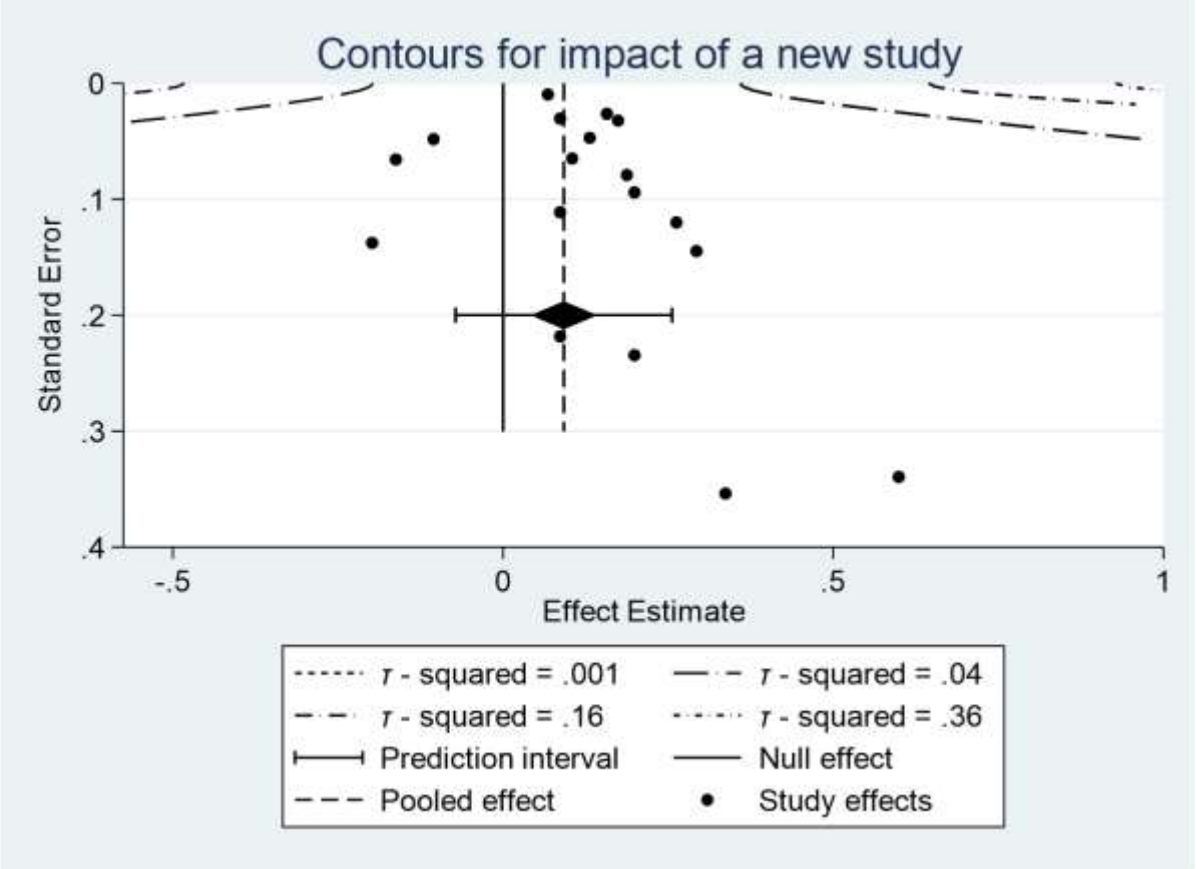

Figure S3 Funnel Plot of Included Studies in the Meta-Analysis (Continued)

B. Non-melanoma Skin Cancer: Basal Cell Carcinoma

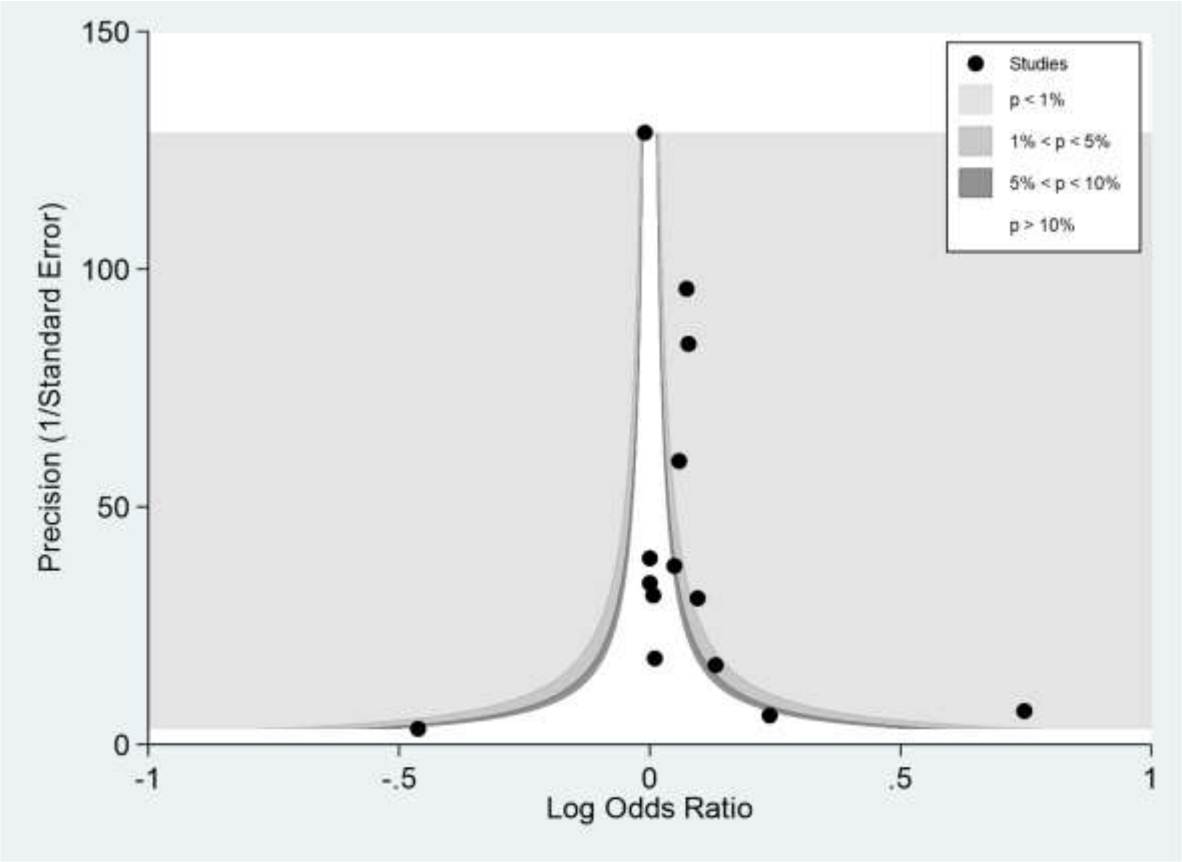

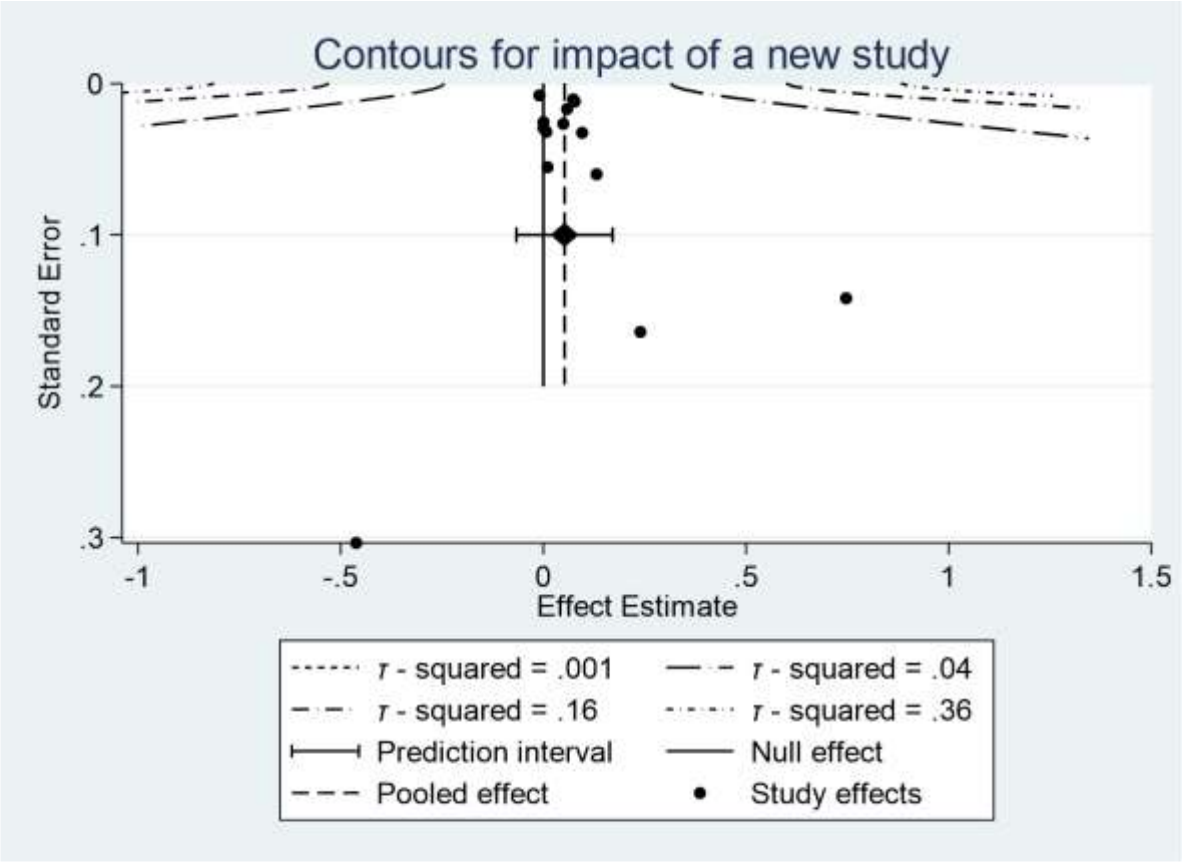

Figure S3 Funnel Plot of Included Studies in the Meta-Analysis (Continued)

C. Non-melanoma Skin Cancer: Squamous Cell Carcinoma

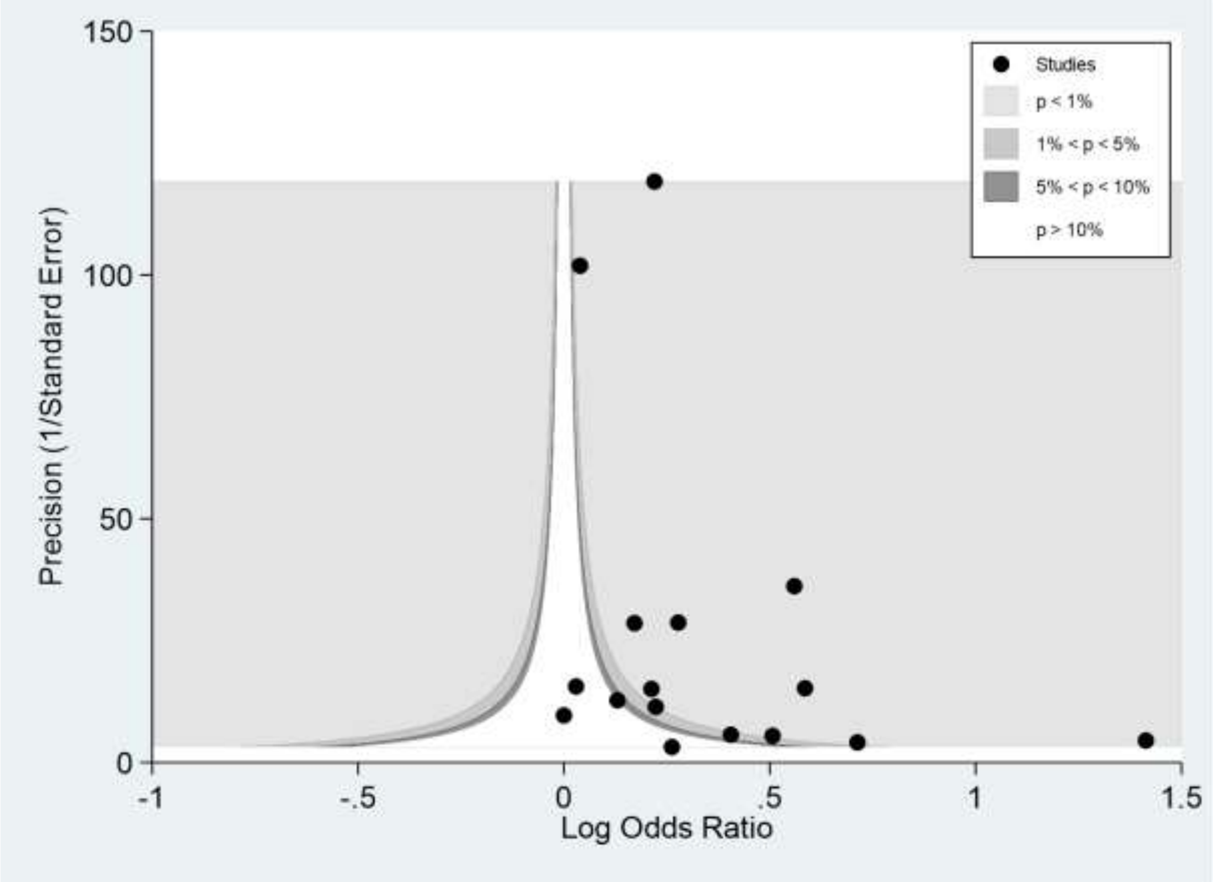

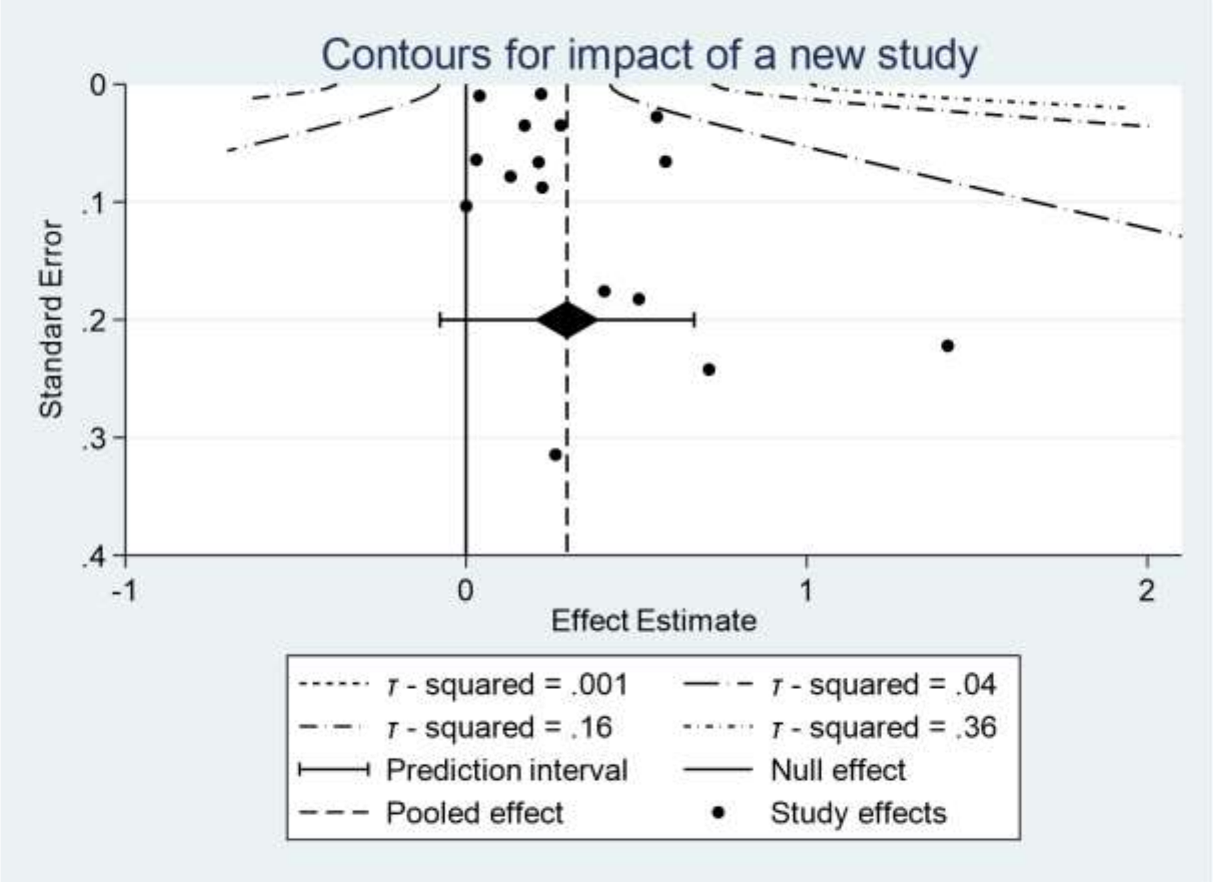

Figure S3 Funnel Plot of Included Studies in the Meta-Analysis (Continued)

D. Non-melanoma Skin Cancer: Unspecified

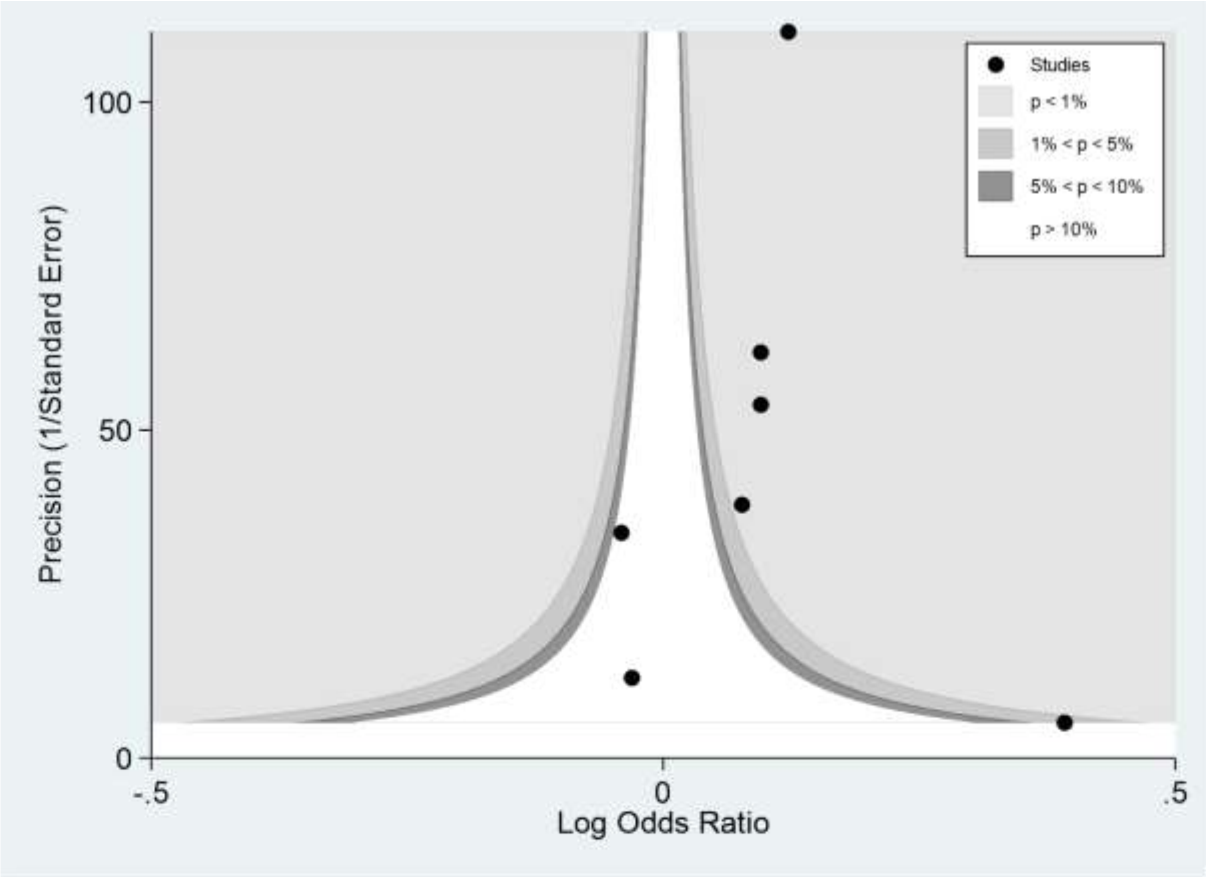

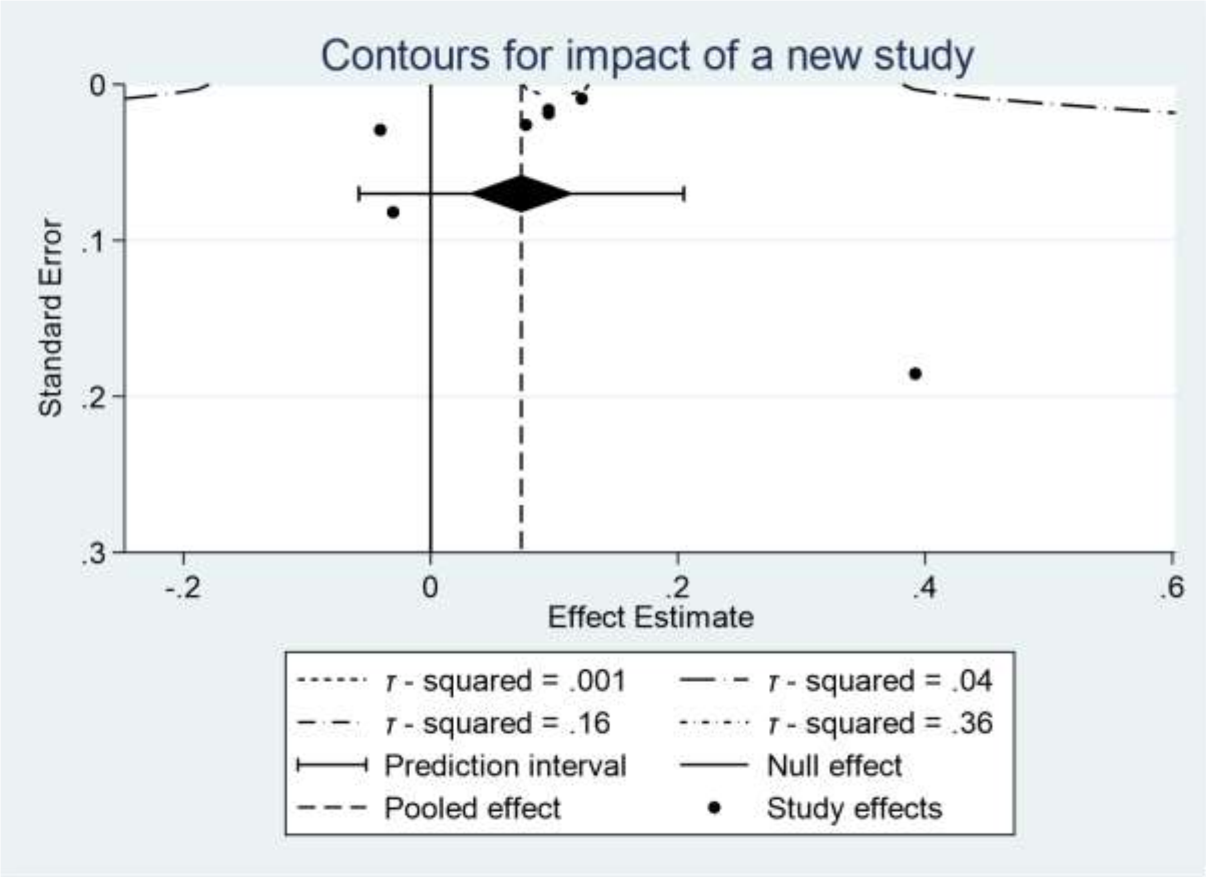

Figure S3 Funnel Plot of Included Studies in the Meta-Analysis (Continued)

E. Lip Cancer

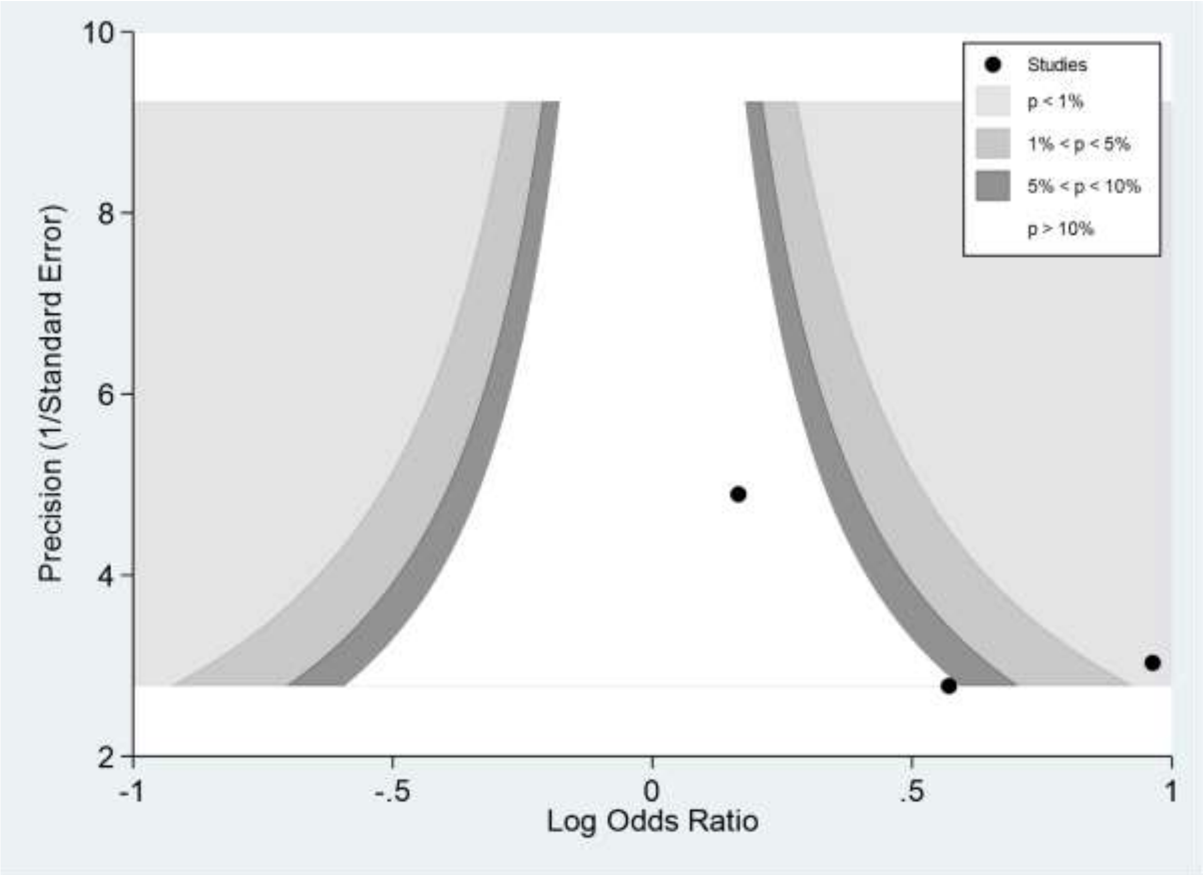

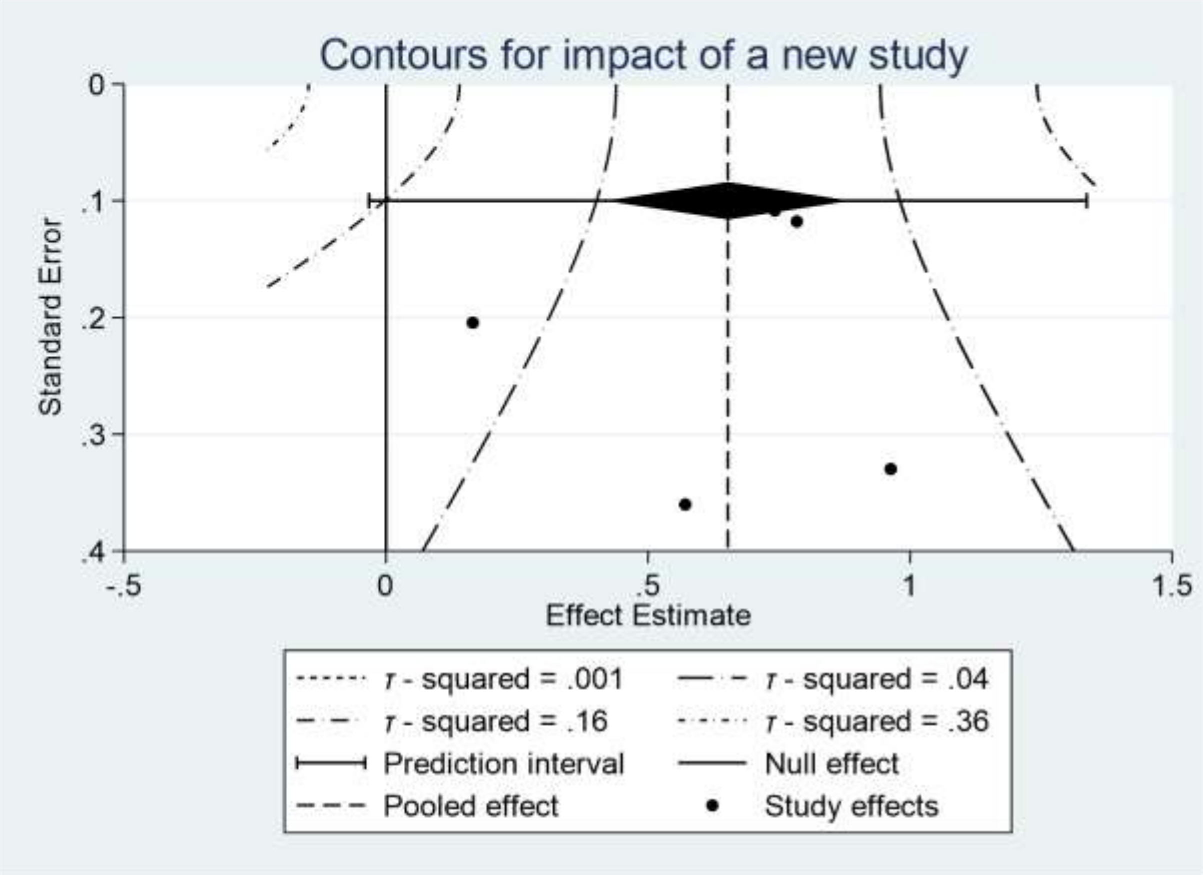

File S1 Meta-Analysis of Observational Studies in Epidemiology (MOOSE) statement Checklist

Item No	Recommendation	Reported on Page No
**Reporting of background should include**		
1	Problem definition	Page 3
2	Hypothesis statement	Page 3
3	Description of the study outcomes	Page 3, Table S2
4	Types of exposure or intervention	Page 3,4, Table S2
5	Type of study designs used	Page 3,4, Table S2
6	Study population	Page 3,4, Table S2
**Reporting of search strategy should include**		
7	Qualifications of searchers (eg. librarians and investigators)	Page 3
8	Search strategy, including time period included in the synthesis and keywords	Page 3, Table S1
9	Effort to include all available studies, including contact with authors	Page 4
10	Databases and registries searched	Page 3, Table S1
11	Search software used, name and version, including special features used (eg. explosion)	Table S1
12	Use of hand searching (eg. reference list of obtained articles)	Page 3
13	List of citations located and those excluded, including justification	Figure S1, eReferences
14	Method of addressing articles published in languages other than English	Page 3
15	Method of handling abstracts and unpublished studies	Page 5
16	Description of any contact with authors	Page 4
**Reporting of methods should include**		
17	Description of relevance or appropriateness of studies assembled for assessing the hypothesis to be tested	Page 4
18	Rationale for the selection and coding of data (eg. sound clinical principles or convenience)	Page 4
19	Documentation of how data were classified and coded (eg. multiple raters, blinding and interrater reliability)	Page 4
20	Assessment of confounding (eg. comparability of cases and controls in studies where appropriate)	Page 4

Stroup DF, Berlin JA, Morton SC, et al. Meta-analysis of observational studies in epidemiology: a proposal for reporting. Meta-analysis Of Observational Studies in Epidemiology (MOOSE) group. JAMA. 2000;283(15):2008-12.

File S1 Meta-Analysis of Observational Studies in Epidemiology (MOOSE) statement Checklist (Continued)

Item No	Recommendation	Reported on Page No
**Reporting of methods should include (Continued)**		
21	Assessment of study quality, including blinding of quality assessors; stratification or regression on possible predictors of study results	Page 4
22	Assessment of heterogeneity	Page 5
23	Description of statistical methods (eg. complete description of fixed or random effects models, justification of whether the chosen models account for predictions of study results, dose-response models, or cumulative meta-analysis) in sufficient detail to be replicated	Page 4,5
24	Provision of appropriate tables and graphics	Throughout tables/figures
**Reporting of results should include**		
25	Graph summarizing individual study estimates and overall estimate	Throughout figures
26	Table giving descriptive information for each study included	Throughout tables
27	Results of sensitivity testing (eg. subgroup analysis)	Page 11,12
28	Indication of statistical uncertainty of findings	Page 12, Table 2
**Reporting of discussion should include**		
29	Quantitative assessment of bias (eg. publication bias)	Page 13
30	Justification for exclusion (eg. exclusion of non-English language citations)	Not applicable
31	Assessment of quality of included studies	Page 13
	Reporting of conclusion should include	Page 14
32	Consideration of alternative explanations for observed results	Page 12,13
33	Generalization of the conclusions (eg. appropriate for the data presented and within the domain of the literature review)	Page 14
34	Guidelines for future research	Page 14
35	Disclosure of funding source	Page 14

Stroup DF, Berlin JA, Morton SC, et al. Meta-analysis of observational studies in epidemiology: a proposal for reporting. Meta-analysis Of Observational Studies in Epidemiology (MOOSE) group. JAMA. 2000;283(15):2008-12.

File S2 PRISMA 2020 Statement Checklist

Section and Topic	Item #	Checklist item	Location where item is reported
**TITLE**			
Title	1	Identify the report as a systematic review.	Page 1
**ABSTRACT**			
Abstract	2	See the PRISMA 2020 for Abstracts checklist.	Page 1, 2
**INTRODUCTION**			
Rationale	3	Describe the rationale for the review in the context of existing knowledge.	Page 3
Objectives	4	Provide an explicit statement of the objective(s) or question(s) the review addresses.	Page 3
**METHODS**			
Eligibility criteria	5	Specify the inclusion and exclusion criteria for the review and how studies were grouped for the syntheses.	Page 3,4, Table S2
Information sources	6	Specify all databases, registers, websites, organisations, reference lists and other sources searched or consulted to identify studies. Specify the date when each source was last searched or consulted.	Page 3, Table S1
Search strategy	7	Present the full search strategies for all databases, registers and websites, including any filters and limits used.	Table S1
Selection process	8	Specify the methods used to decide whether a study met the inclusion criteria of the review, including how many reviewers screened each record and each report retrieved, whether they worked independently, and if applicable, details of automation tools used in the process.	Page 4
Data collection process	9	Specify the methods used to collect data from reports, including how many reviewers collected data from each report, whether they worked independently, any processes for obtaining or confirming data from study investigators, and if applicable, details of automation tools used in the process.	Page 4
Data items	10a	List and define all outcomes for which data were sought. Specify whether all results that were compatible with each outcome domain in each study were sought (e.g. for all measures, time points, analyses), and if not, the methods used to decide which results to collect.	Page 4
	10b	List and define all other variables for which data were sought (e.g. participant and intervention characteristics, funding sources). Describe any assumptions made about any missing or unclear information.	Page 4
Study risk of bias assessment	11	Specify the methods used to assess risk of bias in the included studies, including details of the tool(s) used, how many reviewers assessed each study and whether they worked independently, and if applicable, details of automation tools used in the process.	Pahe 4
Effect measures	12	Specify for each outcome the effect measure(s) (e.g. risk ratio, mean difference) used in the synthesis or presentation of results.	Page 4
Synthesis methods	13a	Describe the processes used to decide which studies were eligible for each synthesis (e.g. tabulating the study intervention characteristics and comparing against the planned groups for each synthesis (item #5)).	
	13b	Describe any methods required to prepare the data for presentation or synthesis, such as handling of missing summary statistics, or data conversions.	Page 4,5
	13c	Describe any methods used to tabulate or visually display results of individual studies and syntheses.	Page 4,5
	13d	Describe any methods used to synthesize results and provide a rationale for the choice(s). If meta-analysis was performed, describe the model(s), method(s) to identify the presence and extent of statistical heterogeneity, and software package(s) used.	Page 4,5
	13e	Describe any methods used to explore possible causes of heterogeneity among study results (e.g. subgroup analysis, meta-regression).	Page 5
	13f	Describe any sensitivity analyses conducted to assess robustness of the synthesized results.	Page 5

Section and Topic	Item #	Checklist item	Location where item is reported
Reporting bias assessment	14	Describe any methods used to assess risk of bias due to missing results in a synthesis (arising from reporting biases).	NA
Certainty assessment	15	Describe any methods used to assess certainty (or confidence) in the body of evidence for an outcome.	Page 5
**RESULTS**			
Study selection	16a	Describe the results of the search and selection process, from the number of records identified in the search to the number of studies included in the review, ideally using a flow diagram.	Page 6, Figure S1
	16b	Cite studies that might appear to meet the inclusion criteria, but which were excluded, and explain why they were excluded.	Page 6
Study characteristics	17	Cite each included study and present its characteristics.	Page 6, Table 1
Risk of bias in studies	18	Present assessments of risk of bias for each included study.	Page 6, Table S7
Results of individual studies	19	For all outcomes, present, for each study: (a) summary statistics for each group (where appropriate) and (b) an effect estimate and its precision (e.g. confidence/credible interval), ideally using structured tables or plots.	Table 2,<br>Figure 1-4
Results of syntheses	20a	For each synthesis, briefly summarise the characteristics and risk of bias among contributing studies.	Page 8,9
	20b	Present results of all statistical syntheses conducted. If meta-analysis was done, present for each the summary estimate and its precision (e.g. confidence/credible interval) and measures of statistical heterogeneity. If comparing groups, describe the direction of the effect.	Page 8,9, Table 2
	20c	Present results of all investigations of possible causes of heterogeneity among study results.	Page 11,12
	20d	Present results of all sensitivity analyses conducted to assess the robustness of the synthesized results.	Page 11,12
Reporting biases	21	Present assessments of risk of bias due to missing results (arising from reporting biases) for each synthesis assessed.	NA
Certainty of evidence	22	Present assessments of certainty (or confidence) in the body of evidence for each outcome assessed.	Table 2
**DISCUSSION**			
Discussion	23a	Provide a general interpretation of the results in the context of other evidence.	Page 12
	23b	Discuss any limitations of the evidence included in the review.	Page 12,13
	23c	Discuss any limitations of the review processes used.	Page 13
	23d	Discuss implications of the results for practice, policy, and future research.	Page 15
**OTHER INFORMATION**			
Registration and protocol	24a	Provide registration information for the review, including register name and registration number, or state that the review was not registered.	Page 3
	24b	Indicate where the review protocol can be accessed, or state that a protocol was not prepared.	Page 3
	24c	Describe and explain any amendments to information provided at registration or in the protocol.	Page 3, Appendix III
Support	25	Describe sources of financial or non-financial support for the review, and the role of the funders or sponsors in the review.	Page 14
Competing interests	26	Declare any competing interests of review authors.	Page 15
Availability of data, code and other materials	27	Report which of the following are publicly available and where they can be found: template data collection forms; data extracted from included studies; data used for all analyses; analytic code; any other materials used in the review.	NA

Page MJ, McKenzie JE, Bossuyt PM, Boutron I, Hoffmann TC, Mulrow CD, et al. The PRISMA 2020 statement: an updated guideline for reporting systematic reviews. BMJ 2021;372:n71.

File S3 Pre-Specified Protocol and Protocol Amendments

PROSPERO International prospective register of systematic reviews

The Association Between Thiazide Diuretics Use and the Risk of Skin Cancer: A Systematic Review and Meta-Analysis

Funding sources/sponsors
Chiang Mai University.

Conflicts of interest
No conflict of interest to disclose.

Review question(s)
To systematically review and synthesize the association between the use of thiazide diuretics and the risk of skin cancer.

Searches
An experienced information specialist will conduct electronic search strategies using an iterative process and in collaboration with the search team. Electronic databases, including PubMed, Medline, Embase, ~~Web of Science~~, Scopus, CINAHL, and CENTRAL—Cochrane library will be used to identify all relevant abstracts. The search strategy will be included the pharmacological class of thiazide or thiazide-like diuretics (Bendroflumethiazide, Benzthiazide, Chlorthalidone, Chlorothiazide, Cyclothiazide, Hydrochlorothiazide, Hydroflumethiazide, Indapamide, Metolazone, Methyclothiazide, Quinethazone) AND "skin cancer" or "squamous cell carcinoma" or "melanoma" or non-melanoma" or "basal cell carcinoma" or malignant melanoma", without language restriction. Key health and dermatology journals will be also manually searched.

Reference lists of included studies, previous systematic reviews, grey literature from Google Scholar, and preprint data (medRxiv and bioRxiv) will be supplemented to the bibliographic database searches. Furthermore, pre-planned updated searches will be performed.

Condition or domain being studied
Skin cancer among patients received thiazide diuretics for any indication.

Participants/ population
Adult and adolescent patients aged 12 ~~18~~ or more regardless of comorbid conditions will be included without geographical restriction.

Intervention(s), exposure(s)
Thiazide treatment in any indication and duration.

Comparator(s)/ control
Non-thiazide users or intensive exposure versus fewer lifetime exposures to thiazide diuretics.

Types of study to be included
Published randomized controlled trials (RCTs), quasi-RCT, and comparative effectiveness observational non-randomized studies (cohort studies and case-control studies) in any setting and

context. Crossover, cross-sectional, N of one trial, case series/case reports, animal studies, and in vitro studies will be excluded.

Main outcome(s)
Association of the use of thiazide diuretics and specific skin cancer types, including malignant melanoma (specific subtypes, superficial spreading melanoma, nodular melanoma, lentigo maligna melanoma) and non-melanoma skin cancer (basal cell carcinoma, squamous cell carcinoma, and unspecified non-melanoma).

Measures of effect
Risk of specific cancer types, hazard ratios (HRs), risk ratios (RRs), or odds ratios (ORs): all-time points as defined by the specific studies.

Additional outcome(s)
(i) Risk of new premalignant/precursor lesions (actinic keratosis or Bowen's disease)
(ii) ~~Skin cancer-related mortality~~ Other forms of skin cancer (lip cancer, Merkel cell carcinoma, malignant adnexal skin tumor, and oral cavity cancer).

Measures of effect
Risk of specific cancer types, hazard ratios (HRs), risk ratios (RRs), or odds ratios (ORs): all-time points as defined by the specific studies.

Data extraction, (selection and coding)
Two independent investigators will review titles and abstracts of bibliographic database search results as well as records from the trial registers to identify studies. Citations determined potentially eligible by either investigator will undergo full-text screening. A third party will verify the accuracy.

Data will be collected based on qualitative and quantitative information as the following:
(i) Patient characteristics (e.g., age, gender, ethnicity, and comorbidity)
(ii) Study characteristics (e.g., number of participants, study method, study location, sample size, setting, outcome measures, risk of bias, and industry sponsorship)
(iii) Thiazide diuretics exposure (e.g., treatment regimen, concomitant treatments, and duration of treatment)
(iv) Outcomes of interest (methods/definitions for assessment outcomes, specific skin cancer types, and pre-specified outcomes)

If studies had overlapping study populations, the study with the most detailed and relevant information will be used. For studies with missing data or uncertain information, the corresponding author will be contacted. If the authors do not respond, the study will be excluded. The final set of data will be cross-checked by the two investigators. Any discrepancies will be addressed through a discussion.

Risk of bias (quality) assessment
This systematic review and meta-analysis will be performed in line with the Preferred Reporting Items for Systematic Reviews and Meta-Analyses guidelines (PRISMA) and reported in line with the Meta-analysis Of Observational Studies in Epidemiology (MOOSE) statement.

Two independent investigators will be appraised the risk of bias and methodological quality for each included study based on its study design. The instrument to assess the risk of bias for RCTs and non-

randomized studies using the Version 2 of the Cochrane risk-of-bias tool for randomized trials (RoB 2) by The Cochrane Collaboration and the Newcastle-Ottawa Scale, respectively.

Strategy for data synthesis
Only studies published in full-text will be included in the data analysis to limit incomplete information, however, the unpublished studies or abstract from conference meetings will be included in the post hoc meta-analysis.

If applicable, the ~~relative risk (RRs)~~ odds ratios (ORs) with the greatest degree of adjustment for potential confounding factors will be analyzed as the common effect estimates of association across studies. The pooled ~~RRs~~ ORs along with the 95% confidence intervals (CIs) will be calculated using DerSimonian-Laird random-effects models.

Heterogeneity will be assessed using the chi-squared statistic, I^2 index, and tau-square statistic to estimate the degree of inconsistency. From the clinical point of view, heterogeneity of the studies will be done by qualitatively assessing the PICOTS (populations, interventions, comparators, outcomes, timing, and setting) of the included studies, looking for similarities and differences. The funnel plot and tested for funnel asymmetry using Begg's and Egger's regression test will be used to investigate any evidence of publication bias. The trim and fill method will be tested to calibrate for publication bias.

To explore the potential source of heterogeneity, the level risk of bias and key characteristics of included studies will be included in a random-effects meta-regression. Moreover, if possible, dose- and duration-responses effects will be also performed.

Analysis of subgroups or subsets
Preplanned stratification or categories for subgroup analyses include (i) patient characteristics (e.g., age, gender, race/ethnicity, individual thiazide diuretics use, skin phototype, and history of skin cancer or precancerous lesions) and (ii) study characteristics (e.g., randomized vs. non-randomized, sample size, duration of follow-up, study quality, and geographical location).

File S4 The Newcastle-Ottawa Scale (NOS) for Assessing the Quality of Non-Randomized Studies

NOS Assessment Scale: Case-Control Studies

Note: A study can be awarded a maximum of one star for each numbered item within the Selection and Exposure categories. A maximum of two stars can be given for Comparability.

Selection

1) Is the case definition adequate?
   - a) yes, with independent validation (histological confirmation or through regional/national cancer registries) *
   - b) yes, e.g., record linkage or based on self-reports
   - c) no description

2) Representativeness of the cases
   - a) consecutive or obviously representative series of cases *
   - b) potential for selection biases or not stated

3) Selection of Controls
   - a) community controls (population-based studies or nested case-control) *
   - b) hospital controls
   - c) no description

4) Definition of Controls
   - a) no history of disease (endpoint) *
   - b) no description of source

Comparability

1) Comparability of cases and controls on the basis of the design or analysis
   - a) study controls for “age and Fitzpatrick skin type/sun exposure” *
   - b) study controls for any additional factor: at least 3 additional factors, including sex, race/ethnic, skin tanning, smoking, alcohol consumption, precancerous skin conditions, comorbidities, photosensitizing drugs, medications with potential antineoplastic properties (e.g., aspirin, NSAIDs, statins), other antihypertensive agents, human papillomavirus; immunosuppressive drugs, environmental hazards/occupational risks (e.g., exposure of arsenic, polycyclic aromatic hydrocarbons, nitrosamine, alkylating agents), and genetic predisposing factors (e.g., xeroderma pigmentosum) *

Exposure

1) Ascertainment of exposure
   - a) secure record (e.g., prescription claim) *
   - b) structured interview where blind to case/control status *
   - c) interview not blinded to case/control status
   - d) written self-report or medical record only
   - e) no description

2) Same method of ascertainment for cases and controls
   - a) yes *
   - b) no

3) Non-Response rate
   - a) same rate for both groups *
   - b) non respondents described
   - c) rate different and no designation

File S4 The Newcastle-Ottawa Scale (NOS) for Assessing the Quality of Non-Randomized Studies (Continued)

NOS Assessment Scale: Cohort Studies

Note: A study can be awarded a maximum of one star for each numbered item within the Selection and Outcome categories. A maximum of two stars can be given for Comparability

Selection

1) Representativeness of the exposed cohort
    - a) truly representative: population-based cohort or regional/national registries data *
    - b) somewhat representative: hospital-based cohort *
    - c) selected group of users e.g., volunteers
    - d) no description of the derivation of the cohort

2) Selection of the non-exposed cohort
    - a) drawn from the same community as the exposed cohort *
    - b) drawn from a different source
    - c) no description of the derivation of the non-exposed cohort

3) Ascertainment of exposure
    - a) secure record (e.g., prescription claim) *
    - b) structured interview *
    - c) written self-report
    - d) no description

4) Demonstration that outcome of interest was not present at start of study
    - a) yes *
    - b) no

Comparability

1) Comparability of cohorts on the basis of the design or analysis
    - a) study controls for “age and Fitzpatrick skin type/sun exposure” *
    - b) study controls for any additional factor: at least 3 additional factors, including sex, race/ethnic, skin tanning, smoking, alcohol consumption, precancerous skin conditions, comorbidities, photosensitizing drugs, medications with potential antineoplastic properties (e.g., aspirin, NSAIDs, statins), other antihypertensive agents, human papillomavirus; immunosuppressive drugs, environmental hazards/occupational risks (e.g., exposure of arsenic, polycyclic aromatic hydrocarbons, nitrosamine, alkylating agents), and genetic predisposing factors (e.g., xeroderma pigmentosum) *

Outcome

1) Assessment of outcome
    - a) independent blind assessment *
    - b) record linkage (e.g., histological confirmation or through regional/national cancer registries, ICD9/10, or validated read codes) *
    - c) self-report
    - d) no description

2) Was follow-up long enough for outcomes to occur
    - a) yes (select an adequate follow up period for outcome of interest: median follow-up time >1 years or study period >5 years) *
    - b) no

3) Adequacy of follow up of cohorts
    - a) complete follow up - all subjects accounted for *
    - b) subjects lost to follow up unlikely to introduce bias (missing outcome <20%, select an adequate 80%) follow up, or description provided of those lost *
    - c) follow up rate <80% (missing outcome >20%) and no description of those lost
    - d) no statement

File S5 Modified Criteria of Evidence Certainty Assessment

Domain	Score and application
Study limitations	• Score as one of three levels, separately by type of study design:<br>○ Low level of study limitations<br>○ Medium level of study limitations<br>○ High level of study limitations
Directness	• Score as one of two levels:<br>○ Direct<br>○ Indirect
Consistency	• Score as one of three levels:<br>○ Consistent<br>○ Inconsistent<br>○ Unknown (e.g., single study)
Precision	• Score as one of two levels:<br>○ Precise<br>○ Imprecise
Reporting bias	• Score as one of two levels:<br>○ Suspected<br>○ Undetected
Dose-response association	• Score as one of two levels:<br>○ Present<br>○ Undetected
Plausible confounding that would decrease observed effect	• Score as one of two levels:<br>○ Present<br>○ Absent
Strength of association (magnitude of effect)	• Score as one of two levels:<br>○ Strong<br>○ Weak

Quality of the evidence

High quality: further research is very unlikely to change our confidence in the estimate of effect

Moderate quality: further research is likely to have an important impact on our confidence in the estimate of effect and may change the estimate

Low quality: further research is very likely to have an important impact on our confidence in the estimate of effect and is likely to change the estimate

Very low quality: we are very uncertain about the estimate.

Insufficient: we have no evidence, we are unable to estimate an effect, or we have no confidence in the estimate of effect for this outcome

Established an overall SOE grade:

- Levels of grades are intended to communicate reviewer’s conclusions of the strength of the overall assessment of a body evidence for a single outcome of a single treatment comparison.
- Each grade has two components. The first, principle definition concerns the level of confidence that reviewers place in the estimates for the benefit or harm; this equates to their judgment as to how closely the evidence is likely to reflect a true effect. The second, subsidiary definition involves an assessment of the level of deficiencies in the body of evidence and belief in the stability of the findings.
- The grade is based on domain scores as well as more holistic, summary appreciation of the possibly complex interaction among the individual domains.
- Evidence based on observational studies is assumed to pose a greater risk of having study limitation; this usually corresponding to an initial provisional grade of low SOE.
- Reviewers may also decide that after assessing the additional domains, the overall SOE of a body of observational studies can be upgraded to moderate (although rarely high).

Abbreviations: SOE, strength of evidence.

File S6 List of Excluded Articles

	Article	Reason for Exclusion
1	Sjöberg T, et al. Angiotensin-Converting Enzyme Inhibitors and Risk of Esophageal and Gastric Cancer: A Nested Case-Control Study. Clin Gastroenterol Hepatol. 2007;5(10):1160-1166.e1161.	No outcomes of interest reported
2	Veronesi M, et al. A prospective evaluation of persistence on antihypertensive treatment with different antihypertensive drugs in clinical practice. Vasc Health Risk Manag . 2007;3(6):999-1005.	Not relevant in the context of the use of thiazide diuretics and risk of skin cancer
3	Christian JB, et al. Association of ACE inhibitors and angiotensin receptor blockers with keratinocyte cancer prevention in the randomized VATTC trial. J Natl Cancer Inst. 2008;100(17):1223-1232.	Unclear definition of thiazide diuretics exposure
4	Jensen AO, et al. Use of photosensitizing diuretics and risk of skin cancer: A population based case control study. Pharmacoepidemiol Drug Saf. 2008;17:S109-S109.	Duplicate data
5	Coogan PF, et al. Diuretic use and the risk of breast cancer. J Hum Hypertens. 2009;23(3):216-218.	No outcomes of interest reported
6	Friedman GD, et al. Screening pharmaceuticals for possible carcinogenic effects: initial positive results for drugs not previously screened. Cancer Causes Control. 2009;20(10):1821-1835.	Duplicate data
7	Ruiter R, et al. High-Ceiling Diuretics Are Associated with an Increased Risk of Basal Cell Carcinoma in a Population-Based Follow-Up Study. Pharmacoepidemiol Drug Saf. 2010;19:S147-S148.	Duplicate data
8	Hirose H, et al. Effects of losartan/hydrochlorothiazide treatment, after change from ARB at usual dosage, on blood pressure and various metabolic parameters including high-molecular weight adiponectin in Japanese male hypertensive subjects. Clin Exp Hypertens. 2011;33(1):41-46.	Not relevant in the context of the use of thiazide diuretics and risk of skin cancer
9	Oliva S, et al. Administration of angiotensin-converting enzyme inhibitors and β-blockers during adjuvant trastuzumab chemotherapy for nonmetastatic breast cancer: Marker of risk or cardioprotection in the real world? Oncologist. 2012;17(7):917-924.	Not relevant in the context of the use of thiazide diuretics and risk of skin cancer
10	De Giorgi V, et al. Effect of beta-blockers and other antihypertensive drugs on the risk of melanoma recurrence and death. Mayo Clin Proc. 2013;88(11):1196-1203.	Not relevant in the context of the use of thiazide diuretics and risk of skin cancer
11	Jahan-Tigh RR, et al. Hydrochlorothiazide and cutaneous T cell lymphoma: Prospective analysis and case series. Cancer. 2013;119(4):825-831.	Case series
12	Gómez-Bernal S, et al. Photosensitivity due to thiazides. Actas Dermosifiliogr. 2014;105(4):359-366.	Review article
13	Johannesdottir SA, et al. Use of antihypertensive drugs and risk of skin cancer. Pharmacoepidemiol Drug Saf. 2014;1):375.	Duplicate data
14	McDonald E, et al. Prescription diuretic use and risk of basal cell carcinoma in the nationwide U.S. radiologic technologists cohort. Cancer Epidemiol Biomarkers Prev. 2014;23(8):1539-1545.	Unclear definition of thiazide diuretics exposure
15	Arnspang S, et al. Statin use and risk of nonmelanoma skin cancer: A nationwide study in Denmark. Br J Cancer. 2015;112(1):153-156.	Not relevant in the context of the use of thiazide diuretics and risk of skin cancer

Appendix VI List of Excluded Articles (Continued)

	Article	Reason for Exclusion
16	Goldvaser H, et al. The Association between Angiotensin Receptor Blocker Usage and Breast Cancer Characteristics. Oncology. 2016;91(4):217-223.	Not relevant in the context of the use of thiazide diuretics and risk of skin cancer
17	Gómez-Acebo I, et al. The use of antihypertensive medication and the risk of breast cancer in a case-control study in a Spanish population: The MCC-Spain study. PLoS ONE. 2016;11(8).	No outcomes of interest reported
18	Pottegard A, et al. Use of hydrochlorothiazide and risk of skin cancer. Pharmacoepidemiol Drug Saf. 2017;26 (Supplement 2):473.	Duplicate data
19	Sigaroudi A, et al. Comparison of hydrochlorothiazide and ramipril concentrations in simultaneous cerebrospinal fluid and blood serum samples. Naunyn-Schmiedeberg's Archives of Pharmacology. 2017;390 (Supplement 1):S9.	Not relevant in the context of the use of thiazide diuretics and risk of skin cancer
20	Mazzilli S, et al. Effects of topical 0.8% piroxicam and 50+ sunscreen filters on actinic keratosis in hypertensive patients treated with or without photosensitizing diuretic drugs: an observational cohort study. Clin Cosmet Investig Dermatol. 2018;11:485-490.	Not relevant in the context of the use of thiazide diuretics and risk of skin cancer
21	Pedersen SA, et al. Hydrochlorothiazide use and risk of merkel cell carcinoma and malignant adnexal skin tumours. Pharmacoepidemiol Drug Saf. 2018;27:388-389.	Duplicate data
22	Schmutz JL, et al. Hydrochlorothiazide and skin cancer. Ann Dermatol Venereol. 2018;145(3):225-226.	Opinion
23	Schmutz JL, et al. Hydrochlorothiazide appears to increase risk of melanoma. Annales de Dermatologie et de Venereologie. 2018;145(10):643-644.	Opinion
24	Crow LD, et al. Medications Associated with Increased Risk of Keratinocyte Carcinoma. Dermatologic Clinics. 2019;37(3):297.	Review article
25	Faconti L, et al. Hydrochlorothiazide and the risk of skin cancer. A scientific statement of the British and Irish Hypertension Society. J Hum Hypertens. 2019;33(4):257-258.	Commentary
26	Geyer S, et al. Hydrochlorothiazide and nonmelanoma skin cancer. Hautarzt. 2019;70(2):148-149.	Commentary
27	Haberle M, et al. Hydrochlorothiazide for lowering the Blood Pressure: What is the Relevance of this Medication to the Management of Squamous Cell Carcinoma? J Dtsch Dermatol Ges. 2019;17:85-86.	Not relevant in the context of the use of thiazide diuretics and risk of skin cancer
28	Kreutz R, et al. Reviewing the effects of thiazide and thiazide-like diuretics as photosensitizing drugs on the risk of skin cancer. J Hypertens 2019; 37(10): 1950-8.	Review article
29	Morales DR. et al. Association between hydrochlorothiazide exposure and skin, lip and oral cancer: A series of population-based nested case-control studies. Pharmacoepidemiol Drug Saf. 2019;28 (Supplement 2):42-43.	Duplicate data

Appendix VI List of Excluded Articles (Continued)

	Article	Reason for Exclusion
30	Olde Engberink RHG, et al. Hydrochlorothiazide and skin cancer. Nederlands Tijdschrift voor Geneeskunde. 2019;163(19).	Review article
31	Queen D, et al. Characteristics of non-melanoma skin cancers of the cutaneous perioral and vermilion lip treated by Mohs micrographic surgery. J Eur Acad Dermatol Venereol. 2019;33(2):305-311.	Not relevant in the context of the use of thiazide diuretics and risk of skin cancer
32	Santala EEE, et al. Antihypertensive drugs and prostate cancer survival after radical prostatectomy in Finland-A nationwide cohort study. Int J Cancer. 2019;144(3):440-447.	No outcomes of interest reported
33	Sokol G, et al. Geriatric Skin Cancer and Concomitant Photosensitivity Drug Utilization. J Geriatr Oncol. 2019;10 (6 Supplement 1):S36-S37.	Not relevant in the context of the use of thiazide diuretics and risk of skin cancer
34	Van Der Heijden S, et al. Hydrochlorothiazide and skin cancer. Warning for discussion? Geneesmiddelenbulletin. 2019;53(3).	Commentary
35	Wenzel RR, et al. Antihypertensive therapy & cancer. Journal Fur Hypertonie. 2019;23(1):8-17.	Review article
36	Daniels B, et al. Risks of squamous cell carcinoma of the lip and cutaneous melanoma in older Australians using hydrochlorothiazide (HCTZ). Pharmacoepidemiol Drug Saf. 2020;29:37-38.	Duplicate data
37	Eworuke E, et al. Risk of non-melanoma skin cancer associated with hydrochlorothiazide-containing products in the United States. Pharmacoepidemiol Drug Saf. 2020;29:9-9.	Duplicate data
38	Habel LA, et al. Hydrochlorothiazide and risk of melanoma subtypes. Pharmacoepidemiol Drug Saf. 2020;29:49-50.	Duplicate data
39	Hofmann GA, et al. The frequency of photosensitizing drug dispensings in Austria and Germany: a correlation with their photosensitizing potential based on published literature. J Eur Acad Dermatol Venereol. 2020;34(3):589-600.	Not relevant in the context of the use of thiazide diuretics and risk of skin cancer
40	Humbert X, et al. Thiazides and nonmelanoma skin cancer: Is it a class effect? New York, New York: Elsevier B.V.; 2020. p. e25-e6.	Letter to editor
41	Knuutila JS, et al. Risk factors and prognosis for metastatic cutaneous squamous cell carcinoma: A cohort study. Acta Derm Venereol. 2020;100(16):1-9.	Not relevant in the context of the use of thiazide diuretics and risk of skin cancer
42	Kristensen KB, et al. Use of antiepileptic drugs and risk of skin cancer: A nationwide case-control study. J Am Acad Dermatol. 2020;82(2):326-335.	Not relevant in the context of the use of thiazide diuretics and risk of skin cancer
43	Leon-Munoz LM, et al. Use of hydrochlorothiazide and risk of skin cancer in a large nested case-control study in Spain. Pharmacoepidemiol Drug Saf. 2020;29:573-573.	Duplicate data
44	Letellier T, et al. Hydrochlorothiazide exposure increases the risk of long-term squamous cell carcinoma after kidney transplantation. Transplant International. 2020;33 (Supplement 1):6-7.	Duplicate data

Appendix VI List of Excluded Articles (Continued)

	Article	Reason for Exclusion
45	Mazzilli S, et al. Efficacy of topical piroxicam 0.8% and sunscreen 50+ on actinic keratosis lesions in hypertensive subjects with or without thiazides diuretic treatments. Journal of the Dermatology Nurses' Association Conference: 24th World Congress of Dermatology Milan Italy 2020; 12(2).	Not relevant in the context of the use of thiazide diuretics and risk of skin cancer
46	Oh CC, et al. Coffee, tea, caffeine, and risk of non melanoma skin cancer in a chinese population: the singapore chinese health study. Journal of the Dermatology Nurses' Association Conference: 24th World Congress of Dermatology Milan Italy 2020; 12(2).	Not relevant in the context of the use of thiazide diuretics and risk of skin cancer
47	O'Neill B, et al. Hydrochlorothiazide and squamous cell carcinoma. Canadian Family Physician 2020; 66(2): 116.	Opinion
48	Pease DR, et al. Cutaneous T-cell lymphoma after chronic exposure to hydrochlorothiazide: pharmacovigilance analysis from the RADAR (Research on Adverse Drug events And Reports) Program. J Eur Acad Dermatol Venereol. 2020.	Letter to editor
49	Pottegard A, et al. Use of hydrochlorothiazide in Denmark following publication of skin cancer risk findings. Pharmacoepidemiol Drug Saf. 2020;29:141-141.	Not relevant in the context of the use of thiazide diuretics and risk of skin cancer
50	Pottegard A, et al. Use of hydrochlorothiazide and risk of uveal melanoma. Pharmacoepidemiol Drug Saf. 2020;29:573-573.	No outcomes of interest reported
51	Rouette J, et al. Use of hydrochlorothiazide and risk of melanoma and nonmelanoma skin cancer. Pharmacoepidemiol Drug Saf. 2020;29:570-570.	Duplicate data
52	Schneider R, et al. Risk of skin cancer in new users of thiazides and thiazide-like diuretics: A cohort study using an active comparator group. Pharmacoepidemiol Drug Saf. 2020;29:568-568.	Duplicate data
53	Schulz M, et al. Impact on antihypertensive prescribing after the dear healthcare professional letter on increased risk of skin cancer related to hydrochlorothiazide. Value Health. 2020;23:S102-S102.	Not relevant in the context of the use of thiazide diuretics and risk of skin cancer
54	Tironneau S, et al. Increased risk of skin cancer with hydrochlorothiazide: What are the practical consequences? Prescrire Int. 2020;29(217):186-187.	Review article
55	Warszawik-Hendzel O, et al. Cardiovascular Drug Use and Risk of Actinic Keratosis: A Case-Control Study. Dermatol Ther (Heidelb). 2020;10(4):735-743.	Unadjusted effect estimates
56	Copland E, et al. Antihypertensive treatment and risk of cancer: an individual participant data meta-analysis. Lancet Oncol. 2021;22(4):558-570.	Insufficient data
57	Lecaros-Astorga, et al. Hydrochlorothiazide use and risk of non-melanoma skin cancer in Spain: A case/non-case study. Int J Clin Pharmacol ther. 2021;59(4):280-8.	Unadjusted effect estimates
58	Rodríguez-Jiménez P, et al. RF - Thiazide Diuretics and Nonmelanoma Skin Cancer. Actas Dermo-Sifiliograficas 2021; 112(2): 176-7.	Opinion
